# Novel spirocyclic tranylcypromine derivatives as lysine-specific demethylase 1 (LSD1) inhibitors†

**DOI:** 10.1039/c7ra13097j

**Published:** 2018-01-05

**Authors:** Ying Shi, Yan-Ran Wu, Ming-Bo Su, Dong-Hao Shen, Hendra Gunosewoyo, Fan Yang, Jia Li, Jie Tang, Yu-Bo Zhou, Li-Fang Yu

**Affiliations:** Shanghai Engineering Research Center of Molecular Therapeutics and New Drug Development, School of Chemistry and Molecular Engineering, East China Normal University 3663 North Zhongshan Road Shanghai 200062 China lfyu@sat.ecnu.edu.cn +86-021-622-31385; CAS Key Laboratory of Receptor Research, National Center for Drug Screening, Shanghai Institute of Materia Medica, Chinese Academy of Sciences 189 Guo Shou Jing Road Shanghai 201203 China ybzhou@simm.ac.cn +86-21-508-01313; School of Pharmacy, Faculty of Health Sciences, Curtin University Bentley Perth WA 6102 Australia; Shanghai Key Laboratory of Green Chemistry and Chemical Process, School of Chemistry and Molecular Engineering, East China Normal University 3663 North Zhongshan Road Shanghai 200062 China

## Abstract

Herein we describe the design, synthesis, and biological evaluation of a novel series of tranylcypromine-based LSD1 inhibitors *via* conformational restriction using spiro ring systems. A simple, direct spirocyclic analog of tranylcypromine (compounds 8a and 8b) was shown to be a 28- to 129-fold more potent inhibitor of LSD1 enzyme compared to tranylcypromine. Further incorporation of various substituted benzyl groups to the amino group resulted in a suite of 2′,3′-dihydrospiro[cyclopropane-1,1′-inden]-2-amines that are potent LSD1 inhibitors with excellent selectivity profiles (*e.g.*14a, 15b, 16a, 19a and 20b) against closely related enzymes such as MAO-A, MAO-B, and LSD2.

## Abstract 2

Herein we describe the design, synthesis, and biological evaluation of a novel series of tranylcypromine-based LSD1 inhibitors *via* conformational restriction using spiro ring systems.

## Graphical abstract

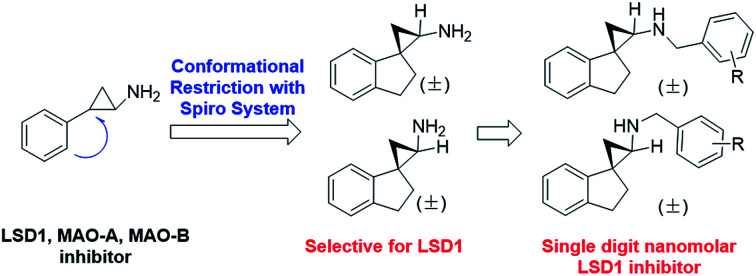

## Introduction

Epigenetic modifications of DNA or histone tails, such as methylation and acetylation, provide regulatory mechanisms to influence gene expression. This dynamic process allows for the chromatin uncoiling or compaction, thereby altering the ability of the transcription machineries to access the DNA. Lysine-specific demethylase 1 (LSD1) is now recognized as the first true histone demethylase, originally discovered by the group of Shi Yang in 2004.^1^ LSD1 belongs to the flavin adenine dinucleotide (FAD)-dependent amine oxidase superfamily and specifically demethylates mono- and dimethylated histone H3 lysine 4 (H3K4me and H3K4me2).^1,2^ The catalytic cycle is thought to involve the methylated lysine being firstly oxidized by FAD co-factor to form an imine intermediate, followed by hydrolysis to generate formaldehyde and the demethylated lysine. FADH_2_, the reduced product of FAD, is then oxidized by O_2_ to form FAD and H_2_O_2_. LSD1 is often present in transcriptional co-repressor complexes such as RE1-silencing transcriptional factor corepressor 1 (CoREST), which can stabilize and recruit LSD1 after binding to chromatin.^3^ Alteration of the LSD1 substrate specificity occurs upon association with the androgen receptor, namely from H3K4 to H3K9.^4^ LSD1 is also known for its ability to demethylate non-histone proteins, such as p53, thus inhibiting the apoptosis process mediated by p53.^5^ In addition to LSD1, the LSD family has a homologue known as LSD2 which was first indentified in 2009.^6,7^ Both homologues contain the N-terminal SWIRM (Swi3p, Rsc8p and Moira) domain and the C-terminal amine oxidase domain.^8^ However, while LSD1 contains the Tower domain which forms a binding site with CoREST, the LSD2 contains a CW-type zinc finger domain with unknown function.^6^

There are numerous studies reporting that LSD1 is upregulated in various tumor tissue cells and tissues including retinoblastoma,^9^ non-small cell lung cancer,^10^ prostate cancer,^11^ breast cancer,^12,13^ and colon cancer.^14^ LSD1 is also overexpressed in MLL-rearranged leukemia as a key regulator to promote the oncogenic potential of MLL-AF9 leukemia stem cell.^15^ MLL gene translocation is a major biomarker in acute leukemia which is typically associated with a poor prognosis. The results of RNAi-mediated knockdown or pharmacological inhibition of LSD1 seem to suggest that this enzyme induces re-expression of epigenetically silenced tumor suppressor genes and regulates p53 transcriptional activity or down-regulation of several leukemic-related genes.^15–17^ Overall, LSD1 represents a promising target for the treatment of leukemia as well as other solid tumors.

**Fig. 1:**
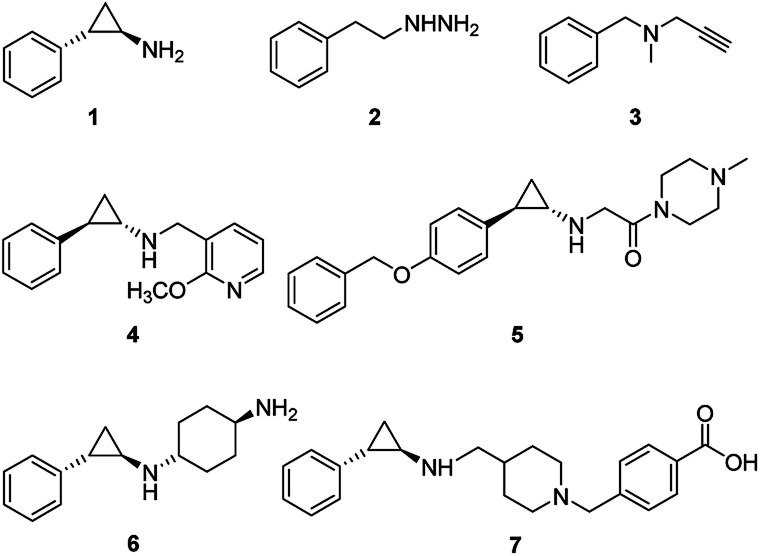
Structures of irreversible LSD1 inhibitors.

**Fig. 2:**
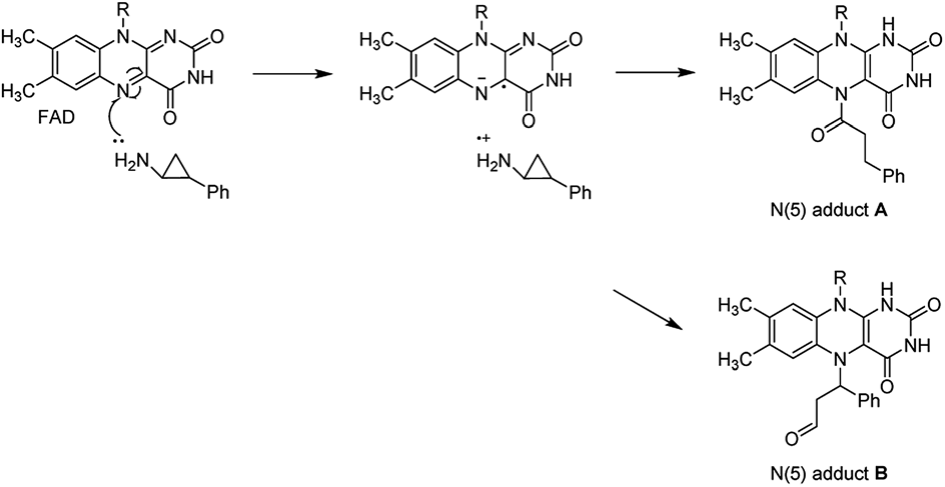
Proposed mechanism of inactivation of LSD1 by tPCPA.

LSD1 exhibits homology with monoamine oxidases (MAOs) A and B with 17.6% identity.^18^ Classic MAO inhibitors such as *trans*-phenylcyclopropylamine (1, tPCPA), phenelzine (2), and pargyline (3) were found to show LSD1 inhibitory activity by forming covalent adduct with FAD (Fig. 1). The (1*R*, 2*S*)-isomer of 1 obtained by chiral resolution of tPCPA reacts with FAD in the active site to produce *N*-(5) adduct A, while the (1*S*, 2*R*)-isomer generates *N*-(5) adduct B (Fig. 2).^18,19^ A number of tPCPA-containing inhibitors were developed with the aim to improve the potency at LSD1 as well as the selectivity over MAOs. The catalytic site for LSD1 is larger compared to MAOs,^20^ with substitutions at the phenyl ring and amino nitrogen atom generally increasing the LSD1 inhibitory activity. Compound 4 is a potent LSD1 inhibitor with an IC_50_ value of 98 nM that has been demonstrated to inhibit colony forming capacity of MLL-AF9 leukemia cells.^15^ Compound 5 was reported to be a brain-permeable LSD1 inhibitor that can block memory consolidation in a mouse model.^21^ Other classes of reversible LSD1 inhibitors have also been reported, including peptides, polyamine analogues, pyrimidine-thioureas, 3-(piperidin-4-ylmethoxy)pyridine and 3,5-diamino-1,2,4-triazoles.^22–27^*N*1-[(1*R*, 2*S*)-2-phenylcyclopropyl]cyclohexane-1,4-diamine dihydrochlorideORY-1001 (6, ORY1001, RG6016, Oryzon Genomics) is an irreversible LSD1 inhibitor that was granted an orphan drug status for the treatment of acute myeloid leukemia (AML) in phase IIA clinical trial.^28^ GlaxoSmithKline developed a potent LSD1 inhibitor, compound 7 (GSK2879552), which is currently in the phase I trials for treatment of relapsed/refractory small cell lung cancer and AML. More recently, Oryzon Genomics announced tPCPA analogue ORY2001, a dual LSD1/MAO-B inhibitor, that was advanced in phase I studies for Alzheimer's disease in 2016. Other compounds currently in phase I/II clinical trials for oncology include INCB059872, IMG-7289 and CC-90011.^29^

**Fig. 3:**
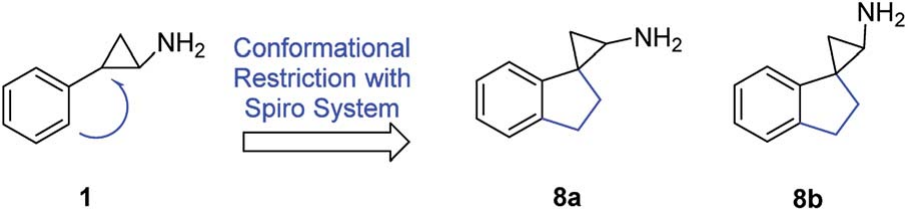
Conformationally constrained compounds 8a and 8b.

Although there are numerous medicinal chemistry campaigns on tranylcypromine-related analogues,^30^ thus far there has been a limited report on the incorporation of spirocycle into the system.^31,32^ Spiro containing system has been increasingly utilized in medicinal chemistry as it introduces not only diverse orientation but also structural novelty.^33^ Within this context, the spirocycle constraint may influence the potency and selectivity for LSD1 inhibition over other homologous enzymes, such as LSD2, MAO-A, and MAO-B. In the present study, we report a series of 2′,3′-dihydrospiro[cyclopropane-1,1′-inden]-2-amine analogues and their potencies at inhibiting LSD1 and related enzymes (Fig. 3).

## Results and discussion

**Scheme 1:**
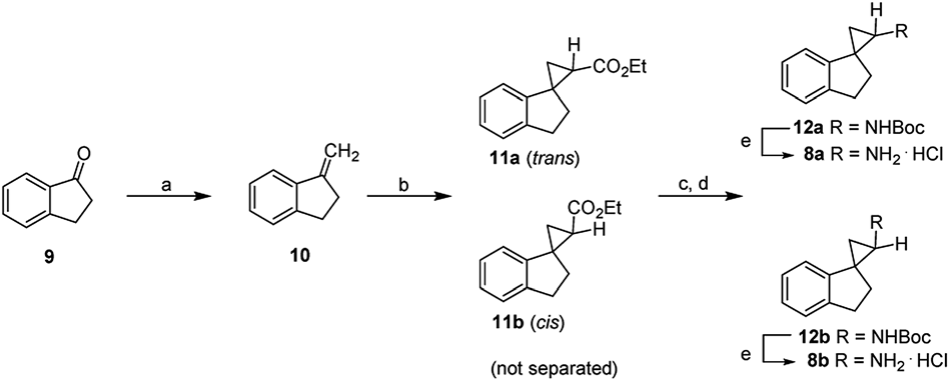
^*a*^Reagents and conditions: (a) methyltriphenylphosphonium bromide, ^*t*^BuOK, THF, rt; (b) Rh_2_(OAc)_4_ (cat.), N_2_CH_2_COOEt, CH_2_Cl_2_, 45 °C; (c) KOH, EtOH, 80 °C; (d) i. diphenylphosphoryl azide (DPPA), Et_3_N, toluene, 0 °C-25 °C; ii. ^*t*^BuOH, reflux; (e) 4 M HCl in ethyl acetate, rt.

Compounds 8a and 8b were first synthesized as outlined in Scheme 1, starting from the commercially available 2,3-dihydro-1*H*-inden-1-one (9). Terminal olefin 10 was prepared *via* Wittig reaction with methyltriphenylphosphonium bromide. Cyclopropanation with ethyl diazoacetate catalyzed by rhodium(ii) acetate dimer resulted in the esters 11a and 11b. Following basic hydrolysis, the corresponding carboxylic acids were in turn converted to the carbamates 12a and 12b*via* Curtius rearrangement using diphenylphosphoryl azide (DPPA), triethylamine and refluxing in anhydrous *tert*-butanol. The resultant enantiomers 12a and 12b were readily separated by flash chromatography and the stereochemical assignments were based collectively on their 1*H*,1*H* COSY and Nuclear Overhauser Effect (NOE) NMR spectra (ESI Table 1†). Briefly, for the aromatic ring of compound 8a, the H-5′ proton (*δ* 7.18–7.12) coupled with H-4′ (*δ* 7.22) and H-6′ (*δ* 7.18–7.12). Similarly, H-6′ proton (*δ* 7.18–7.12) coupled with H-5′ (*δ* 7.18–7.12) and H-7′ (*δ* 6.76). In the aliphatic region, H-3′ (*δ* 3.14) coupled with H-2′ (*δ* 2.35–2.20) and the H-2 (*δ* 2.88) with H-3 (*δ* 1.42, 1.29). For compound 8a, NOE correlations were observed between H-7′ and H-2. For compound 8b, in the aromatic region, the H-6′ proton (*δ* 7.26–7.21) coupled with H-5′ (*δ* 7.26–7.21) and H-7′ (*δ* 7.08–7.06). Similarly, the H-5′ proton (*δ* 7.26–7.21) coupled with H-4′ (*δ* 7.32) and H-6′ (*δ* 7.26–7.21). In the aliphatic region, the H-3′ (*δ* 3.18–3.10, 2.97) coupled with H-2′ (*δ* 2.38–2.30, 1.97). The H-2 proton (*δ* 2.88) coupled with H-3 (*δ* 1.51, 1.43). For compound 8b, NOE correlations were seen for H-2 and H-2′, and therefore supporting the stereochemical assignments of 8a as the *trans*-isomer and 8b as the *cis*-isomer. Lastly, deprotection of the Boc group in acidic conditions afforded final compounds 8a and 8b as hydrochloride salts.

Following their synthesis, compounds 8a and 8b were tested for their inhibitory activities against the purified LSD1 recombinant. As shown in Table 1, compounds 8a and 8b drastically improved the inhibitory activity against LSD1 by 129- and 28-fold compared to the control compound tPCPA (1), with the *trans*-isomer 8a being 5-fold more potent than the *cis*-isomer 8b. Furthermore, both isomers exhibited very high selectivity of >600- and >120- fold against MAO-A, but only modest selectivity for MAO-B. The observed trend suggested that the conformational restriction imposed by the spirocycle was beneficial for the inhibitory activity against LSD1 and selectivity over MAO-A.

**Table 1:** *In vitro* inhibitory activities against LSD1, MAO-A, MAO-B, and LSD2^a^

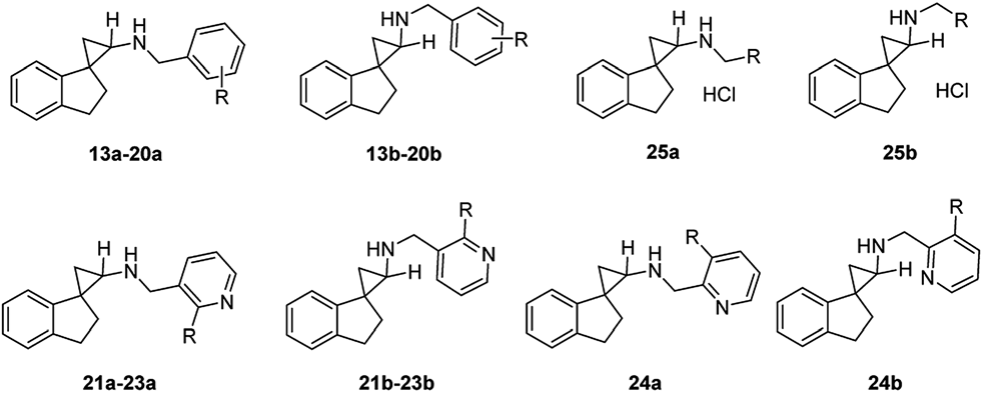				
Compound	LSD1, IC_50_ (μM)	MAO-A, IC_50_ (μM)	MAO-B, IC_50_ (μM)	LSD2, IC_50_ (μM)
1	22	11 ± 4	7.0 ± 0.8	NT^b^
6	0.014 ± 0.003	>100	194 ± 114	NT
8a	0.17 ± 0.01	>100	0.90 ± 0.09	NT
8b	0.78 ± 0.05	>100	7.8 ± 0.7	NT
13a	3.3 ± 0.6	NT	NT	NT
13b	0.11 ± 0.02	0.13 ± 0.01	5.1 ± 0.1	2.0 ± 0.2
14a	0.0022 ± 0.0003	5.9 ± 1.1	12 ± 1	NT
14b	0.0094 ± 0.0027	1.7 ± 0.3	70 ± 35	NT
15a	0.0070 ± 0.0017	4.8 ± 0.5	9.8 ± 2.3	NT
15b	0.0011 ± 0.0001	2.5 ± 0.1	32 ± 3	NT
16a	0.0095 ± 0.0024	3.7 ± 0.8	1.2 ± 0.2	6.8 ± 1.0
16b	0.063 ± 0.008	0.39 ± 0.19	7.7 ± 3.8	4.7 ± 0.8
17a	0.0066 ± 0.0015	0.92 ± 0.18	2.3 ± 0.4	0.80 ± 0.27
17b	0.017 ± 0.003	0.12 ± 0.01	27 ± 4	1.7 ± 0.5
18a	0.0071 ± 0.0013	2.2 ± 0.7	8.7 ± 1.1	0.66 ± 0.06
18b	0.015 ± 0.003	0.090 ± 0.010	31 ± 4	0.49 ± 0.10
19a	0.0030 ± 0.0001	11.7 ± 2.4	15 ± 2	NT
19b	0.012 ± 0.002	3.1 ± 0.2	27 ± 4	NT
20a	0.025 ± 0.007	1.4 ± 0.1	20 ± 1	2.3 ± 0.1
20b	0.0083 ± 0.0019	5.0 ± 1.8	7.0 ± 0.7	1.6 ± 0.2
21a	0.017 ± 0.003	10.8 ± 1.1	15 ± 1	NT
21b	0.020 ± 0.003	6.0 ± 1.1	28 ± 6	NT
22a	0.032 ± 0.006	>100	47 ± 5	NT
22b	0.010 ± 0.005	>100	68 ± 8	NT
23a	0.064 ± 0.001	9.7 ± 1.5	8.2 ± 1.5	13 ± 3
23b	0.070 ± 0.003	9.8 ± 0.8	29 ± 4	8.7 ± 0.8
24a	0.25 ± 0.01	NT	NT	NT
24b	0.47 ± 0.02	NT	NT	NT
25a	1.4 ± 0.1	NT	NT	NT
25b	22 ± 4	NT	NT	NT

a See Experimental section. Data represent mean values ± SEM of eight-point experiments each performed from three independent experiments. Positive controls: compound 1 and 6.

b NT: not tested.

**Scheme 2:**
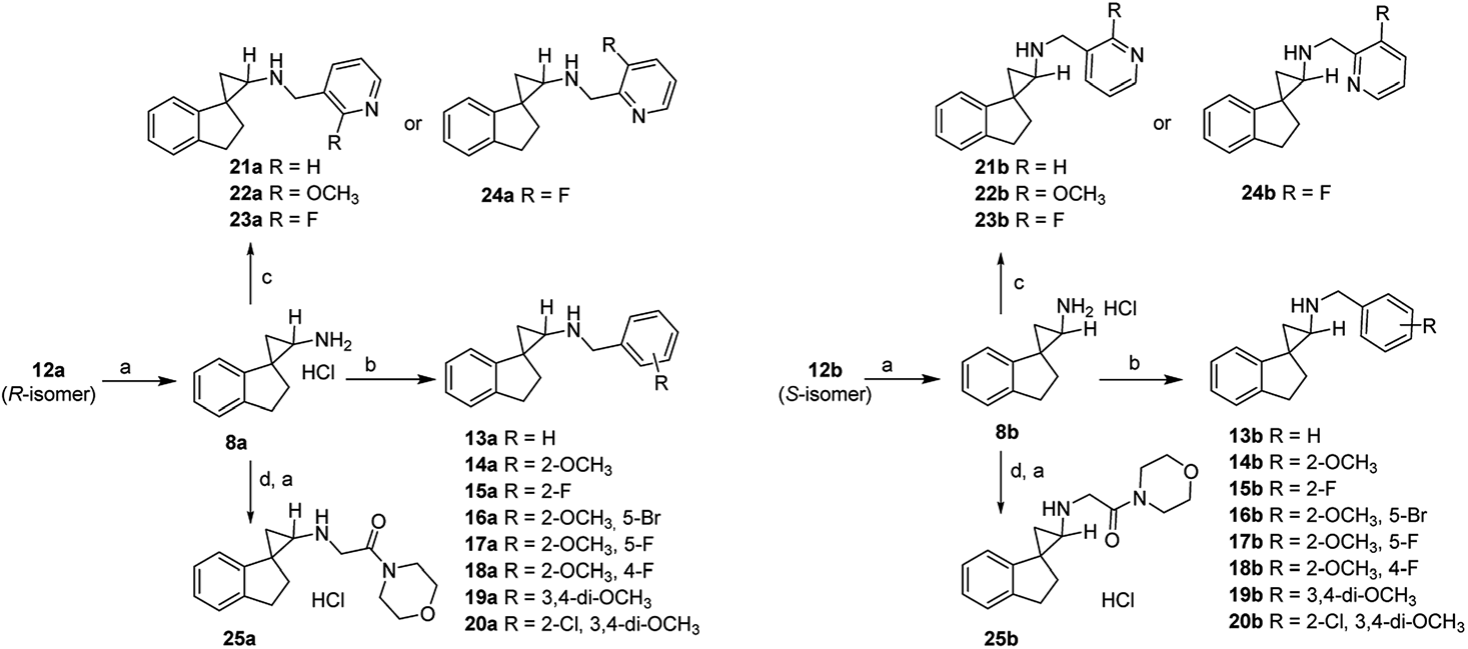
^*a*^Reagents and conditions: (a) 4 M HCl in ethyl acetate, rt; (c) substituted pyridine aldehyde, NaBH_4_, MeOH, rt; (b) substituted benzaldehyde, NaBH_4_, MeOH, rt; (d) 2-chloro-1-morpholinoethan-1-one, NaH, DMF, 0 °C–rt.

Encouraged by the preliminary *in vitro* results of the spirocycles 8a and 8b, we further explored structural extensions on the amino group (13a–25a, 13b–25b). As outlined in Scheme 2, reductive amination of the amines 8a and 8b with various substituted benzaldehydes or pyridine aldehydes using NaBH_4_ gave the final products 13a–24a and 13b–25b. Alkylation of 8a or 8b with 2-chloro-1-morpholinoethan-1-one in the presence of sodium hydride in anhydrous dimethylformamide (DMF) provided the amides 25a and 25b. Their IC_50_ values for the inhibition of LSD1, MAO-A, MAO-B and LSD2 are summarized in Table 1.

A benzyl substitution on the amino group of 8a resulted in a 19-fold decrease in the LSD1 inhibitory activity (13a*vs.*8a: IC_50_ = 3.3 μM *vs.* 0.17 μM), while the reverse trend was observed for 13b*vs.*8b (IC_50_ = 0.11 μM *vs.* 0.78 μM). To our delight, all the other synthesized benzyl and pyridylmethyl derivatives demonstrated potent inhibitory activities at the LSD1 with IC_50_ values between 1.1 nM and 0.47 μM (14a–24a, 14b–24b). Generally, the *trans*-isomers are more potent LSD1 inhibitors compared to the corresponding *cis*-isomers, with the exceptions of 13a*vs.*13b (IC_50_ = 3.3 μM *vs.* 0.11 μM), 15a*vs.*15b (IC_50_ = 7.0 nM *vs.* 1.1 nM), 20a*vs.*20b (IC_50_ = 25 nM *vs.* 8.3 nM), and 22a*vs.*22b (IC_50_ = 32 nM *vs.* 10 nM). In particular, a 2-methoxybenzyl (14a, 14b) or 2-fluorobenzyl substituents (15a, 15b) on the amino group enhanced the inhibitory potencies to single digit nanomolar levels, indicating that both electron donating and withdrawing groups are tolerated at the 2-position of the aromatic ring. Within the disubstituted benzyl analogues (16a–19a, 16b–19b), all the *trans*-isomers were found to be more potent inhibitors of LSD1 compared to their corresponding *cis*-isomers. Among the 2,5-disubstituted benzyls analogues 16a–17a and 16b–17b, while the *trans*-isomers are almost equipotent at LSD1, the additional 5-fluoro substitution gave slightly more potent compounds than the 5-bromo substitution (17b*vs.*16b) for the *cis*-isomers, indicating potential steric issues at this region. Moving the fluoro substituent from the 5- to the 4-position of the benzyl group did not elicit significant changes in the IC_50_ values (17a, 17b*vs.*18a, 18b). The 3,4-dimethoxy analogues (19a, 19b) were almost as equipotent as the 2-methoxy analogue (14a, 14b). For the trisubstituted benzyl analogue 20, the *cis*-isomer (20b) was found to be 3-fold more potent than the *trans*-isomer (20a). Changing a benzyl to a 3-pyridylmethyl group (21a–23a, 21b–23b) resulted in essentially equipotent isomers, with the exception of the 2-methoxy derivative, where the *trans*-isomer 22a (IC_50_ = 32 nM) was 3-fold less potent than the *cis*-isomer 22b (IC_50_ = 10 nM). The unsubstituted 3-pyridylmethyl analogues 21a and 21b were found to be 10-fold and 39-fold more potent than 8a and 8b, respectively. The 2-methoxy analogues 22a and 22b exhibited similar inhibitory activity against LSD1 compared with 21a and 21b. However, the 2-fluoro derivatives 23a and 23b were approximately 4-fold less potent than the unsubstituted derivatives 21a and 21b (IC_50_ = 64 and 70 nM). Keeping the fluoro group intact, a pyridyl walk from 3- to the 2-position (24a, 24b*vs.*23a, 23b) resulted in at least a 4-fold reduction in their LSD1 inhibitory potencies. Swapping the aromatic group to an aliphatic morpholinecarbonyl (25a, 25b) showed a further significant reduction in the LSD1 inhibitory activities, with IC_50_ values of 1.4 and 22 μM, respectively. In terms of selectivity for LSD1 inhibition over other related enzymes LSD2, MAO-A and MAO-B, all of the synthesized target compounds were the most potent at inhibiting LSD1 with selectivity index varying between low (*e.g.* IC_50_ values of 13b and 18b for LSD1 *vs.* MAO-A) and excellent (*e.g.* IC_50_ values of 14a, 15b, 16a, 19a and 20b for LSD1 *vs.* MAO-A, MAO-B and LSD2).

**Scheme 3:**
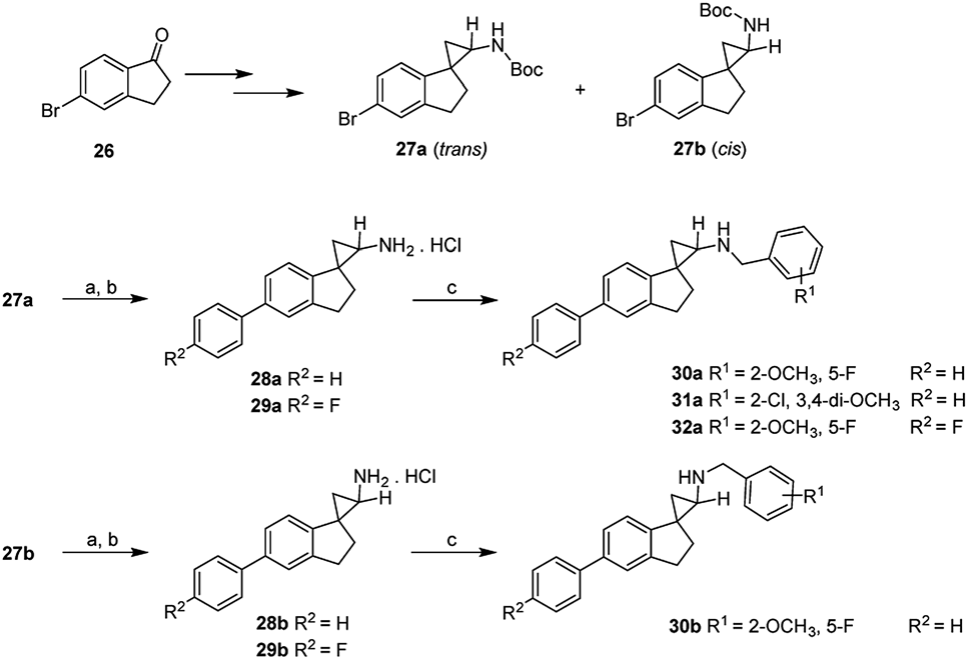
^*a*^Reagents and conditions: (a) Pd(PPh_3_)_4_ (cat.), boronic acid, Na_2_CO_3_, DMF, 80 °C; (b) 4 M HCl in EtOAc, rt; (c) substituted benzaldehyde, NaBH_4_, MeOH, rt.

The subsequent round of structural activity relationship (SAR) investigations involved structural extensions on the aromatic ring of the dihydroindene. As shown in Scheme 3, the commercially available 5-bromo-2,3-dihydro-1*H*-inden-1-one (26) were used as the starting material and subjected to the conditions as previously outlined in Scheme 2. The isomers 27a and 27b were separated by flash chromatography and the relative stereochemistry assigned based on a combination of their COSY and NOESY studies. Suzuki coupling of 27a with aromatic boronic acid in the presence of Pd(PPh_3_)_4_ at 80 °C in degassed 1 M Na_2_CO_3_ (aq)/DMF, followed by deprotection of the Boc group afforded the desired compounds 28a and 29a. Compounds 28b and 29b were synthesized following identical procedures with 27b as the starting material. The final compounds 30a–32a and 30b were readily accessed *via* reductive amination of the corresponding precursors with various substituted benzaldehydes and NaBH_4_ as the reducing agent. Their inhibitory activities at LSD1 and related enzymes are shown in Table 2.

**Table 2:** *In vitro* inhibitory activities of 28a–32a and 28b–30b against LSD1^a^

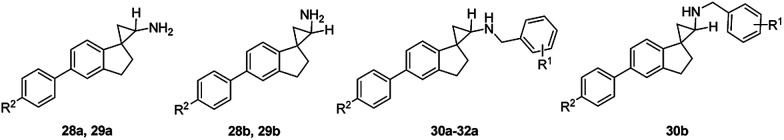			
Compound	*R* ^1^	*R* ^2^	LSD1, IC_50_ (μM)
1	—	—	22
8a	—	—	0.17 ± 0.01
8b	—	—	0.78 ± 0.05
28a	—	H	0.10 ± 0.01
28b	—	H	2.7 ± 0.2
29a	—	F	0.30 ± 0.01
29b	—	F	6.0 ± 0.8
30a	2-OCH_3_, 5-F	H	1.7 ± 0.1
30b	2-OCH_3_, 5-F	H	4.4 ± 0.3
31a	2-Cl,3,4-di-OCH_3_	H	8.9 ± 1.2
32a	2-OCH_3_, 5-F	F	2.6 ± 0.5

a See Experimental Section. Data represent mean values ± SEM of eight-point experiments each performed from three independent experiments. Positive control: compound 1.

As demonstrated in Table 2, an unsubstituted benzene ring extension at the aromatic group of the dihydroindene gave opposite effects depending on the stereochemistry of the cyclopropanamine. For the *trans*-isomers, a very slight increase in potency was observed (28a*vs.*8a), whereas an opposite trend was seen for the *cis*-isomers (28b*vs.*8b), with 28b having an IC_50_ value of 2.7 μM against LSD1. A 4-fluoro substitution on the newly added benzene ring resulted in approximately 3-fold reduction in the LSD1 inhibitory potencies (29a, 29b*vs.*28a, 28b). The remaining synthesized compounds 30a, 30b, 31a and 32a all showed IC_50_ values in the single digit micromolar range, indicating a potentially undesired steric effect at this region. As the most potent LSD1 inhibitor arising from this biphenyl series, compound 28a was further tested against other enzymes MAO-A, MAO-B and LSD2, with IC_50_ values of 0.46 μM, 0.17 μM and 94 μM, respectively.

## Conclusion

In summary, a novel series of 2′,3′-dihydrospiro[cyclopropane-1,1′-inden]-2-amine analogues as potent LSD1 inhibitors have been developed. In line with the conformational restriction conferred by the spirocycle to the tranylcypromine system, compounds 8a and 8b displayed significant improvements in their LSD1 inhibitory potencies and high selectivities for MAO-A. In order to further explore the SAR, structural modifications on the amino group and the benzene ring of the dihydroindene were found to give more potent compounds. Specifically, the addition of substituted benzyl moieties to the amino group (13a–25a, 13b–25b) showed single digit nanomolar potencies against LSD1 and excellent selectivity profiles against the homologous MAO enzymes and LSD2. While most of the *trans*-isomers were generally found to be more potent than the corresponding *cis*-isomers, there were some exceptions in the SAR data. The additional aromatic moiety at the benzene ring of the dihydroindene (28a–32a, 28b–30b) resulted in a significant drop of the LSD1 inhibitory potencies, indicating an unfavorable steric clash at this region. Overall, these studies warrant the further development of spirocyclic tranylcypromine derivatives as potent and selective LSD1 inhibitors.

## Experimental

### Chemistry

Starting materials, reagents, and solvents were purchased from commercial suppliers and used without further purification, unless otherwise stated. Anhydrous tetrahedrofuran (THF) and CH_2_Cl_2_ were obtained by distillation over sodium wire or CaH_2_, respectively. All non-aqueous reactions were run under a nitrogen atmosphere with exclusion of moisture from reagents, and all reaction vessels were oven-dried. The progress of reactions was monitored by TLC on SiO_2_. Spots were visualized by their quenching of the fluorescence of an indicator admixed to the SiO_2_ layer, or by dipping into phosphomolybdic acid ethanol solution followed by heating. SiO_2_ for flash chromatography was of 200–300 mesh particle size, and an EtOAc/PE mixture or gradient was used unless stated otherwise. ^1^H NMR spectra were recorded at a spectrometer frequency of 400 MHz, ^13^C NMR spectra at 101 MHz. Chemical shifts are reported in *δ* (ppm) using the *δ* 0 signal of tetramethylsilane (TMS) as internal standard. High resolution mass spectra were performed using a Bruker ESI-TOF high-resolution mass spectrometer and Waters Micromass Q-TOF micro Synapt high definition mass spectrometer.

#### 1-Methylene-2,3-dihydro-1*H*-indene (10)

To a solution of 2,3-dihydro-1*H*-inden-1-one (9, 4.00 g, 30.3 mmol) and methyltriphenylphosphonium bromide (12.97 g, 36.32 mmol) in THF (20 mL) was added potassium *tert*-butoxide (4.08 g, 36.32 mmol) in THF (20 mL) *via* an addition funnel over 0.5 h. The resulting mixture was stirred at room temperature for 16 h. The solvent was removed under vacuum and the residue was purified by flash chromatography on silica gel, eluting with PE/EtOAc (100 : 1) to provide the title compound 10. Colorless oil; yield 87%. ^1^H NMR (400 MHz, CDCl_3_) *δ* 7.49–7.47 (m, 1H), 7.25–7.23 (m, 1H), 7.21–7.16 (m, 2H), 5.45–5.43 (m, 1H), 5.02–5.01 (m, 1H), 2.96 (t, *J* = 7.0 Hz, 2H), 2.80–2.77 (m, 2H).

#### Ethyl 2′,3′-dihydrospiro[cyclopropane-1,1′-indene]-2-carboxylate (11a and 11b)

To a solution of 10 (3.42 g, 26.27 mmol) and rhodium(ii) acetate dimer (115 mg, 0.26 mmol) in refluxing CH_2_Cl_2_ (5 mL) was added ethyl diazoacetate (8.98 g, 78.81 mmol) in CH_2_Cl_2_ (2 mL) *via* syringe pump over 1 h. After refluxing for 3 h the resulting mixture was stirred at room temperature overnight. The solvent was removed under vacuum and the residue was purified by flash chromatography on silica gel, eluting with PE/EtOAc (100 : 1) to provide the title compounds 11a and 11b as a mixture. Colorless oil; total yield 59%. The diastereoisomers 11a and 11b were not isolated and used directly in the next reaction.

#### *tert*-Butyl ((2*R*)-2′,3′-dihydrospiro[cyclopropane-1,1′-inden]-2-yl)carbamate (12a) and *tert*-butyl ((2*S*)-2′,3′-dihydrospiro[cyclopropane-1,1′-inden]-2-yl)carbamate (12b)

To a stirred solution of 11a and 11b (3.35 g, 15.49 mmol) in EtOH (10 mL) was added KOH (2.61 g, 46.47 mmol). After refluxing for 3 h, the reaction was cooled to rt. EtOAc (20 mL) was added to the reaction mixture, followed by extraction with H_2_O (3 × 30 mL). A 10% aqueous hydrochloric acid solution was added to the combined aqueous layers until pH value dropped to 5–6, which was then extracted with EtOAc (3 × 30 mL). The combined organic layers were washed with brine (1 × 30 mL), dried over Na_2_SO_4_, filtered and concentrated *in vacuo* to afford an oil. This residue was then reacted with triethylamine (6.4 mL, 46.47 mmol) and diphenylphosphorylazide (6.7 mL, 30.98 mmol) in anhydrous toluene (20 mL) at 0 °C while stirring under an argon atmosphere. The reaction mixture was warmed to 25 °C over 4 h, then washed with water (3 × 50 mL), brine (1 × 30 mL), dried over Na_2_SO_4_, filtered and concentrated *in vacuo*. The residual azide was dissolved in anhydrous *tert*-butanol (100 mL) while stirring and heated to reflux for 6 h. After completion, the reaction was cooled to 25 °C and concentrated *in vacuo* to afford a brown oil. The oil was diluted with EtOAc (50 mL) and washed with saturated sodium bicarbonate (1 × 50 mL), brine (1 × 30 mL), dried over Na_2_SO_4_, filtered and concentrated *in vacuo*. The crude carbamate was purified by flash chromatography in PE/EtOAc (30 : 1) to yield the pure product 12a (first elution) and 12b (second elution). 12a: white solid, yield 27%. ^1^H NMR (400 MHz, CDCl_3_) *δ* 7.18 (d, *J* = 4.0 Hz, 1H), 7.11–7.10 (m, 2H), 6.65–6.63 (m, 1H), 4.78 (s, 1H), 3.12–2.99 (m, 2H), 2.75 (s, 1H), 2.18–2.02 (m, 2H), 1.44–1.33 (m, 9H), 1.26–0.90 (m, 2H). ^13^C NMR (100 MHz, CDCl_3_) *δ* 156.4, 146.2, 143.7, 126.4, 126.1, 124.3, 118.5, 79.5, 36.2, 32.9, 30.8, 28.9, 28.3, 21.4. 12b: white solid; yield 19%. ^1^H NMR (400 MHz, CDCl_3_) *δ* 7.24–7.20 (m, 1H), 7.15–7.09 (m, 2H), 6.81 (s, 1H), 4.86–4.67 (m, 1H), 3.16–2.92 (m, 2H), 2.76 (s, 1H), 2.28–1.90 (m, 2H), 1.31–1.12 (m, 11H). ^13^C NMR (101 MHz, CDCl_3_) *δ* 156.3, 145.7, 142.4, 126.2, 125.8, 124.4, 121.2, 79.2, 36.2, 35.1, 33.4, 30.8, 28.2, 19.6.

#### (2*R*)-2′,3′-Dihydrospiro[cyclopropane-1,1′-inden]-2-amine hydrochloride (8a)

A solution of 12a (300 mg, 1.16 mmol) and 4 M HCl in EtOAc (5 mL) was stirred at room temperature overnight. The precipitated solid was filtered, washed with diethyl ether and dried to afford 8a as hydrochloride salt. Yellow solid; yield 98%. ^1^H NMR (400 MHz, CD_3_OD) *δ* 7.23–7.21 (m, 1H), 7.18–7.12 (m, 2H), 6.77–6.75 (m, 1H), 3.14 (t, *J* = 7.6 Hz, 2H), 2.88 (dd, *J* = 8.0, 4.8 Hz, 1H), 2.34–2.20 (m, 2H), 1.44–1.27 (m, 2H). ^13^C NMR (101 MHz, CD_3_OD) *δ* 145.4, 144.8, 128.2, 128.0, 125.5, 120.0, 35.3, 31.9, 31.5, 29.4, 19.9. HRMS (ESI): calcd for C_11_H_14_N [M + H]^+^, 160.1121; found 160.1114.

#### (2*S*)-2′,3′-Dihydrospiro[cyclopropane-1,1′-inden]-2-amine hydrochloride (8b)

This compound was synthesized from 12b according to the methodology described for 12a. Yellow solid; yield 94%. ^1^H NMR (400 MHz, CD_3_OD) *δ* 7.32–7.31 (m, 1H), 7.26–7.21 (m, 2H), 7.08–7.06 (m, 1H), 3.18–2.94 (m, 2H), 2.88 (dd, *J* = 7.6, 4.8 Hz, 1H), 2.38–1.94 (m, 2H), 1.52–1.41 (m, 2H). ^13^C NMR (101 MHz, CD_3_OD) *δ* 147.5, 140.4, 128.7, 127.7, 126.3, 121.9, 36.6, 35.0, 33.1, 31.5, 15.7. HRMS (ESI): calcd for C_11_H_14_N [M + H]^+^, 160.1121; found 160.1124.

#### (2*R*)-*N*-Benzyl-2′,3′-dihydrospiro[cyclopropane-1,1′-inden]-2-amine (13a)

Triethylamine (39 mg, 0.39 mmol) was added to a solution of 8a (50 mg, 0.26 mmol) in MeOH (2 mL) at rt to produce the free amine. Then benzaldehyde (28 mg, 0.26 mmol) was added and the mixture was stirred at rt for 0.5 h. Molecular sieves type 4 Å (300 mg) were added and the mixture was stirred for another 0.25 h. Sodium borohydride (15 mg, 0.39 mmol) was then added in a single portion. After overnight stirring, the mixture was then filtered and the solvent removed *in vacuo* to give the crude product which was partitioned between saturated NaHCO_3_ (20 mL) and EtOAc (20 mL), followed by extraction with EtOAc (3 × 30 mL). The organic phase was combined, dried over Na_2_SO_4_, filtered, and concentrated *in vacuo*. The crude product was purified by flash chromatography in PE/EtOAc (15 : 1) to yield the pure product 13a. Yellow oil; yield 42%. ^1^H NMR (400 MHz, CDCl_3_) *δ* 7.32–7.28 (m, 4H), 7.26–7.23 (m, 1H), 7.20–7.17 (m, 1H), 7.12–7.08 (m, 2H), 6.64–6.60 (m, 1H), 3.86–3.78 (m, 2H), 3.08–2.92 (m, 2H), 2.42–2.34 (m, 2H), 2.17–2.09 (m, 1H), 1.90 (s, 1H), 1.18–0.85 (m, 2H)·^13^C NMR (101 MHz, CDCl_3_) *δ* 147.8, 143.8, 140.4, 128.4, 128.2, 127.0, 126.3, 125.7, 124.2, 118.4, 53.9, 44.8, 33.6, 30.9, 28.4, 22.0. HRMS (ESI): calcd for C_18_H_20_N [M + H]^+^, 250.1590; found 250.1594.

#### (2*S*)-*N*-Benzyl-2′,3′-dihydrospiro[cyclopropane-1,1′-inden]-2-amine (13b)

This compound was obtained from 8b and benzaldehyde according to the methodology described for 13a. Yellow oil; yield 39%. ^1^H NMR (400 MHz, CDCl_3_) *δ* 7.27–7.23 (m, 2H), 7.21–7.17 (m, 2H), 7.15–7.13 (m, 5H), 3.58–3.39 (m, 2H), 3.07–2.91 (m, 2H), 2.44 (dd, *J* = 7.0, 4.6 Hz, 1H), 2.23–1.82 (m, 2H), 1.08–1.01 (m, 2H). ^13^C NMR (101 MHz, CDCl_3_) *δ* 145.2, 143.9, 140.5, 128.3, 128.2, 126.8, 125.7, 125.6, 124.0, 122.3, 53.5, 44.8, 35.5, 33.8, 30.8, 20.4. HRMS (ESI): calcd for C_18_H_20_N [M + H]^+^, 250.1590; found 250.1601.

#### (2*R*)-*N*-(2-Methoxybenzyl)-2′,3′-dihydrospiro[cyclopropane-1,1′-inden]-2-amine (14a)

This compound was obtained from 8a and 2-methoxybenzaldehyde according to the methodology described for 13a. Yellow oil; yield 65%. ^1^H NMR (400 MHz, CDCl_3_) *δ* 7.23–7.17 (m, 3H), 7.11–7.05 (m, 2H), 6.89–6.85 (m, 1H), 6.82 (d, *J* = 8.0 Hz, 1H), 6.60–6.56 (m, 1H), 3.88–3.74 (m, 5H), 3.07–2.93 (m, 2H), 2.43–2.36 (m, 1H), 2.28 (dd, *J* = 7.4, 4.6 Hz, 1H), 2.15–2.07 (m, 1H), 2.03 (s, 1H), 1.15–0.85 (m, 2H). ^13^C NMR (101 MHz, CDCl_3_) *δ* 157.6, 148.0, 143.8, 129.9, 128.4, 128.2, 126.2, 125.6, 124.1, 120.3, 118.4, 110.2, 55.1, 49.2, 44.7, 33.5, 30.9, 28.3, 22.0. HRMS (ESI): calcd for C_19_H_22_NO [M + H]^+^, 280.1696; found 280.1676.

#### (2*S*)-*N*-(2-Methoxybenzyl)-2′,3′-dihydrospiro[cyclopropane-1,1′-inden]-2-amine (14b)

This compound was obtained from 8b and 2-methoxybenzaldehyde according to the methodology described for 13a. Yellow oil; yield 62%. ^1^H NMR (400 MHz, CDCl_3_) *δ* 7.22–7.16 (m, 2H), 7.13–7.10 (m, 3H), 7.04–7.01 (m, 1H), 6.86–6.82 (m, 1H), 6.78 (d, *J* = 8.0 Hz, 1H), 3.76 (s, 3H), 3.61–3.44 (m, 2H), 3.06–2.89 (m, 2H), 2.39 (dd, *J* = 6.8, 5.2 Hz, 1H), 2.18–1.81 (m, 3H), 1.04–1.00 (m, 2H). ^13^C NMR (101 MHz, CDCl_3_) *δ* 157.6, 145.1, 144.2, 130.0, 128.6, 128.0, 125.5, 125.5, 123.9, 122.4, 120.2, 110.0, 55.1, 48.7, 44.7, 35.7, 33.7, 30.8, 20.7. HRMS (ESI): calcd for C_19_H_22_NO [M + H]^+^, 280.1696; found 280.1707.

#### (2*R*)-*N*-(2-Fluorobenzyl)-2′,3′-dihydrospiro[cyclopropane-1,1′-inden]-2-amine (15a)

This compound was obtained from 8a and 2-fluorobenzaldehyde according to the methodology described for 13a. Yellow oil; yield 52%. ^1^H NMR (400 MHz, CDCl_3_) *δ* 7.30–7.26 (m, 1H), 7.24–7.16 (m, 2H), 7.12–6.98 (m, 4H), 6.62–6.58 (m, 1H), 3.92–3.81 (m, 2H), 3.07–2.92 (m, 2H), 2.41–2.30 (m, 2H), 2.15–2.10 (m, 1H), 1.80 (s, 1H), 1.17–0.85 (m, 2H). ^13^C NMR (101 MHz, CDCl_3_) *δ* 161.3 (d, *J*_C–F_ = 246.5 Hz), 147.8, 143.9, 130.6 (d, *J*_C–F_ = 4.9 Hz), 128.8 (d, *J*_C–F_ = 8.2 Hz), 127.4 (d, *J*_C–F_ = 15.3 Hz), 126.4, 125.9, 124.3, 124.1 (d, *J*_C–F_ = 3.5 Hz), 118.5, 115.4 (d, *J*_C–F_ = 21.9 Hz), 47.5 (d, *J*_C–F_ = 2.9 Hz), 44.7, 33.7, 31.0, 28.4, 22.2. HRMS (ESI): calcd for C_18_H_19_FN [M + H]^+^, 268.1496; found 268.1499.

#### (2*S*)-*N*-(2-Fluorobenzyl)-2′,3′-dihydrospiro[cyclopropane-1,1′-inden]-2-amine (15b)

This compound was obtained from 8b and 2-fluorobenzaldehyde according to the methodology described for 13a. Yellow oil; yield 60%. ^1^H NMR (400 MHz, CDCl_3_) *δ* 7.22–7.09 (m, 6H), 7.03–6.93 (m, 2H), 3.66–3.48 (m, 2H), 3.06–2.90 (m, 2H), 2.41 (dd, *J* = 7.0, 4.6 Hz, 1H), 2.20–1.81 (m, 2H), 1.65 (s, 1H), 1.06–1.00 (m, 2H). ^13^C NMR (101 MHz, CDCl_3_) *δ* 161.3 (d, *J*_C–F_ = 246.4 Hz), 145.2, 144.0, 130.8 (d, *J*_C–F_ = 5.0 Hz), 128.6 (d, *J*_C–F_ = 8.1 Hz), 127.4 (d, *J*_C–F_ = 15.4 Hz), 125.8, 125.7, 124.1, 123.9 (d, *J*_C–F_ = 3.5 Hz), 122.5, 115.2 (d, *J*_C–F_ = 22.0 Hz), 46.8 (d, *J*_C–F_ = 2.8 Hz), 44.6, 35.6, 33.9, 30.9, 20.8. HRMS (ESI): calcd for C_18_H_19_FN [M + H]^+^, 268.1496; found 268.1502.

#### (2*R*)-*N*-(5-Bromo-2-methoxybenzyl)-2′,3′-dihydrospiro[cyclopropane-1,1′-inden]-2-amine (16a)

This compound was obtained from 8a and 5-bromo-2-methoxybenzaldehyde according to the methodology described for 13a. Yellow oil; yield 57%. ^1^H NMR (400 MHz, CDCl_3_) *δ* 7.34–7.29 (m, 2H), 7.20–7.18 (m, 1H), 7.12–7.07 (m, 2H), 6.69 (d, *J* = 8.8 Hz, 1H), 6.62–6.58 (m, 1H), 3.86–3.67 (m, 5H), 3.08–2.93 (m, 2H), 2.40–2.33 (m, 1H), 2.27 (dd, *J* = 7.2, 4.8 Hz, 1H), 2.15–2.08 (m, 1H), 2.03–2.00 (m, 1H), 1.17–0.85 (m, 2H). ^13^C NMR (101 MHz, CDCl_3_) *δ* 156.7, 147.8, 143.8, 132.4, 130.8, 130.7, 126.3, 125.7, 124.2, 118.5, 112.7, 111.9, 55.4, 48.6, 44.6, 33.6, 30.8, 28.3, 22.0. HRMS (ESI): calcd for C_19_H_21_BrNO [M + H]^+^, 358.0801; found 358.0768.

#### (2*S*)-*N*-(5-Bromo-2-methoxybenzyl)-2′,3′-dihydrospiro[cyclopropane-1,1′-inden]-2-amine (16b)

This compound was obtained from 8b and 5-bromo-2-methoxybenzaldehyde according to the methodology described for 13a. Yellow oil; yield 67%. ^1^H NMR (400 MHz, CDCl_3_) *δ* 7.26–7.23 (m, 1H), 7.19–7.16 (m, 1H), 7.14–7.07 (m, 4H), 6.61 (d, *J* = 8.4 Hz, 1H), 3.72 (s, 3H), 3.60–3.36 (m, 2H), 3.05–2.89 (m, 2H), 2.36 (dd, *J* = 6.8, 4.8 Hz, 1H), 2.20–1.80 (m, 3H), 1.05–0.98 (m, 2H). ^13^C NMR (101 MHz, CDCl_3_) *δ* 156.5, 145.1, 143.9, 132.6, 130.8, 130.5, 125.6, 125.5, 123.9, 122.3, 112.4, 111.7, 55.3, 48.2, 44.7, 35.5, 33.8, 30.8, 20.5. HRMS (ESI): calcd for C_19_H_21_BrNO [M + H]^+^, 358.0801; found 358.0795.

#### (2*R*)-*N*-(5-Fluoro-2-methoxybenzyl)-2′,3′-dihydrospiro[cyclopropane-1,1′-inden]-2-amine (17a)

This compound was obtained from 8a and 5-fluoro-2-methoxybenzaldehyde according to the methodology described for 13a. Yellow oil; yield 65%. ^1^H NMR (400 MHz, CDCl_3_) *δ* 7.21–7.17 (m, 1H), 7.11–7.07 (m, 2H), 6.97 (dd, *J* = 8.6, 3.0 Hz, 1H), 6.91–6.86 (m, 1H), 6.73 (dd, *J* = 8.8, 4.4 Hz, 1H), 6.61–6.57 (m, 1H), 3.88–3.68 (m, 5H), 3.08–2.94 (m, 2H), 2.42–2.35 (m, 1H), 2.28 (dd, *J* = 7.4, 4.6 Hz, 1H), 2.15–2.08 (m, 1H), 1.93 (s, 1H), 1.16–0.85 (m, 2H). ^13^C NMR (101 MHz, CDCl_3_) *δ* 156.9 (d, *J*_C–F_ = 239.3 Hz), 153.6 (d, *J*_C–F_ = 1.9 Hz), 147.9, 143.8, 130.3 (d, *J*_C–F_ = 6.7 Hz), 126.3, 125.7, 124.2, 118.5, 116.5 (d, *J*_C–F_ = 23.2 Hz), 113.6 (d, *J*_C–F_ = 22.8 Hz), 110.9 (d, *J*_C–F_ = 8.2 Hz), 55.6, 48.8 (d, *J*_C–F_ = 0.7 Hz), 44.6, 33.6, 30.9, 28.3, 22.0. HRMS (ESI): calcd for C_19_H_21_FNO [M + H]^+^, 298.1602; found 298.1593.

#### (2*S*)-*N*-(5-Fluoro-2-methoxybenzyl)-2′,3′-dihydrospiro[cyclopropane-1,1′-inden]-2-amine (17b)

This compound was obtained from 8b and 5-fluoro-2-methoxybenzaldehyde according to the methodology described for 13a. Yellow oil; yield 70%. ^1^H NMR (400 MHz, CDCl_3_) *δ* 7.23–7.21 (m, 1H), 7.17–7.14 (m, 3H), 6.90–6.80 (m, 2H), 6.71 (dd, *J* = 8.8, 4.4 Hz, 1H), 3.76 (s, 3H), 3.64–3.42 (m, 2H), 3.09–2.93 (m, 2H), 2.42 (dd, *J* = 6.8, 4.8 Hz, 1H), 2.23–1.84 (m, 3H), 1.09–1.03 (m, 2H). ^13^C NMR (101 MHz, CDCl_3_) *δ* 156.8 (d, *J*_C–F_ = 239.0 Hz), 153.6 (d, *J*_C–F_ = 2.0 Hz), 145.1, 144.0, 130.4 (d, *J*_C–F_ = 6.7 Hz), 125.7, 125.6, 124.0, 122.4, 116.6 (d, *J*_C–F_ = 23.1 Hz), 113.4 (d, *J*_C–F_ = 22.9 Hz), 110.7 (d, *J*_C–F_ = 8.3 Hz), 55.7, 48.3 (d, *J*_C–F_ = 0.8 Hz). 44.7, 35.6, 33.8, 30.8, 20.7. HRMS (ESI): calcd for C_19_H_21_FNO [M + H]^+^, 298.1602; found 298.1584.

#### (2*R*)-*N*-(4-Fluoro-2-methoxybenzyl)-2′,3′-dihydrospiro[cyclopropane-1,1′-inden]-2-amine (18a)

This compound was obtained from 8a and 4-fluoro-2-methoxybenzaldehyde according to the methodology described for 13a. Yellow oil; yield 71%. ^1^H NMR (400 MHz, CDCl_3_) *δ* 7.19–7.17 (m, 1H), 7.14–7.07 (m, 3H), 6.59–6.56 (m, 2H), 6.54–6.53 (m, 1H), 3.83–3.70 (m, 5H), 3.07–2.92 (m, 2H), 2.40–2.33 (m, 1H), 2.25 (dd, *J* = 7.6, 4.8 Hz, 1H), 2.14–2.07 (m, 1H), 1.97 (s, 1H), 1.15–0.84 (m, 2H). ^13^C NMR (101 MHz, CDCl_3_) *δ* 161.9 (d, *J*_C–F_ = 245.2 Hz), 157.6 (d, *J*_C–F_ = 9.8 Hz), 146.9, 142.7, 129.6 (d, *J*_C–F_ = 9.9 Hz), 125.3, 124.6, 123.2, 123.1 (d, *J*_C–F_ = 3.3 Hz), 117.4, 105.3 (d, *J*_C–F_ = 21.0 Hz), 97.7 (d, *J*_C–F_ = 25.9 Hz). 54.3, 47.6, 43.6, 32.5, 29.8, 27.2, 20.9. HRMS (ESI): calcd for C_19_H_21_FNO [M + H]^+^, 298.1602; found 298.1606.

#### (2*S*)-*N*-(4-Fluoro-2-methoxybenzyl)-2′,3′-dihydrospiro[cyclopropane-1,1′-inden]-2-amine (18b)

This compound was obtained from 8b and 4-fluoro-2-methoxybenzaldehyde according to the methodology described for 13a. Yellow oil; yield 66%. ^1^H NMR (400 MHz, CDCl_3_) *δ* 7.19–7.17 (m, 1H), 7.14–7.07 (m, 3H), 6.27 (t, *J* = 7.6 Hz, 1H), 6.54–6.47 (m, 2H), 3.72 (s, 3H), 3.59–3.36 (m, 2H), 3.03–2.88 (m, 2H), 2.37 (dd, *J* = 6.8, 4.8 Hz, 1H), 2.19–2.12 (m, 1H), 1.92 (s, 1H), 1.85–1.79 (m, 1H), 1.05–1.00 (m, 2H). ^13^C NMR (101 MHz, CDCl_3_) *δ* 161.8 (d, *J*_C–F_ = 244.9 Hz), 157.5 (d, *J*_C–F_ = 9.7 Hz), 144.1, 143.0, 129.6 (d, *J*_C–F_ = 9.8 Hz), 124.6, 124.5, 123.0 (d, *J*_C–F_ = 2.8 Hz), 122.9, 121.2, 105.1 (d, *J*_C–F_ = 20.9 Hz), 97.6 (d, *J*_C–F_ = 26.0 Hz), 54.3, 47.1, 43.7, 34.6, 32.7, 29.8, 19.4. HRMS (ESI): calcd for C_19_H_21_FNO [M + H]^+^, 298.1602; found 298.1599.

#### (2*R*)-*N*-(3,4-Fimethoxybenzyl)-2′,3′-dihydrospiro[cyclopropane-1,1′-inden]-2-amine (19a)

This compound was obtained from 8a and 3,4-dimethoxybenzaldehyde according to the methodology described for 13a. Yellow oil; yield 46%. ^1^H NMR (400 MHz, CDCl_3_) *δ* 7.19–7.16 (m, 1H), 7.12–7.07 (m, 2H), 6.84–6.78 (m, 3H), 6.63–6.59 (m, 1H), 3.85 (s, 3H), 3.79–3.73 (m, 5H), 3.07–2.90 (m, 2H), 2.37–2.30 (m, 2H), 2.17–2.08 (m, 1H), 2.04–1.96 (m, 1H), 1.18–0.85 (m, 2H). ^13^C NMR (101 MHz, CDCl_3_) *δ* 148.8, 148.0, 147.8, 143.7, 133.0, 126.3, 125.7, 124.2, 120.3, 118.4, 111.7, 111.1, 56.0, 55.7, 53.8, 44.9, 33.5, 30.8, 28.4, 21.9. HRMS (ESI): calcd for C_20_H_24_NO_2_ [M + H]^+^, 310.1807; found 310.1787.

#### (2*S*)-*N*-(3,4-Fimethoxybenzyl)-2′,3′-dihydrospiro[cyclopropane-1,1′-inden]-2-amine (19b)

This compound was obtained from 8b and 3,4-dimethoxybenzaldehyde according to the methodology described for 13a. Yellow oil; yield 51%. ^1^H NMR (400 MHz, CDCl_3_) *δ* 7.23–7.21 (m, 1H), 7.16–7.13 (m, 3H), 6.75 (d, *J* = 8.4 Hz, 1H), 6.68–6.65 (m, 2H), 3.85–3.84 (m, 6H), 3.54–3.27 (m, 2H), 3.09–2.93 (m, 2H), 2.46 (dd, *J* = 6.8, 4.8 Hz, 1H), 2.26–2.18 (m, 1H), 1.89–1.83 (m, 2H), 1.10–1.04 (m, 2H). ^13^C NMR (101 MHz, CDCl_3_) *δ* 148.8, 147.9, 145.2, 143.8, 133.0, 125.7, 125.4, 124.0, 122.2, 120.3, 111.7, 110.8, 55.9, 55.8, 53.3, 44.8, 35.6, 33.8, 30.8, 20.0. HRMS (ESI): calcd for C_20_H_24_NO_2_ [M + H]^+^, 310.1807; found 310.1787.

#### (2*R*)-*N*-(2-Chloro-3,4-dimethoxybenzyl)-2′,3′-dihydrospiro[cyclopropane-1,1′-inden]-2-amine (20a)

This compound was obtained from 8a and 2-chloro-3,4-dimethoxybenzaldehyde according to the methodology described for 13a. Yellow oil; yield 64%. ^1^H NMR (400 MHz, CDCl_3_) *δ* 7.21–7.19 (m, 1H), 7.14–7.09 (m, 2H), 7.02 (d, *J* = 8.4 Hz, 1H), 6.76 (d, *J* = 8.4 Hz, 1H), 6.64–6.60 (m, 1H), 3.93–3.83 (m, 8H), 3.10–2.95 (m, 2H), 2.44–2.37 (m, 1H), 2.31 (dd, *J* = 7.2, 4.8 Hz, 1H), 2.18–2.10 (m, 1H), 1.91 (s, 1H), 1.19–0.88 (m, 2H). ^13^C NMR (101 MHz, CDCl_3_) *δ* 151.8, 146.7, 144.6, 142.8, 129.7, 127.2, 125.3, 124.7, 124.0, 123.2, 117.4, 109.2, 60.0, 55.1, 50.3, 43.4, 32.6, 29.8, 27.3, 21.0. HRMS (ESI): calcd for C_20_H_23_ClNO_2_ [M + H]^+^, 344.1412; found 344.1390.

#### (2*S*)-*N*-(2-Chloro-3,4-dimethoxybenzyl)-2′,3′-dihydrospiro[cyclopropane-1,1′-inden]-2-amine (20b)

This compound was obtained from 8b and 2-chloro-3,4-dimethoxybenzaldehyde according to the methodology described for 13a. Yellow oil; yield 68%. ^1^H NMR (400 MHz, CDCl_3_) *δ* 7.19–7.18 (m, 1H), 7.13–7.10 (m, 3H), 6.81 (d, *J* = 8.4 Hz, 1H), 6.71 (d, *J* = 8.4 Hz, 1H), 3.83 (s, 6H), 3.65–3.48 (m, 2H), 3.08–2.91 (m, 2H), 2.39 (dd, *J* = 6.8, 4.8 Hz, 1H), 2.20–1.82 (m, 2H), 1.76 (s, 1H), 1.06–1.00 (m, 2H). ^13^C NMR (101 MHz, CDCl_3_) *δ* 152.6, 145.4, 145.1, 144.0, 130.9, 128.3, 125.7, 125.6, 125.2, 123.9, 122.5, 110.2, 60.6, 56.1, 50.8, 44.5, 35.6, 33.9, 30.9, 20.8. HRMS (ESI): calcd for C_20_H_23_ClNO_2_ [M + H]^+^, 344.1412; found 344.1402.

#### (2*R*)-*N*-(Pyridin-3-ylmethyl)-2′,3′-dihydrospiro[cyclopropane-1,1′-inden]-2-amine (21a)

This compound was obtained from 8a and nicotinaldehyde according to the methodology described for 13a. Yellow oil; yield 40%. ^1^H NMR (400 MHz, CD_3_OD) *δ* 8.47 (d, *J* = 2.0 Hz, 1H), 8.40 (dd, *J* = 5.0, 1.4 Hz, 1H), 7.80–7.78 (m, 1H), 7.35 (dd, *J* = 8.0, 4.8 Hz, 1H), 7.14–7.11 (m, 1H), 7.07–7.02 (m, 2H), 6.62–6.58 (m, 1H), 3.83 (s, 2H), 3.02–2.84 (m, 2H), 2.32–2.25 (m, 2H), 2.12–2.04 (m, 1H), 1.17–0.81 (m, 2H). ^13^C NMR (101 MHz, CD_3_OD) *δ* 150.3, 148.6, 148.5, 144.6, 138.6, 137.5, 127.4, 126.8, 125.1, 125.1, 119.4, 51.8, 45.9, 34.2, 31.6, 29.5, 22.0. HRMS (ESI): calcd for C_17_H_19_N_2_ [M + H]^+^, 251.1543; found 251.1521.

#### (2*S*)-*N*-(Pyridin-3-ylmethyl)-2′,3′-dihydrospiro[cyclopropane-1,1′-inden]-2-amine (21b)

This compound was synthesized from 8b according to the methodology described for 13a. Yellow oil; yield 60%. ^1^H NMR (400 MHz, CD_3_OD) *δ* 8.30 (dd, *J* = 4.8, 1.6 Hz, 1H), 8.20 (d, *J* = 2.0 Hz, 1H), 7.51–7.48 (m, 1H), 7.23–7.20 (m, 1H), 7.15–7.05 (m, 4H), 3.57–3.24 (m, 2H), 2.97–2.83 (m, 2H), 2.41 (dd, *J* = 7.2, 4.8 Hz, 1H), 2.20–1.76 (m, 2H), 1.07–1.02 (m, 2H). ^13^C NMR (101 MHz, CD_3_OD) *δ* 150.0, 148.4, 146.3, 144.5, 138.3, 137.4, 126.9, 126.6, 125.0, 124.8, 123.0, 51.4, 46.0, 36.7, 34.8, 31.5, 19.6. HRMS (ESI): calcd for C_17_H_19_N_2_ [M + H]^+^, 251.1543; found 251.1537.

#### (2*R*)-*N*-((2-Methoxypyridin-3-yl)methyl)-2′,3′-dihydrospiro[cyclopropane-1,1′-inden]-2-amine (22a)

This compound was obtained from 8a and 2-methoxynicotinaldehyde according to the methodology described for 13a. Yellow oil; yield 61%. ^1^H NMR (400 MHz, CDCl_3_) *δ* 8.07–8.05 (m, 1H), 7.47 (d, *J* = 7.2 Hz, 1H), 7.20–7.18 (m, 1H), 7.12–7.07 (m, 2H), 6.81 (dd, *J* = 7.0, 5.4 Hz, 1H), 6.59 (t, *J* = 4.2 Hz, 1H), 3.91 (s, 3H), 3.84–3.72 (m, 2H), 3.09–2.93 (m, 2H), 2.41–2.34 (m, 1H), 2.29–2.26 (m, 1H), 2.16–2.09 (m, 3H), 1.17–1.14 (m, 1H). ^13^C NMR (101 MHz, CDCl_3_) *δ* 162.1, 147.8, 145.3, 143.7, 137.8, 126.3, 125.8, 124.2, 122.6, 118.4, 116.7, 53.3, 48.7, 44.5, 33.5, 30.8, 28.3, 22.0. HRMS (ESI): calcd for C_18_H_21_N_2_O [M + H]^+^, 281.1648; found 281.1654.

#### (2*S*)-*N*-((2-Methoxypyridin-3-yl)methyl)-2′,3′-dihydrospiro[cyclopropane-1,1′-inden]-2-amine (22b)

This compound was obtained from 8b and 2-methoxynicotinaldehyde according to the methodology described for 13a. Yellow oil; yield 50%. ^1^H NMR (400 MHz, CD_3_OD) *δ* 7.91 (d, *J* = 4.4 Hz, 1H), 7.36–7.32 (m, 1H), 7.13–7.06 (m, 3H), 6.99–6.97 (m, 1H), 6.81–6.77 (m, 1H), 3.82 (s, 3H), 3.58–3.29 (m, 2H), 2.95–2.80 (m, 2H), 2.38 (dd, *J* = 7.0, 5.0 Hz, 1H), 2.20–1.75 (m, 2H), 1.06–0.96 (m, 2H). ^13^C NMR (101 MHz, CD_3_OD) *δ* 163.3, 146.4, 146.2, 144.4, 139.5, 127.0, 126.7, 125.2, 123.6, 122.6, 117.9, 53.9, 46.0, 36.9, 34.7, 31.6, 19.4, 19.3. HRMS (ESI): calcd for C_18_H_21_N_2_O [M + H]^+^, 281.1648; found 281.1665.

#### (2*R*)-*N*-((2-Fluoropyridin-3-yl)methyl)-2′,3′-dihydrospiro[cyclopropane-1,1′-inden]-2-amine (23a)

This compound was obtained from 8a and 2-fluoronicotinaldehyde according to the methodology described for 13a. Yellow oil; yield 22%. ^1^H NMR (400 MHz, CDCl_3_) *δ* 8.10 (d, *J* = 4.8 Hz, 1H), 7.77–7.72 (m, 1H), 7.20–7.18 (m, 1H), 7.14–7.07 (m, 3H), 6.62–6.58 (m, 1H), 3.93–3.83 (m, 2H), 3.09–2.92 (m, 2H), 2.39–2.30 (m, 2H), 2.16–2.08 (m, 1H), 1.90 (s, 1H), 1.19–0.85 (m, 2H). ^13^C NMR (101 MHz, CDCl_3_) *δ* 161.9 (d, *J*_C–F_ = 239.9 Hz), 147.4, 146.0 (d, *J*_C–F_ = 14.9 Hz), 143.7, 140.6 (d, *J*_C–F_ = 5.8 Hz), 126.4, 125.9, 124.3, 122.2 (d, *J*_C–F_ = 30.0 Hz), 121.4 (d, *J*_C–F_ = 4.2 Hz). 118.4, 46.9, 44.4, 33.6, 30.8, 28.3, 22.0. HRMS (ESI): calcd for C_17_H_18_FN_2_ [M + H]^+^, 269.1449; found 269.1471.

#### (2*S*)-*N*-((2-Fluoropyridin-3-yl)methyl)-2′,3′-dihydrospiro[cyclopropane-1,1′-inden]-2-amine (23b)

This compound was obtained from 8b and 2-fluoronicotinaldehyde according to the methodology described for 13a. Yellow oil; yield 30%. ^1^H NMR (400 MHz, CDCl_3_) *δ* 8.03 (d, *J* = 4.8 Hz, 1H), 7.54–7.49 (m, 1H), 7.19–7.17 (m, 1H), 7.14–7.07 (m, 3H), 7.06–7.02 (m, 1H), 3.69–3.47 (m, 2H), 3.03–2.89 (m, 2H), 2.41 (dd, *J* = 6.8, 4.8 Hz, 1H), 2.22–2.14 (m, 1H), 1.87–1.81 (m, 1H), 1.76 (s, 1H), 1.08–1.00 (m, 2H). ^13^C NMR (101 MHz, CDCl_3_) *δ* 161.8 (d, *J*_C–F_ = 239.7 Hz), 145.8 (d, *J*_C–F_ = 14.8 Hz), 145.1, 143.5, 140.8 (d, *J*_C–F_ = 5.9 Hz), 125.8, 125.5, 124.0, 122.4, 122.1 (d, *J*_C–F_ = 30.0 Hz), 121.3 (d, *J*_C–F_ = 4.2 Hz), 46.4, 44.5, 35.4, 30.7, 29.7, 20.5. HRMS (ESI): calcd for C_17_H_18_FN_2_ [M + H]^+^, 269.1449; found 269.1448.

#### (2*R*)-*N*-((3-Fluoropyridin-2-yl)methyl)-2′,3′-dihydrospiro[cyclopropane-1,1′-inden]-2-amine (24a)

This compound was obtained from 8a and 3-fluoropicolinaldehyde according to the methodology described for 13a. Yellow oil; yield 47%. ^1^H NMR (400 MHz, CDCl_3_) *δ* 8.39–8.37 (m, 1H), 7.37–7.32 (m, 1H), 7.22–7.18 (m, 2H), 7.13–7.08 (m, 2H), 6.64–6.60 (m, 1H), 4.09–4.01 (m, 2H), 3.07–3.03 (m, 2H), 2.47–2.36 (m, 3H), 2.20–2.12 (m, 1H), 1.21–0.92 (m, 2H). ^13^C NMR (101 MHz, CDCl_3_) *δ* 157.5 (d, *J*_C–F_ = 257.6 Hz), 147.8 (d, *J*_C–F_ = 15.8 Hz), 147.7, 144.9 (d, *J*_C–F_ = 5.3 Hz), 143.9, 126.3, 125.8, 124.2, 123.2 (d, *J*_C–F_ = 3.7 Hz), 122.6 (d, *J*_C–F_ = 19.1 Hz), 118.5, 48.4, 44.7, 33.5, 30.9, 28.3, 22.1. HRMS (ESI): calcd for C_17_H_18_FN_2_ [M + H]^+^, 269.1449; found 269.1431.

#### (2*S*)-*N*-((3-Fluoropyridin-2-yl)methyl)-2′,3′-dihydrospiro[cyclopropane-1,1′-inden]-2-amine (24b)

This compound was obtained from 8b and 3-fluoropicolinaldehyde according to the methodology described for 13a. Yellow oil; yield 59%. ^1^H NMR (400 MHz, CDCl_3_) *δ* 8.30 (d, *J* = 4.8 Hz, 1H), 7.25–7.21 (m, 1H), 7.16–7.07 (m, 5H), 3.81–3.71 (m, 2H), 3.04–2.87 (m, 2H), 2.45 (dd, *J* = 6.8, 4.8 Hz, 1H), 2.22 (s, 1H), 2.17–1.82 (m, 2H), 1.07–1.00 (m, 2H). ^13^C NMR (101 MHz, CDCl_3_) *δ* 157.6 (d, *J*_C–F_ = 257.6 Hz), 148.1 (d, *J*_C–F_ = 15.7 Hz), 145.0, 144.8 (d, *J*_C–F_ = 5.4 Hz), 143.9, 125.6, 125.5, 123.9, 123.1 (d, *J*_C–F_ = 3.6 Hz), 122.5, 122.4 (d, *J*_C–F_ = 19.2 Hz), 48.3, 44.8, 35.6, 33.7, 30.7, 20.8. HRMS (ESI): calcd for C_17_H_18_FN_2_ [M + H]^+^, 269.1449; found 269.1447.

#### 2-(((2*R*)-2′,3′-Dihydrospiro[cyclopropane-1,1′-inden]-2-yl)amino)-1-morpholinoethan-1-one hydrochloride (25a)

To a stirred suspension of NaH (28 mg, 50% in mineral oil, 0.59 mmol) in anhydrous DMF (2 mL) at 0 °C was added a solution of 12a (100 mg, 0.39 mmol). After an additional 0.5 h stirring, 2-chloro-1-morpholinoethan-1-one (77 mg, 0.47 mmol) was added at 0 °C and the mixture was stirred for another 1 h, slowly warming to rt. The reaction mixture was poured into ice-water and extracted with EtOAc (3 × 30 mL). The combined organic layers were washed with water, brine, dried over anhydrous Na_2_SO_4_, filtered and evaporated. The crude residue was purified by flash chromatography using CH_2_Cl_2_ : CH_3_OH (60 : 1) to afford a yellow solid, which was then immediately treated with 4 M HCl in ethyl acetate (5 mL) and stirred at rt overnight. The precipitated solid was filtered, washed with diethyl ether and dried to afford 25a as a hydrochloride salt. Yellow solid; yield 48%. ^1^H NMR (400 MHz, CD_3_OD) *δ* 7.24–7.16 (m, 3H), 6.77 (d, *J* = 4.0 Hz, 1H), 4.31 (s, 2H), 3.70–3.60 (m, 6H), 3.48 (s, 2H), 3.17–3.06 (m, 3H), 2.40–2.33 (m, 2H), 1.45 (s, 2H). ^13^C NMR (101 MHz, CD_3_OD) *δ* 164.9, 145.1, 144.8, 128.4, 128.0, 125.5, 120.1, 67.5, 67.4, 50.0, 46.3, 43.5, 42.9, 33.0, 31.7, 29.5, 19.5. HRMS (ESI): calcd for C_17_H_23_N_2_O_2_ [M + H]^+^, 287.1754; found 287.1735.

#### 2-(((2*S*)-2′,3′-Dihydrospiro[cyclopropane-1,1′-inden]-2-yl)amino)-1-morpholinoethan-1-one hydrochloride (25b)

This compound was synthesized from 8b according to the methodology described for 25a. Yellow solid; yield 45%. ^1^H NMR (400 MHz, CD_3_OD) *δ* 7.36 (d, *J* = 6.4 Hz, 1H), 7.30–7.20 (m, 3H), 4.16–3.78 (m, 2H), 3.66–3.59 (m, 4H), 3.57–3.54 (m, 2H), 3.31–3.30 (m, 2H), 3.22–3.13 (m, 1H), 3.07 (dd, *J* = 7.8, 4.6 Hz, 1H), 3.02–2.96 (m, 1H), 2.40–1.94 (m, 2H), 1.75–1.45 (m, 2H). ^13^C NMR (101 MHz, CD_3_OD) *δ* 164.6, 147.6, 140.1, 129.0, 128.0, 126.5, 121.7, 67.5, 67.3, 46.2, 43.6, 42.7, 36.7, 34.0, 31.5, 15.1. HRMS (ESI): calcd for C_17_H_22_N_2_NaO_2_ [M + Na]^+^, 309.1573; found 309.1550.

#### *tert*-Butyl ((2*R*)-5′-bromo-2′,3′-dihydrospiro[cyclopropane-1,1′-inden]-2-yl)carbamate (27a)

This compound was obtained from 5-bromo-2,3-dihydro-1*H*-inden-1-one (26) as starting material and prepared according to the methodology described for 12a. White solid; yield 24%. ^1^H NMR (400 MHz, CDCl_3_) *δ* 7.31 (s, 1H), 7.21 (d, *J* = 8.0 Hz, 1H), 6.50 (d, *J* = 8.0 Hz, 1H), 4.73 (s, 1H), 3.11–2.95 (m, 2H), 2.69 (s, 1H), 2.14–2.04 (m, 2H), 1.43 (s, 9H), 1.35–1.32 (m, 2H). ^13^C NMR (101 MHz, CDCl_3_) *δ* 156.4, 146.2, 145.4, 129.4, 127.4, 119.9, 119.6, 79.7, 36.5, 32.8, 30.6, 28.9, 28.3, 21.0.

#### *tert*-Butyl ((2*S*)-5′-bromo-2′,3′-dihydrospiro[cyclopropane-1,1′-inden]-2-yl)carbamate (27b)

This compound was obtained from 5-bromo-2,3-dihydro-1*H*-inden-1-one (26) as starting material and prepared according to the methodology described for 12b. White solid; yield 22%. ^1^H NMR (400 MHz, CDCl_3_) *δ* 7.35 (s, 1H), 7.24 (d, *J* = 8.4 Hz, 1H), 6.68 (s, 1H), 4.56 (s, 1H), 3.17–2.91 (m, 2H), 2.75 (s, 1H), 2.30–1.91 (m, 2H), 1.31–1.22 (m, 9H), 1.14 (s, 2H). ^13^C NMR (101 MHz, CDCl_3_) *δ* 156.1, 148.0, 141.6, 128.6, 127.5, 122.5, 119.9, 79.4, 37.2, 36.1, 34.9, 30.6, 28.2, 19.3.

### General procedure for the synthesis of (2*R*)-5′-phenyl-2′,3′-dihydrospiro[cyclopropane-1,1′-inden]-2-amine hydrochloride (28a)

To a mixture of 27a (150 mg, 0.44 mmol) and tetrakis(triphenylphosphine)palladium (25 mg, 0.022 mmol) in DMF (2.5 mL) was added phenylboronic acid (65 mg, 0.53 mmol) immediately followed by aqueous Na_2_CO_3_ solution (2 M, 0.4 mL H_2_O). The mixture was stirred at 80 °C for 16 h under an argon atmosphere. After cooling, the reaction mixture was filtered and the filtrate was extracted with EtOAc (3 × 30 mL). The combined organic layers were washed with water, dried over MgSO_4_, filtered, and concentrated *in vacuo*. The crude product was purified by flash chromatography in PE/EtOAc (25 : 1) to yield the pure product as a white solid. A solution of HCl in ethyl acetate (4 M, 5 mL) was then added to the white solid. After stirring at room temperature overnight, the precipitated solid was filtered off, washed with diethyl ether and dried to afford 28a as a hydrochloride salt. Yellow solid; yield 63%. ^1^H NMR (400 MHz, DMSO-*d*_6_) *δ* 8.73 (s, 2H), 7.63–7.61 (m, 2H), 7.51 (s, 1H), 7.46–7.42 (m, 3H), 7.36–7.32 (m, 1H), 6.93 (d, *J* = 8.0 Hz, 1H), 3.18–3.04 (m, 2H), 2.85–2.82 (m, 1H), 2.37–2.17 (m, 2H), 1.38–1.35 (m, 2H). ^13^C NMR (101 MHz, DMSO-*d*_6_) *δ* 144.4, 144.3, 140.4, 139.0, 128.9, 127.2, 126.7, 125.4, 122.5, 119.8, 33.6, 30.3, 30.1, 28.3, 18.8. HRMS (ESI): calcd for C_17_H_18_N [M + H]^+^, 236.1439; found 236.1425.

#### (2*S*)-5′-Phenyl-2′,3′-dihydrospiro[cyclopropane-1,1′-inden]-2-amine hydrochloride (28b)

This compound was obtained from 27b and phenylboronic acid according to the methodology described for 28a. Yellow solid; yield 62%. ^1^H NMR (400 MHz, DMSO-*d*_6_) *δ* 8.36 (s, 2H), 7.63 (d, *J* = 7.2 Hz, 2H), 7.57 (s, 1H), 7.46–7.43 (m, 3H), 7.38–7.31 (m, 2H), 3.13–2.92 (m, 2H), 2.87–2.84 (m, 1H), 2.26–1.96 (m, 2H), 1.56–1.33 (m, 2H). ^13^C NMR (101 MHz, DMSO-*d*_6_) *δ* 146.8, 140.5, 139.9, 139.4, 129.1, 127.5, 126.8, 125.1, 123.2, 122.2, 35.1, 34.0, 31.2, 30.5, 16.0. HRMS (ESI): calcd for C_17_H_18_N [M + H]^+^, 236.1439; found 236.1434.

#### (2*R*)-5′-(4-Fluorophenyl)-2′,3′-dihydrospiro[cyclopropane-1,1′-inden]-2-amine hydrochloride (29a)

This compound was obtained from 27a and (4-fluorophenyl)boronic acid according to the methodology described for 28a. White solid; yield 60%. ^1^H NMR (400 MHz, DMSO-*d*_6_) *δ* 8.77 (s, 2H), 7.68–7.64 (m, 2H), 7.49 (s, 1H), 7.40 (d, *J* = 8.0 Hz, 1H), 7.26 (t, *J* = 8.8 Hz, 2H), 6.92 (d, *J* = 8.0 Hz, 1H), 3.17–3.03 (m, 2H), 2.83 (t, *J* = 6.2 Hz 1H), 2.38–2.17 (m, 2H), 1.36–1.33 (m, 2H). ^13^C NMR (101 MHz, DMSO-*d*_6_) *δ* 161.6 (d, *J*_C–F_ = 244.9 Hz), 144.3, 144.2, 137.8, 136.7 (d, *J*_C–F_ = 3.0 Hz). 128.4 (d, *J*_C–F_ = 8.1 Hz), 125.2, 122.4, 119.7, 115.5 (d, *J*_C–F_ = 21.3 Hz), 33.4, 30.2, 29.9, 28.2, 18.6. HRMS (ESI): calcd for C_17_H_17_NF [M + H]^+^, 254.1345; found 254.1336.

#### (2*S*)-5′-(4-Fluorophenyl)-2′,3′-dihydrospiro[cyclopropane-1,1′-inden]-2-amine hydrochloride (29b)

This compound was obtained from 27b and (4-fluorophenyl)boronic acid according to the methodology described for 28a. White solid; yield 34%. ^1^H NMR (400 MHz, DMSO-*d*_6_) *δ* 8.52 (s, 2H), 7.71–7.67 (m, 2H), 7.56 (s, 1H), 7.48–7.43 (m, 2H), 7.28 (t, *J* = 8.8 Hz, 2H), 3.12–2.94 (m, 2H), 2.86 (s, 1H), 2.27–1.98 (m, 2H), 1.64–1.32 (m, 2H). ^13^C NMR (101 MHz, DMSO-*d*_6_) *δ* 161.6 (d, *J*_C–F_ = 244.9 Hz), 146.5, 139.7, 138.0, 136.7 (d, *J*_C–F_ = 3.0 Hz), 128.4 (d, *J*_C–F_ = 8.2 Hz), 124.7, 122.8, 122.2, 115.6 (d, *J*_C–F_ = 21.4 Hz), 34.8, 33.7, 31.0, 30.3, 15.8. HRMS (ESI): calcd for C_17_H_17_FN [M + H]^+^,254.1340; found 254.1337.

#### (2*R*)-*N*-(5-Fluoro-2-methoxybenzyl)-5′-phenyl-2′,3′-dihydrospiro[cyclopropane-1,1′-inden]-2-amine (30a)

This compound was obtained from 28a and 5-fluoro-2-methoxybenzaldehyde according to the methodology described for 13a. Yellow oil; yield 73%. ^1^H NMR (400 MHz, CDCl_3_) *δ* 7.55 (d, *J* = 7.6 Hz, 2H), 7.42–7.38 (m, 3H), 7.34–7.28 (m, 2H), 7.00 (dd, *J* = 8.8, 3.2 Hz, 1H), 6.92–6.87 (m, 1H), 6.73 (dd, *J* = 9.2, 4.4 Hz, 1H), 6.66 (d, *J* = 8.0 Hz, 1H), 3.90–3.72 (m, 6H), 3.14–2.99 (m, 2H), 2.47–2.41 (m, 1H), 2.34 (dd, *J* = 7.2, 4.8 Hz, 1H), 2.22–2.14 (m, 1H), 1.21–0.91 (m, 2H). ^13^C NMR (101 MHz, CDCl_3_) *δ* 156.0 (d, *J*_C–F_ = 239.4 Hz), 152.6 (d, *J*_C–F_ = 2.1 Hz), 146.1, 143.5, 140.6, 138.2, 128.9 (d, *J*_C–F_ = 6.9 Hz), 127.6, 126.0, 125.8, 124.5, 122.1, 117.7, 115.6 (d, *J*_C–F_ = 23.2 Hz), 112.7 (d, *J*_C–F_ = 22.7 Hz), 109.9 (d, *J*_C–F_ = 8.2 Hz), 54.6, 47.7, 43.6, 32.4, 29.8, 27.5, 21.0. HRMS (ESI): calcd for C_25_H_25_FNO [M + H]^+^, 374.1915; found 374.1920.

#### (2*S*)-*N*-(5-Fluoro-2-methoxybenzyl)-5′-phenyl-2′,3′-dihydrospiro[cyclopropane-1,1′-inden]-2-amine (30b)

This compound was obtained from 28b and 5-fluoro-2-methoxybenzaldehyde according to the methodology described for 13a. Yellow oil; yield 55%. ^1^H NMR (400 MHz, CDCl_3_) *δ* 7.60–7.58 (m, 2H), 7.43–7.39 (m, 3H), 7.37–7.35 (m, 1H), 7.32–7.28 (m, 1H), 7.17 (d, *J* = 8.0 Hz, 1H), 6.87–6.80 (m, 2H), 6.66 (dd, *J* = 8.4, 4.4 Hz, 1H), 3.72 (s, 3H), 3.68–3.45 (m, 2H), 3.09–2.95 (m, 2H), 2.43 (dd, *J* = 7.2, 4.8 Hz, 1H), 2.24–2.16 (m, 1H), 1.97 (s, 1H), 1.92–1.86 (m, 1H), 1.10–1.03 (m, 2H). ^13^C NMR (101 MHz, CDCl_3_) *δ* 156.8 (d, *J*_C–F_ = 239.1 Hz), 153.5 (d, *J*_C–F_ = 2.0 Hz), 145.8, 143.4, 141.8, 139.0, 130.2 (d, *J*_C–F_ = 6.7 Hz), 128.7, 127.1, 126.8, 124.7, 122.8, 122.6, 116.6 (d, *J*_C–F_ = 23.1 Hz), 113.5 (d, *J*_C–F_ = 22.8 Hz), 110.7 (d, *J*_C–F_ = 8.2 Hz), 55.7, 48.4, 44.8, 35.7, 33.7, 30.8, 20.8. HRMS (ESI): calcd for C_25_H_25_FNO [M + H]^+^, 374.1915; found 374.1906.

#### (2*R*)-*N*-(2-Chloro-3,4-dimethoxybenzyl)-5′-phenyl-2′,3′-dihydrospiro[cyclopropane-1,1′-inden]-2-amine (31a)

This compound was obtained from 28a and 2-chloro-3,4-dimethoxybenzaldehyde according to the methodology described for 13a. Yellow oil; yield 52%. ^1^H NMR (400 MHz, CDCl_3_) *δ* 7.56–7.54 (m, 2H), 7.42–7.39 (m, 3H), 7.34–7.28 (m, 2H), 7.06 (d, *J* = 8.4 Hz, 1H), 6.76 (d, *J* = 8.4 Hz, 1H), 6.66 (d, *J* = 8.0 Hz, 1H), 3.97–3.88 (m, 2H), 3.84 (s, 6H), 3.14–2.99 (m, 2H), 2.48–2.41 (m, 1H), 2.37 (dd, *J* = 7.2, 4.8 Hz, 1H), 2.23–2.16 (m, 1H), 1.22–0.95 (m, 2H). ^13^C NMR (101 MHz, CDCl_3_) *δ* 153.0, 146.9, 145.7, 144.5, 141.6, 139.3, 130.1, 128.7, 128.4, 127.1, 126.8, 125.6, 125.3, 123.1, 118.7, 110.4, 60.6, 56.1, 51.1, 44.2, 33.4, 30.9, 28.6, 21.9. HRMS (ESI): calcd for C_26_H_27_ClNO_2_ [M + H]^+^, 420.1725; found 420.1685.

#### (2*R*)-*N*-(5-Fluoro-2-methoxybenzyl)-5′-(4-fluorophenyl)-2′,3′-dihydrospiro[cyclopropane-1,1′-inden]-2-amine (32a)

This compound was obtained from 28a and 5-fluoro-2-methoxybenzaldehyde according to the methodology described for 13a. Yellow oil; yield 54%. ^1^H NMR (400 MHz, CDCl_3_) *δ* 7.52–7.47 (m, 2H), 7.36 (s, 1H), 7.28–7.24 (m, 1H), 7.12–7.06 (m, 2H), 7.02 (dd, *J* = 8.8, 3.2 Hz, 1H), 6.93–6.88 (m, 1H), 6.74 (dd, *J* = 8.8, 4.4 Hz, 1H), 6.65 (d, *J* = 7.6 Hz, 1H), 3.91–3.76 (m, 2H), 3.73 (s, 3H), 3.13–2.98 (m, 2H), 2.61 (s, 1H), 2.48–2.41 (m, 1H), 2.36 (dd, *J* = 7.2, 4.8 Hz, 1H), 2.23–2.16 (m, 1H), 1.22–0.97 (m, 2H). ^13^C NMR (101 MHz, CDCl_3_) *δ* 162.2 (d, *J*_C–F_ = 246.6 Hz), 156.9 (d, *J*_C–F_ = 239.5 Hz), 153.7 (d, *J*_C–F_ = 2.0 Hz), 147.0, 144.6, 138.3, 137.7 (d, *J*_C–F_ = 3.2 Hz), 129.4 (d, *J*_C–F_ = 7.4 Hz), 128.5 (d, *J*_C–F_ = 8.0 Hz), 125.4, 123.0, 118.8, 116.8 (d, *J*_C–F_ = 23.2 Hz), 115.5 (d, *J*_C–F_ = 21.4 Hz), 114.0 (d, *J*_C–F_ = 22.8 Hz), 110.9 (d, *J*_C–F_ = 8.1 Hz), 55.7, 48.6, 44.4, 33.3, 30.9, 28.5, 21.8. HRMS (ESI): calcd for C_25_H_24_F_2_NO [M + H]^+^, 392.1820; found 392.1801.

### LSD1 and LSD2 inhibition assay

Recombinant human LSD1 (172–852 aa) and full-length LSD2 were purchased from Sino Biological Inc. and Active Motif. LSD1 or LSD2 enzyme inhibition assay was performed with Lance Ultra LSD1 Histone H3-Lysine 4 Demethylase Assay kit (Perkin Elmer) according to the manufacturer's protocol. Briefly, LSD1/2 assay components were diluted in Tris buffer (50 mM Tris–HCl, 50 mM NaCl, 0.01% Tween-20, 1 mM DTT, 10 μM FAD, 10% glycerol, pH 9.0), and cultured for 1 hour at room temperature, which contained 4 μL enzyme solution (4 nM LSD1 or 172 nM LSD2), 4 μL substrate solution (2.5 μM Bio-H3K4me2(1–24aa)), and 2 μL inhibitor in 384-well microplate. And then, 5 μL detection mix containing Eu-labeled H3K4me0 antibody and ULight-Streptavidin were added, and fluorescence intensity were measured with Envision (PerkinElmer) in TR-FRET mode (excitation at 320 & emission at 665 nm). The test was carried out in triplicate. And the IC_50_ data was calculated using the software GraphPad Prism 5, and chosen the equation “sigmoidal dose–response (variable slope)” for curve fitting.

### MAO-A and MAO-B inhibition assay

MAO-A and MAO-B enzymes were purchased from Sigma-Aldrich and the inhibition assay was tested with Promega MAO-Glo™ Assay kit according to the manufacturer's protocol. The luminescent signals were detected using Envision (PerkinElmer). The test was carried out in triplicate. And the IC_50_ data was calculated using the software GraphPad Prism 5, and chosen the equation “sigmoidal dose–response (variable slope)” for curve fitting.

## Conflicts of interest

There are no conflicts of interest to declare.

## Supplementary Material

RA-008-C7RA13097J-s001
