# Supplementary material for: Novel spirocyclic tranylcypromine derivatives as lysine-specific demethylase 1 (LSD1) inhibitors
Source: RSC Adv. 2018 Jan 5;8(3):1666–76. doi: 10.1039/c7ra13097j (PMC9077246; doi:10.1039/c7ra13097j)
Supplement: RA-008-C7RA13097J-s001 [file RA-008-C7RA13097J-s001.pdf]

Electronic Supplementary Material (ESI) for RSC Advances.
This journal is © The Royal Society of Chemistry 2017

Supplementary Information

Novel Spirocyclic Tranylcypromine Derivatives as LSD1 Inhibitors

Ying Shi[a, +] , Yan-Ran Wu[a, +], Ming-Bo Su[b, +], Dong-Hao Shen[a], Hendra Gunosewoyo[c], Fan Yang[a], Jia Li[b], Jie Tang[d], Yu-Bo Zhou[b, *], and Li-Fang Yu[a *]

*Corresponding authors

[a] Shanghai Engineering Research Center of Molecular Therapeutics and New Drug Development, School of Chemistry and Molecular Engineering, East China Normal University, 3663 North Zhongshan Road, Shanghai 200062, China

[b] CAS Key Laboratory of Receptor Research, National Center for Drug Screening, Shanghai Institute of Materia Medica, Chinese Academy of Sciences, 189 Guo Shou Jing Road, Shanghai 201203, China

[c] School of Pharmacy, Faculty of Health Sciences, Curtin University, Bentley, Perth, WA 6102, Australia

[d] Shanghai Key Laboratory of Green Chemistry and Chemical Process, School of Chemistry and Molecular Engineering, East China Normal University, 3663 North Zhongshan Road, Shanghai 200062, China

[+]These authors contributed equally

Contents

Table 1
^{1}H NMR, ^{13}C NMR spectrum for 8a, 8b,13a-25a,13b-25b,28a-32a,28b-30b
^{1}H-^{1}H COESY and ^{1}H-^{1}H NOESY spectrum for 8a,8b

Table 1. NMR Data of **8a** and **8b** in CD_3OD (400 MHz).

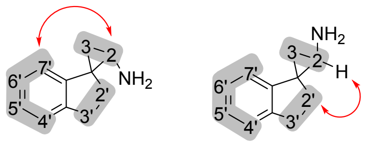

8a **8b**

(Gray Zones ^{1}H, ^{1}H COSY; Red Arrows ^{1}H, ^{1}H NOESY).

**Cmpd**	**Pos.**	**Type**	**δ_H**	**m (*J* in Hz)**	**COSY**	**NOESY**
**8a**	2’	CH_2	2.35–2.20	m	3’	3’
	3’	CH_2	3.14	t (7.6)	2’	2’, 4’
	4’	CH	7.22	d (6.4)	5’	3’, 5’
	5’	CH	7.18–7.12	m	4’, 6’	4’
	6’	CH	7.18–7.12	m	5’, 7’	7’
	7’	CH	6.76	d (6.4)	6’	**2**, 3, 6,
	2	CH	2.88	dd (7.2, 4.8)	3	3, **7’**
	3	CH_2 (H, H)	1.42, 1.29	t (7.2), t (5.4)	2	2, 7’
**8b**	2’	CH_2 (H, H)	2.38–2.30, 1.97	m, dd (12.6, 7.8)	3’	**2**, 3’
	3’	CH_2 (H, H)	3.18–3.10, 2.97	m, dd (15.6, 8.8)	2’	2’
	4’	CH	7.32	d (5.2)	5’	5’
	5’	CH	7.26–7.21	m	4’, 6’	4’
	6’	CH	7.26–7.21	m	5’, 7’	7’
	7’	CH	7.08–7.06	m	6’	3, 6’
	2	CH	2.88	dd (7.8, 4.6)	3	**2’**, 3
	3	CH_2 (H, H)	1.51, 1.43	dd (6.8, 4.8), t (7.4)	2	2, 7’

^{1}H NMR (400 MHz, CD_3OD) and ^{13}C NMR (101MHz, CD_3OD) spectrum for 8a

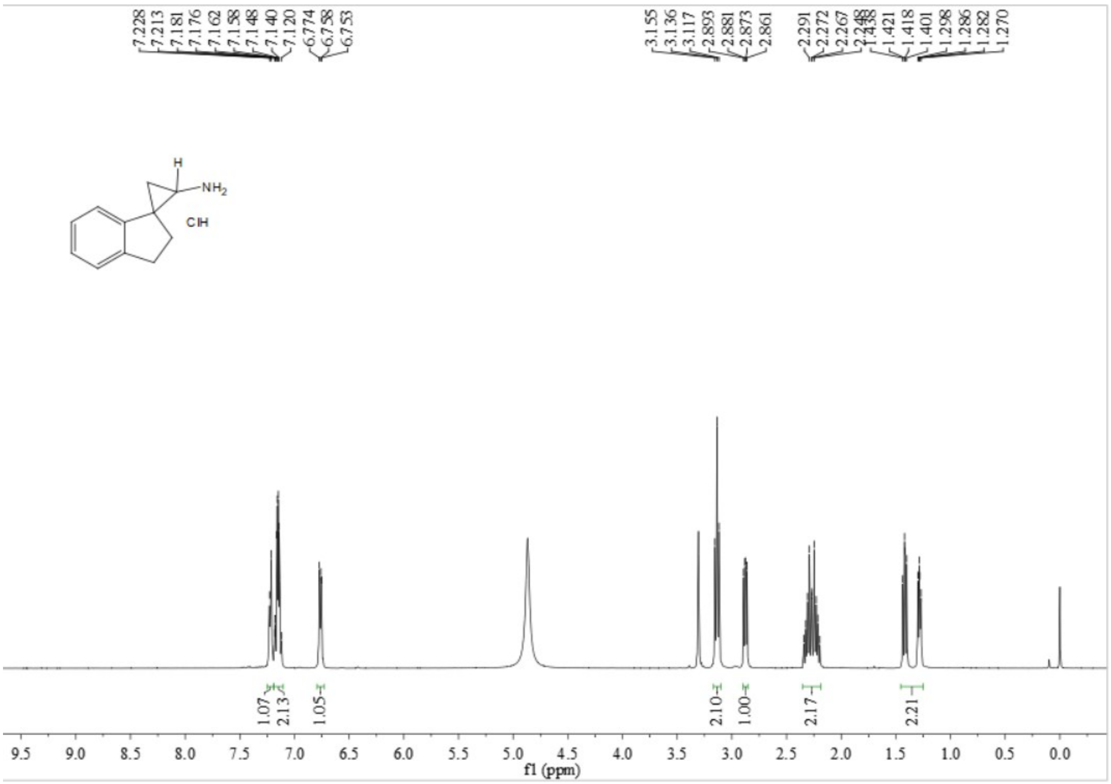

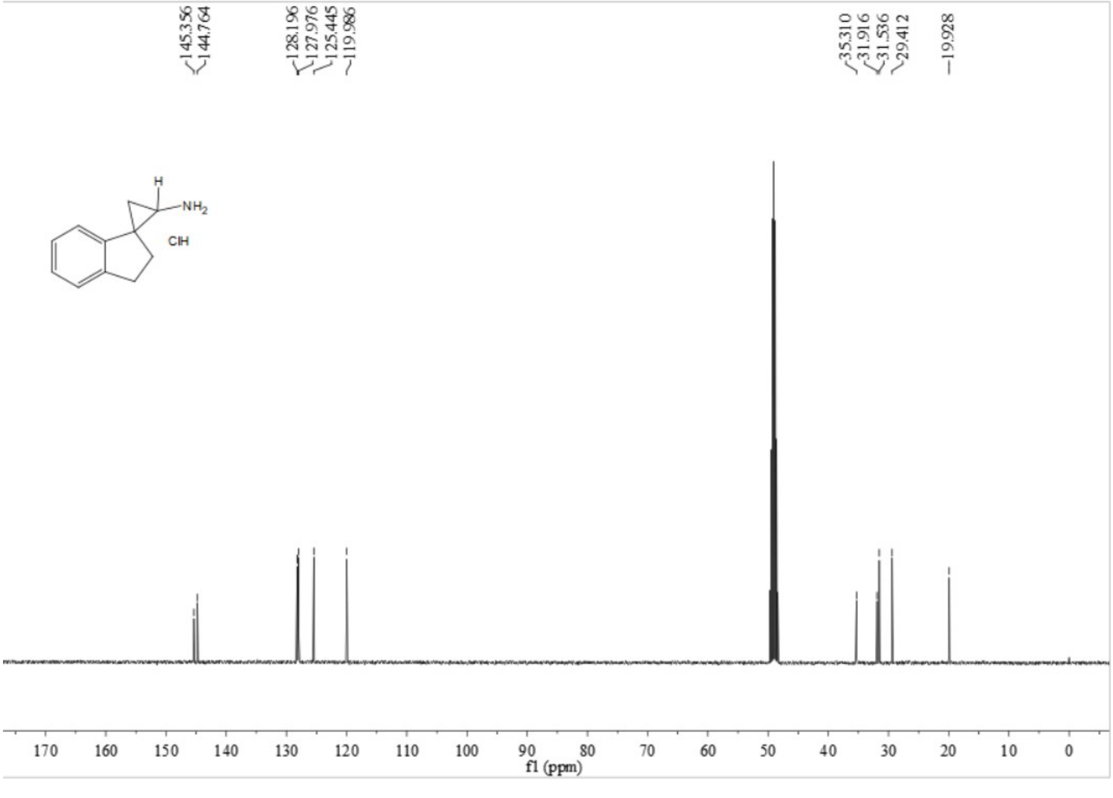

^{1}H-^{1}H COESY (CD_3OD) spectrum for 8a

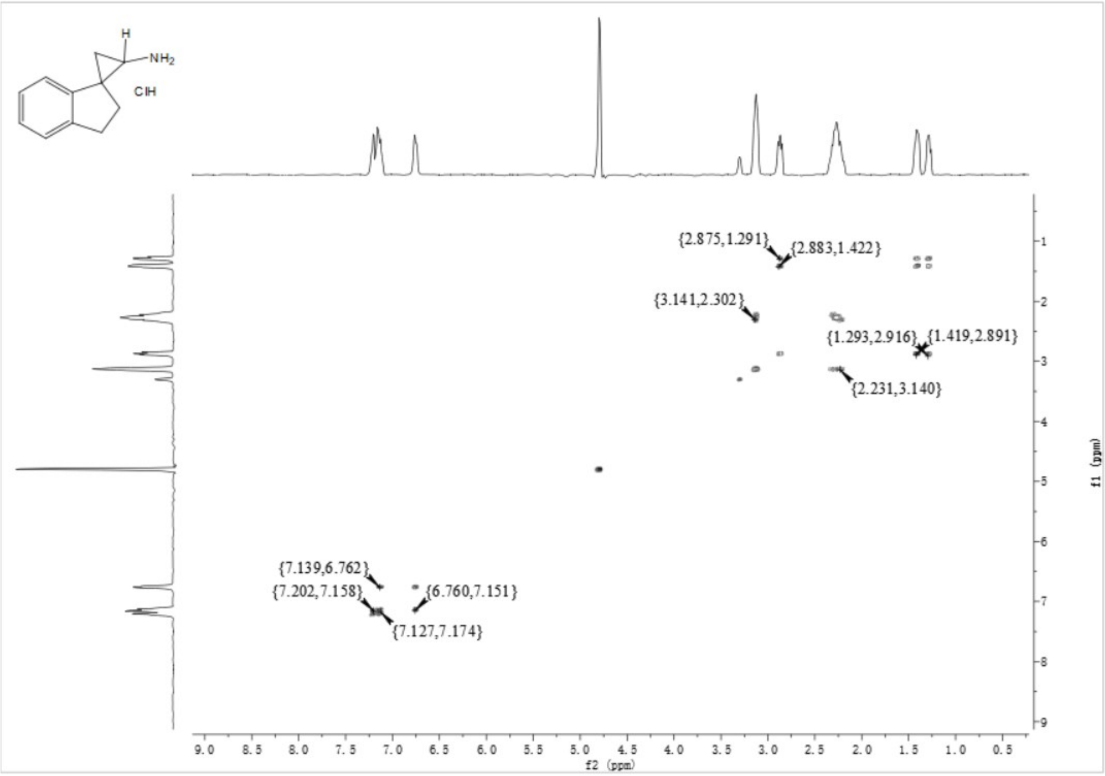

^{1}H-^{1}H NOESY (CD_3OD) spectrum for 8a

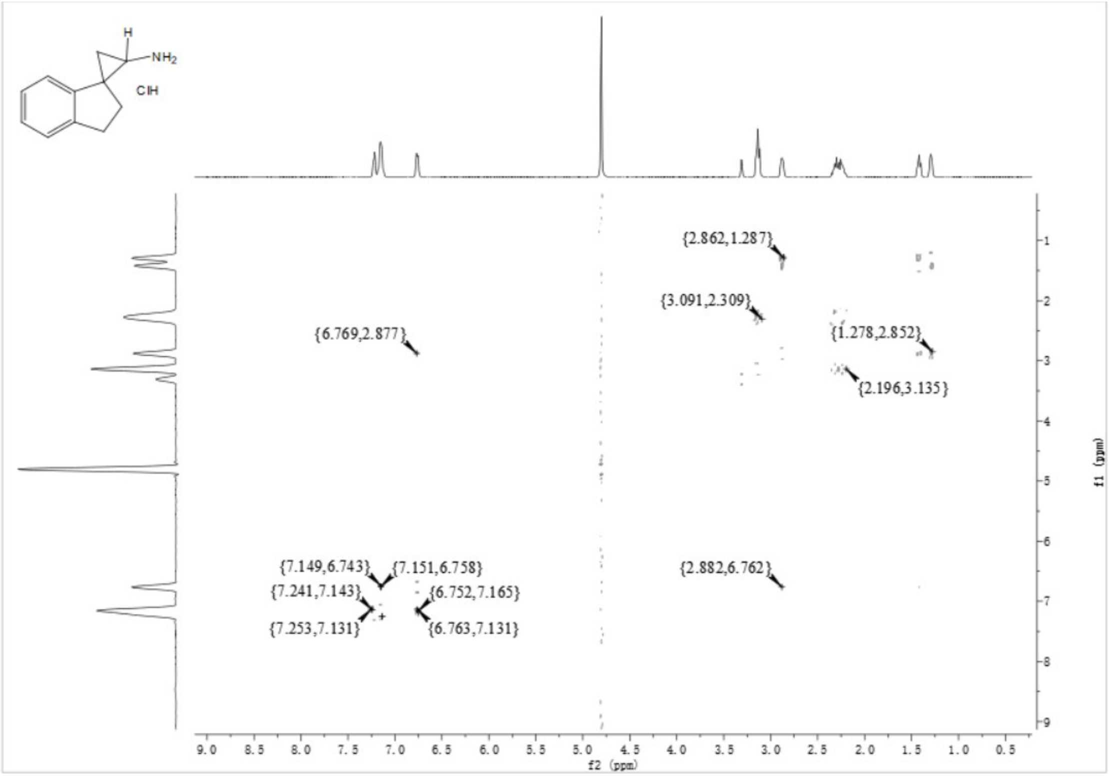

^{1}H NMR (400 MHz, CD_3OD) and ^{13}C NMR (101MHz, CD_3OD) spectrum for 8b

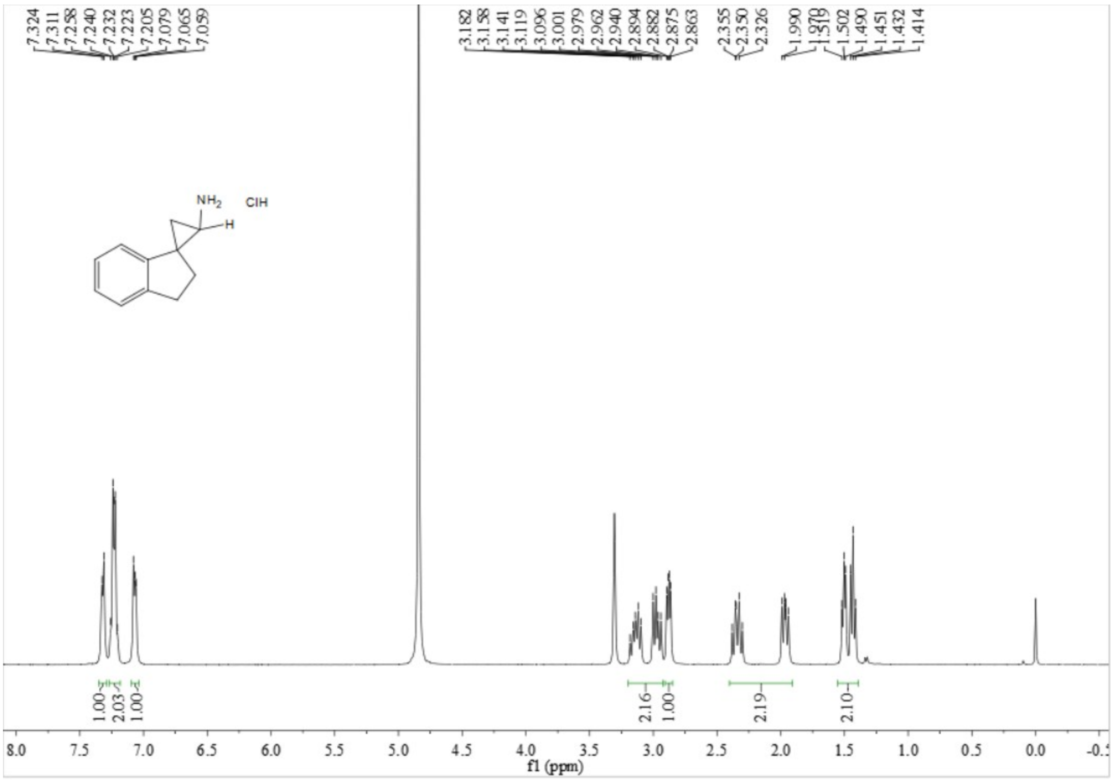

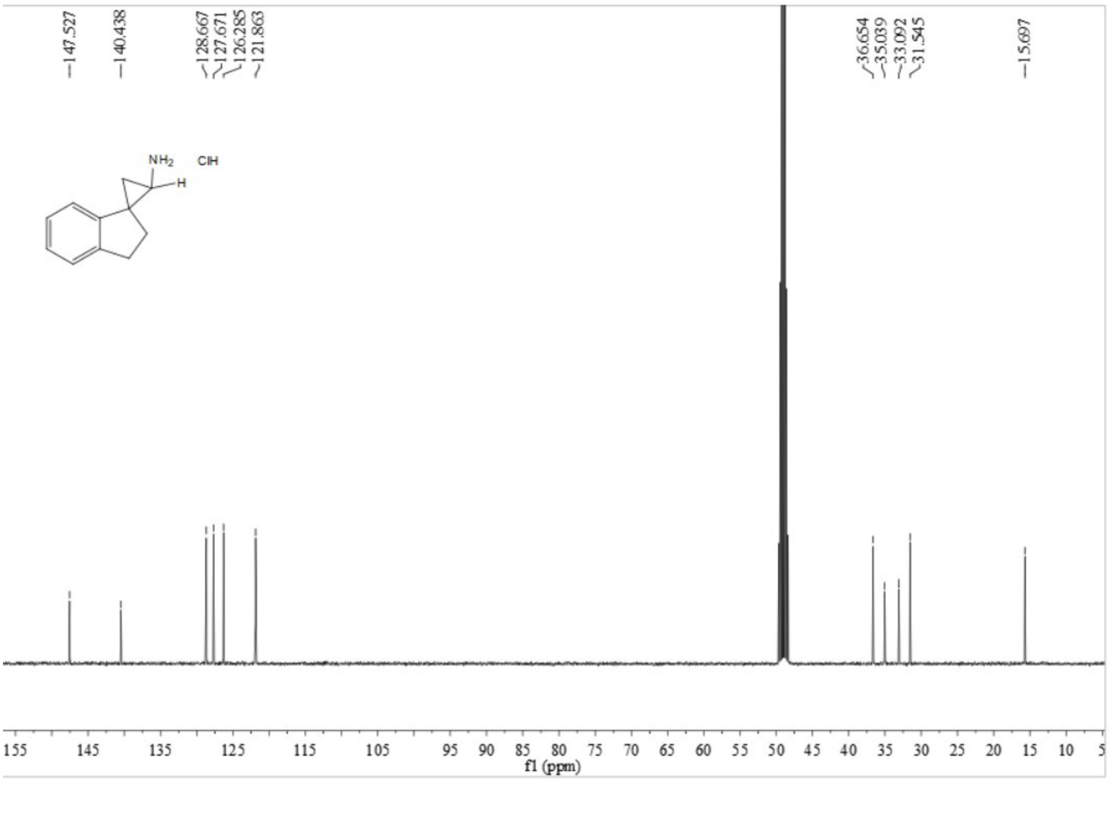

^{1}H-^{1}H COESY (CD_3OD) spectrum for 8b

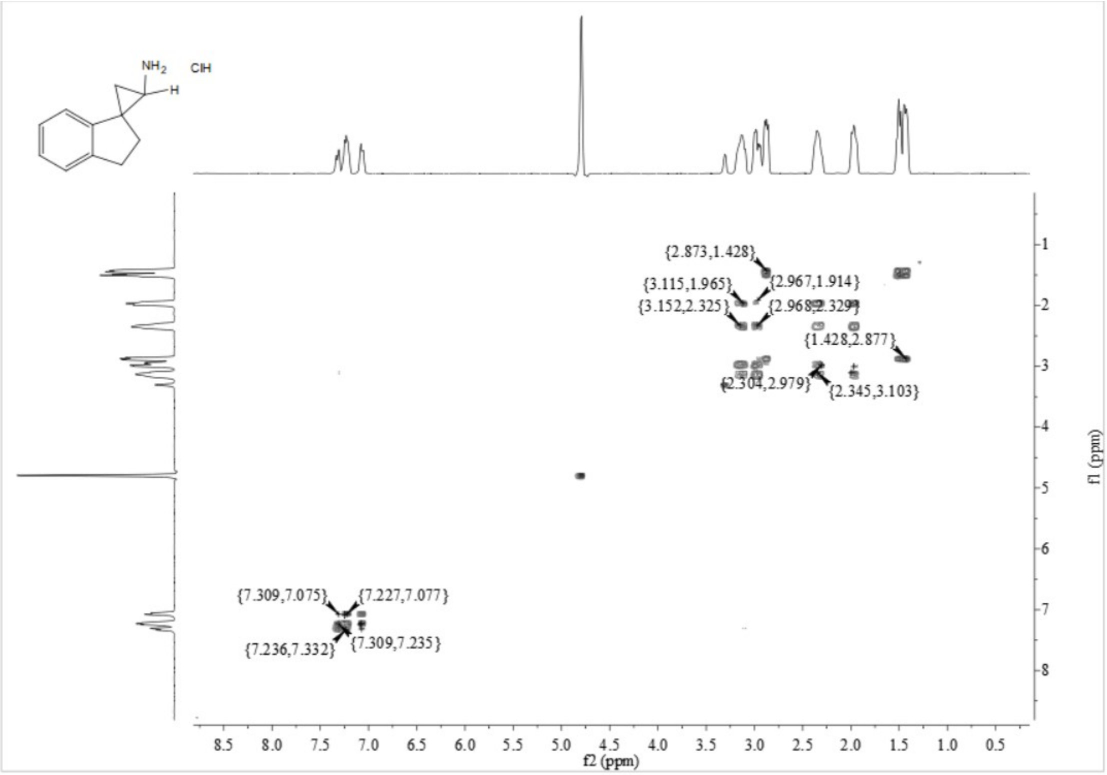

^1H-^1H NOESY (CD_3OD) spectrum for 8b

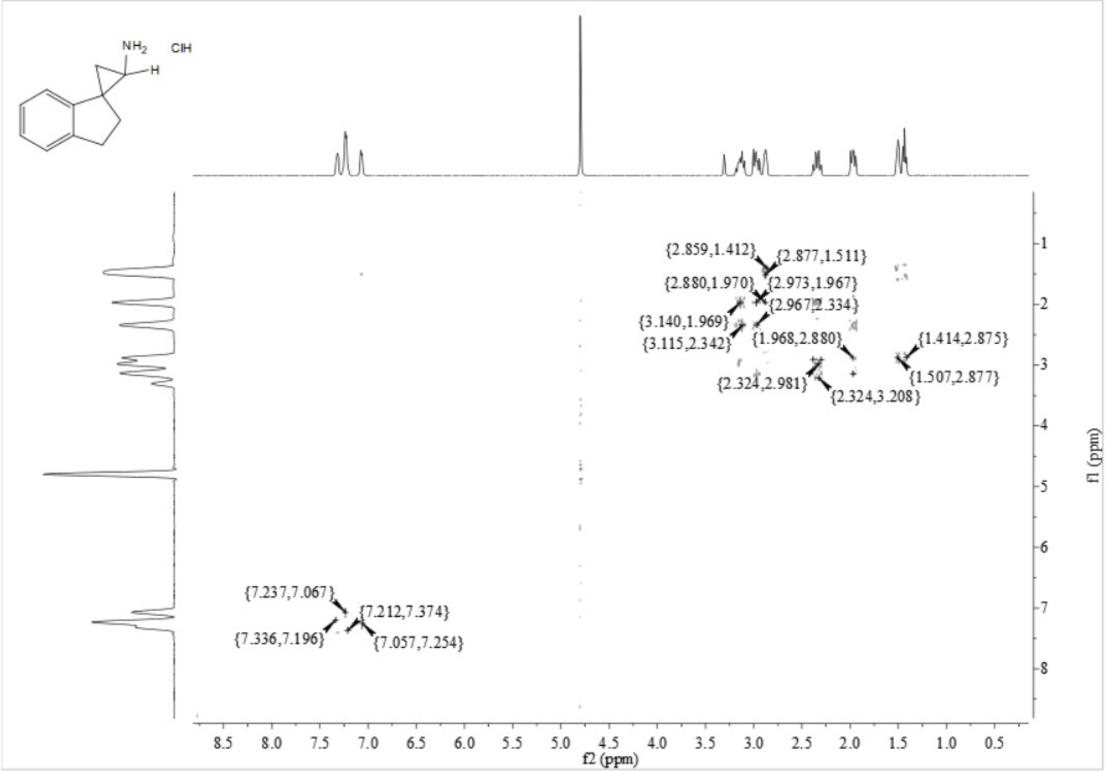

^1H NMR (400 MHz, $CDCl_3$) and ^{13}C NMR (101MHz, $CDCl_3$) spectrum for 13a

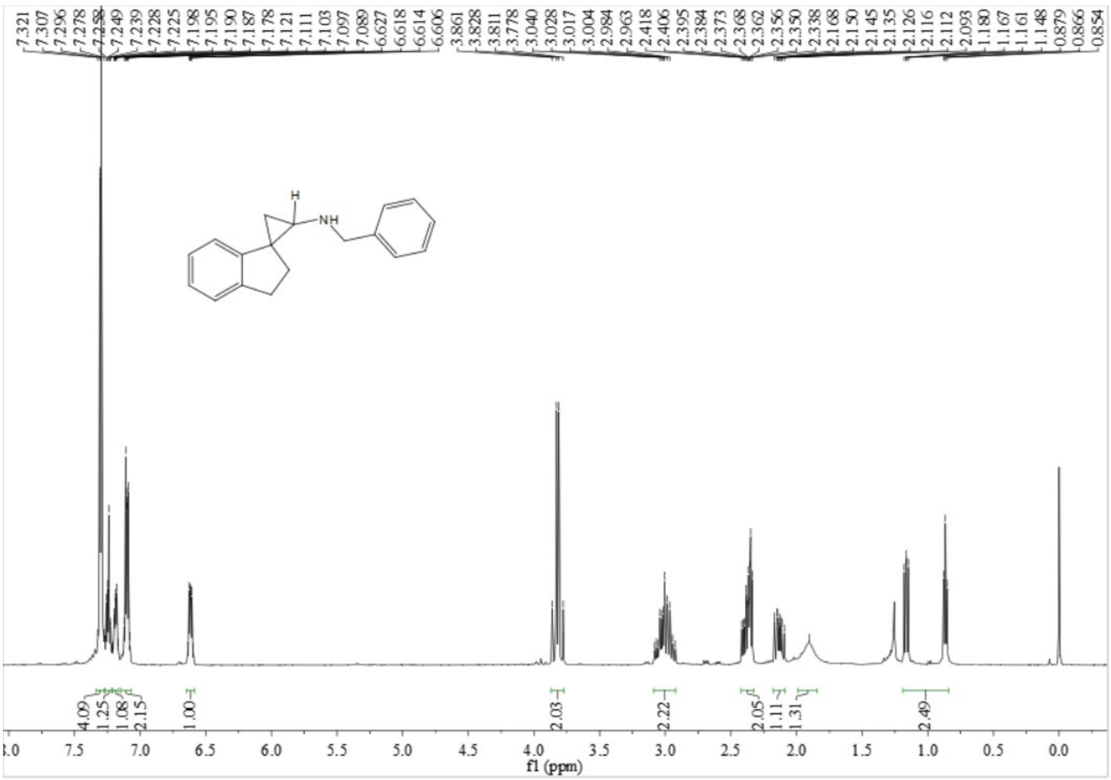

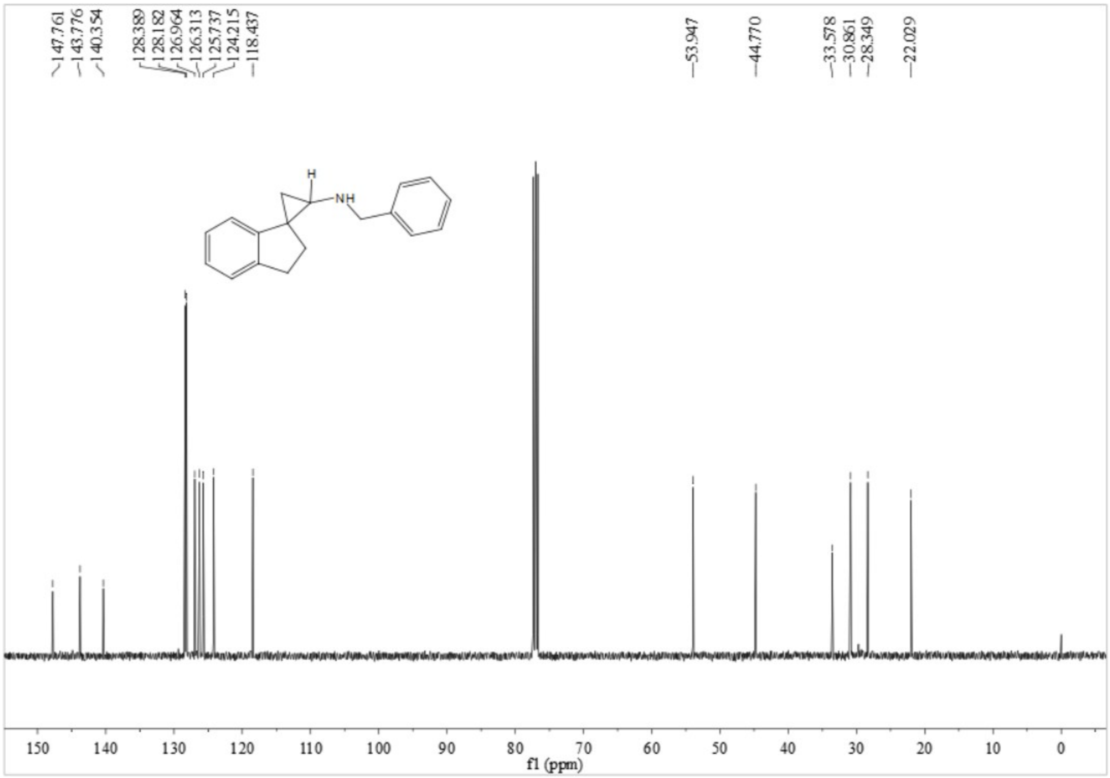

^{1}H NMR (400 MHz, $CDCl_3$) and ^{13}C NMR (101MHz, $CDCl_3$) spectrum for 13b

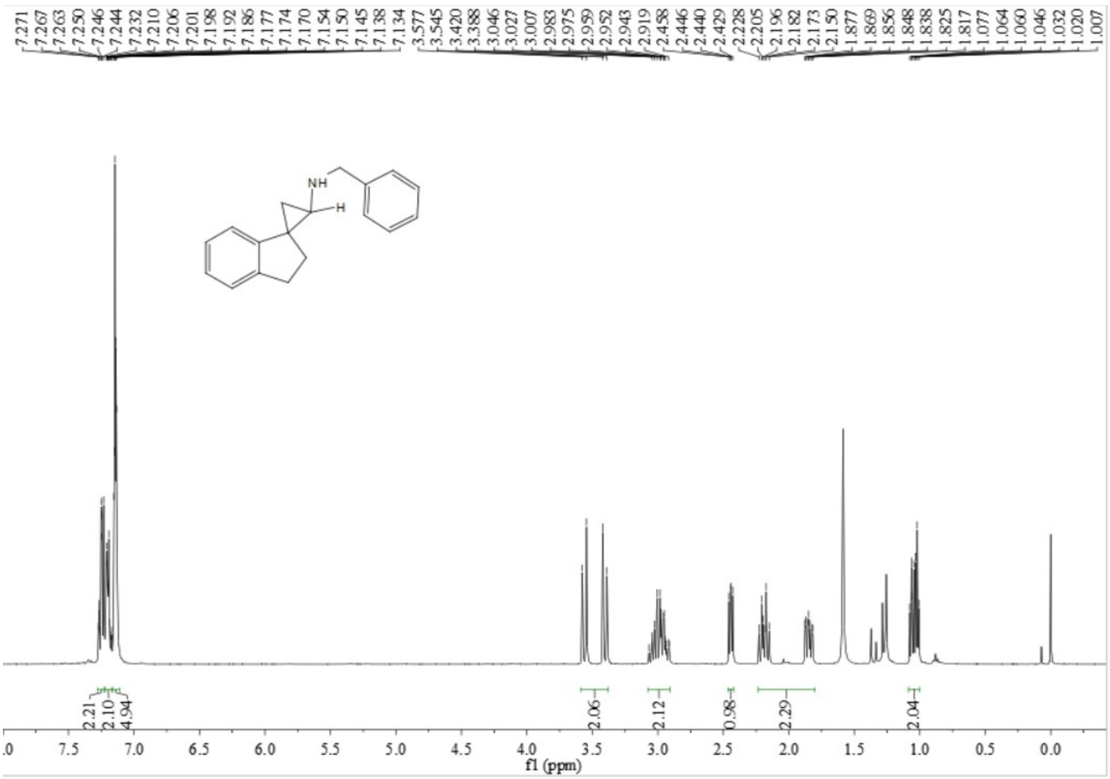

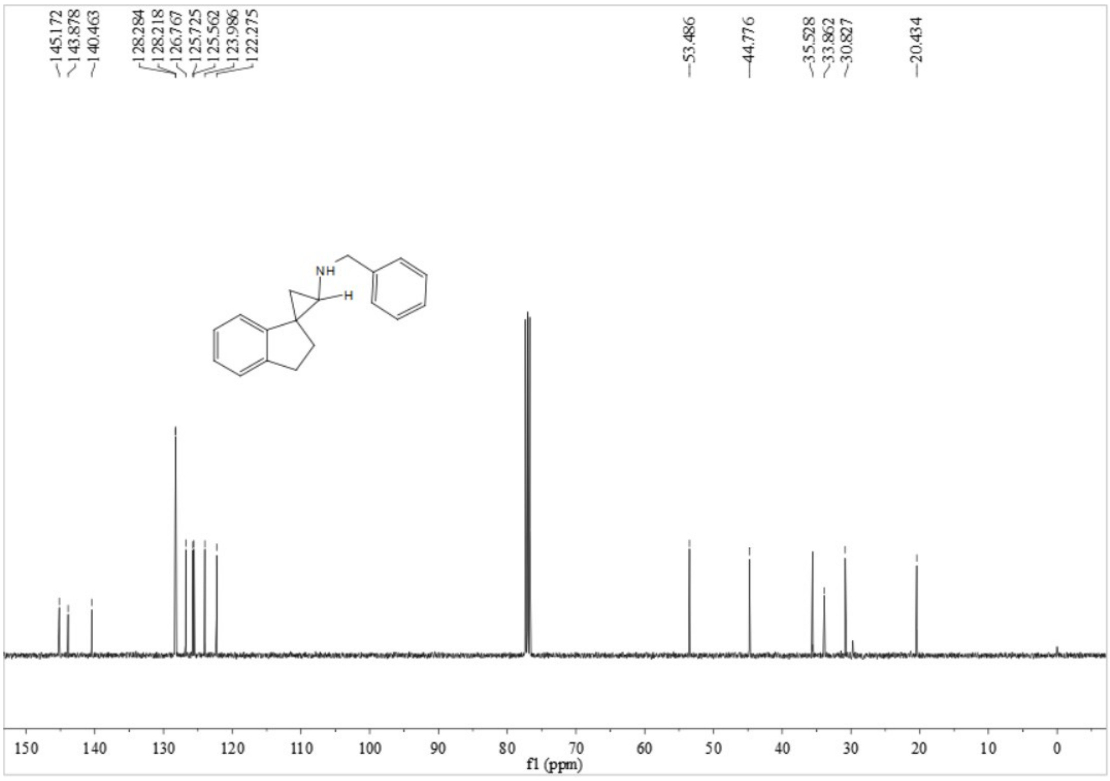

^{1}H NMR (400 MHz, CDCl$_3$) and ^{13}C NMR (101MHz, CDCl$_3$) spectrum for 14a

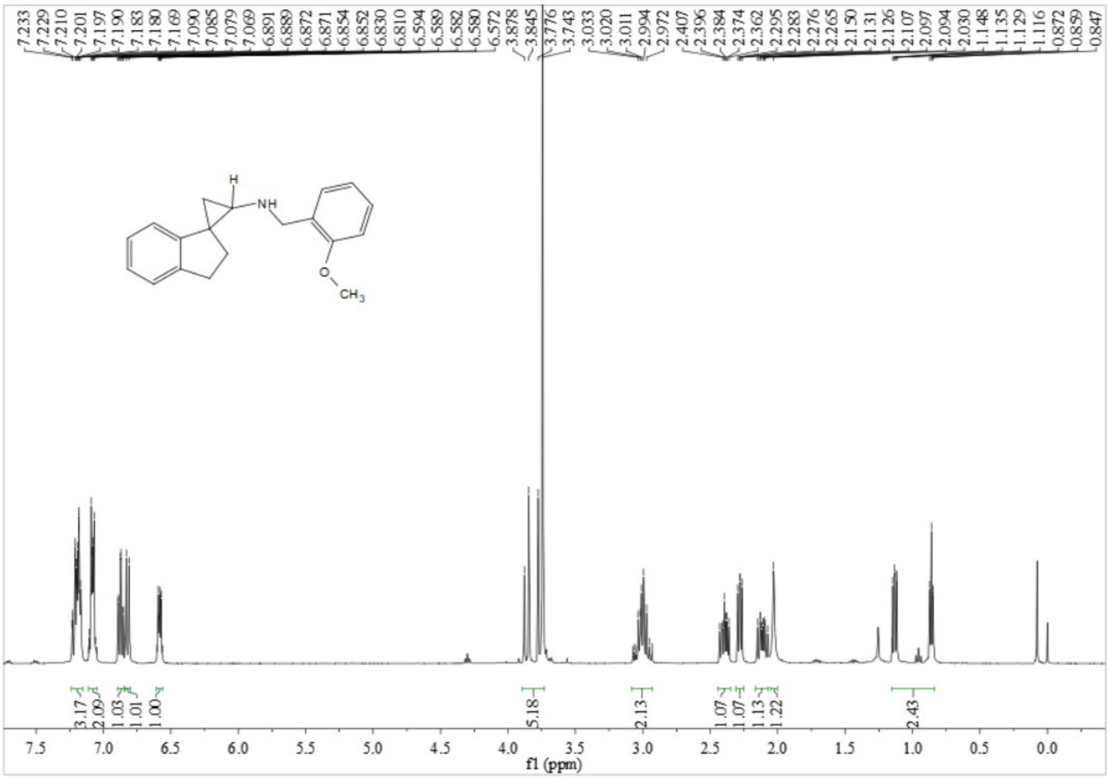

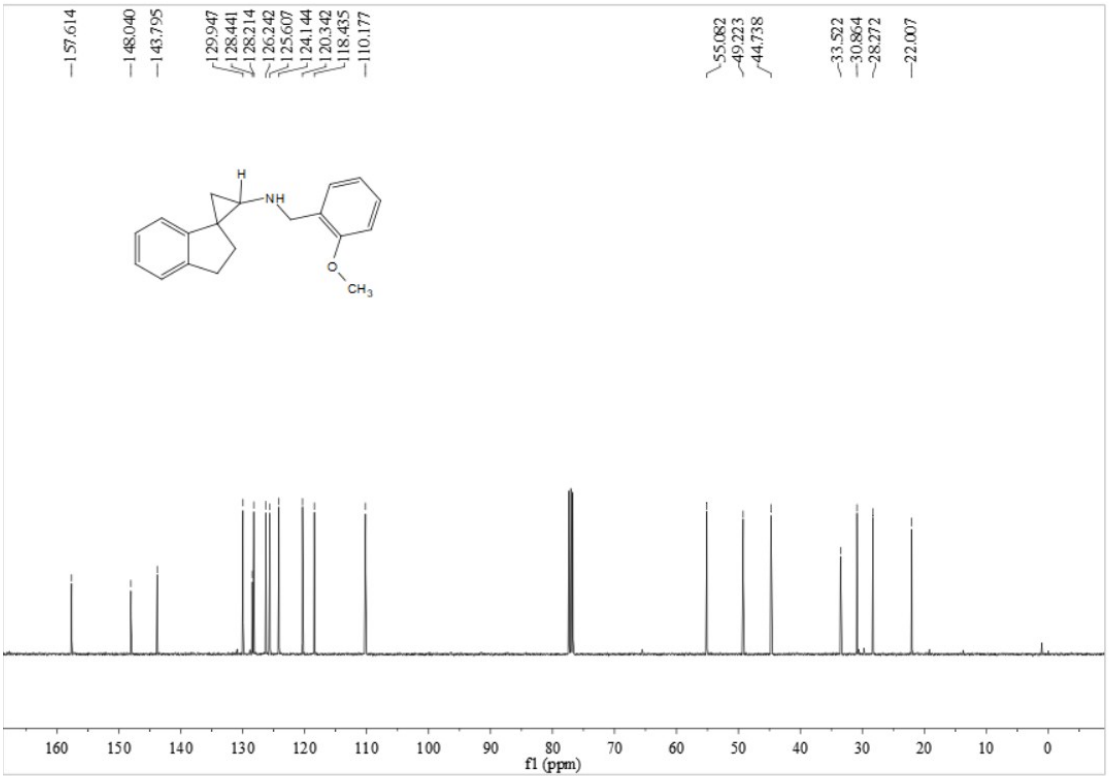

^{1}H NMR (400 MHz, $CDCl_3$) and ^{13}C NMR (101MHz, $CDCl_3$) spectrum for 14b

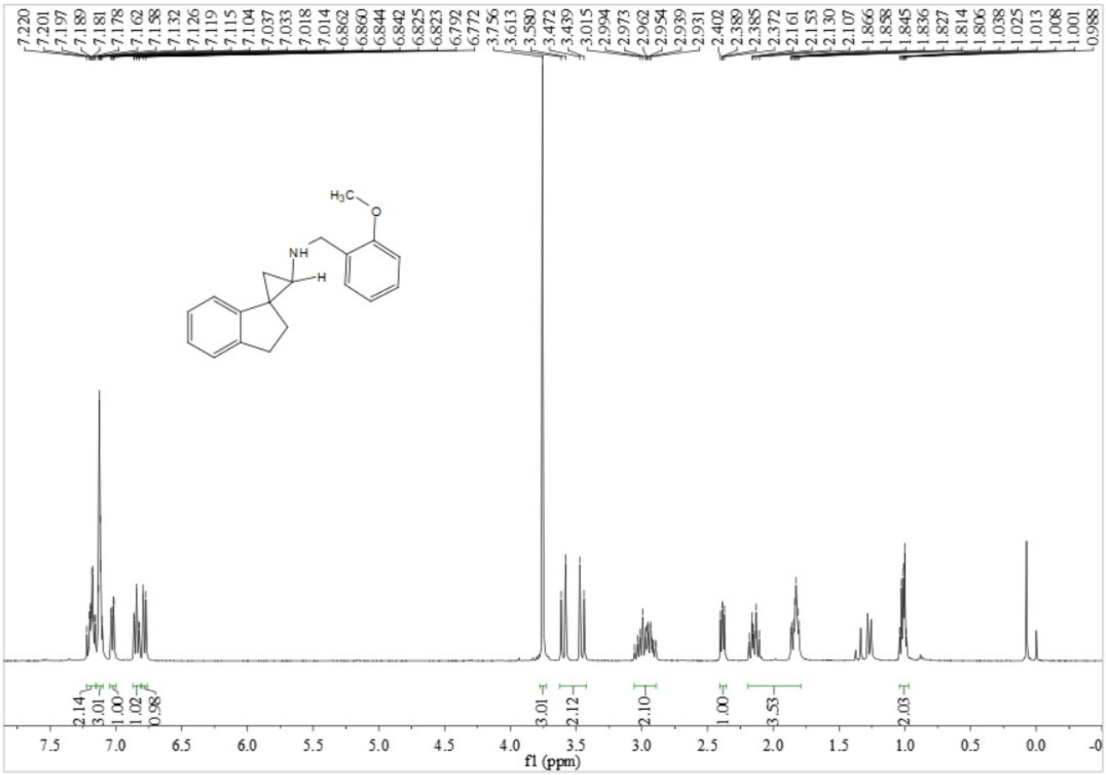

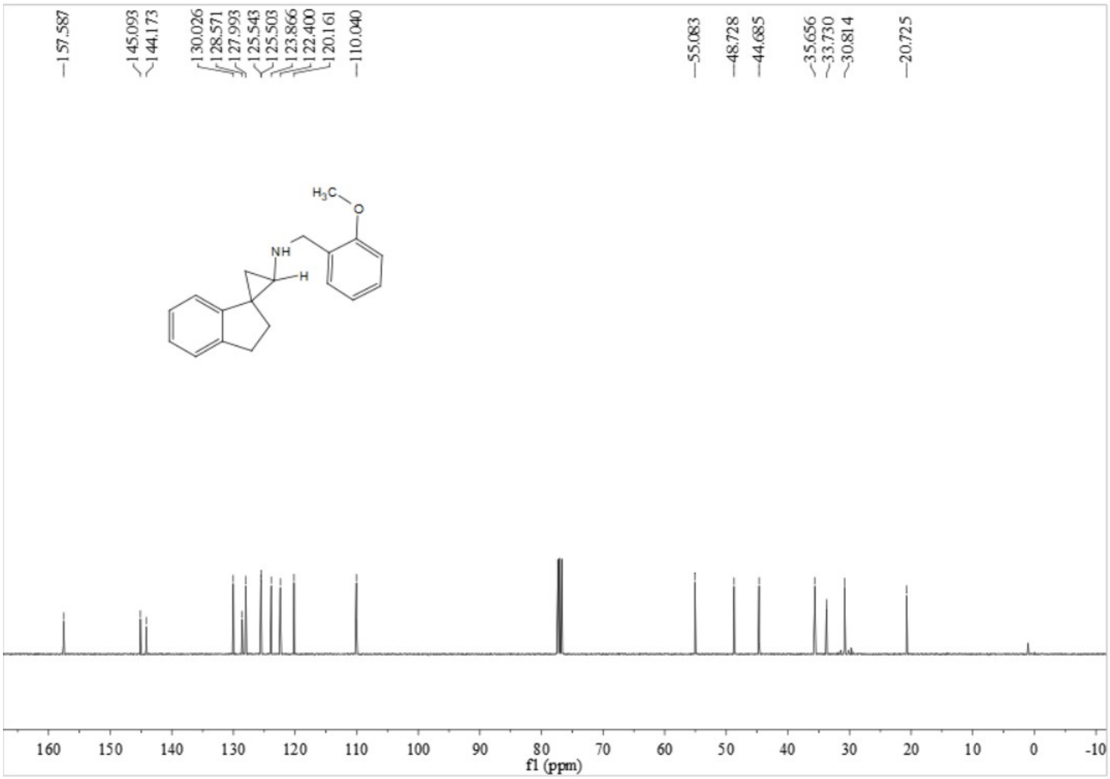

^{1}H NMR (400 MHz, $CDCl_3$) and ^{13}C NMR (101MHz, $CDCl_3$) spectrum for 15a

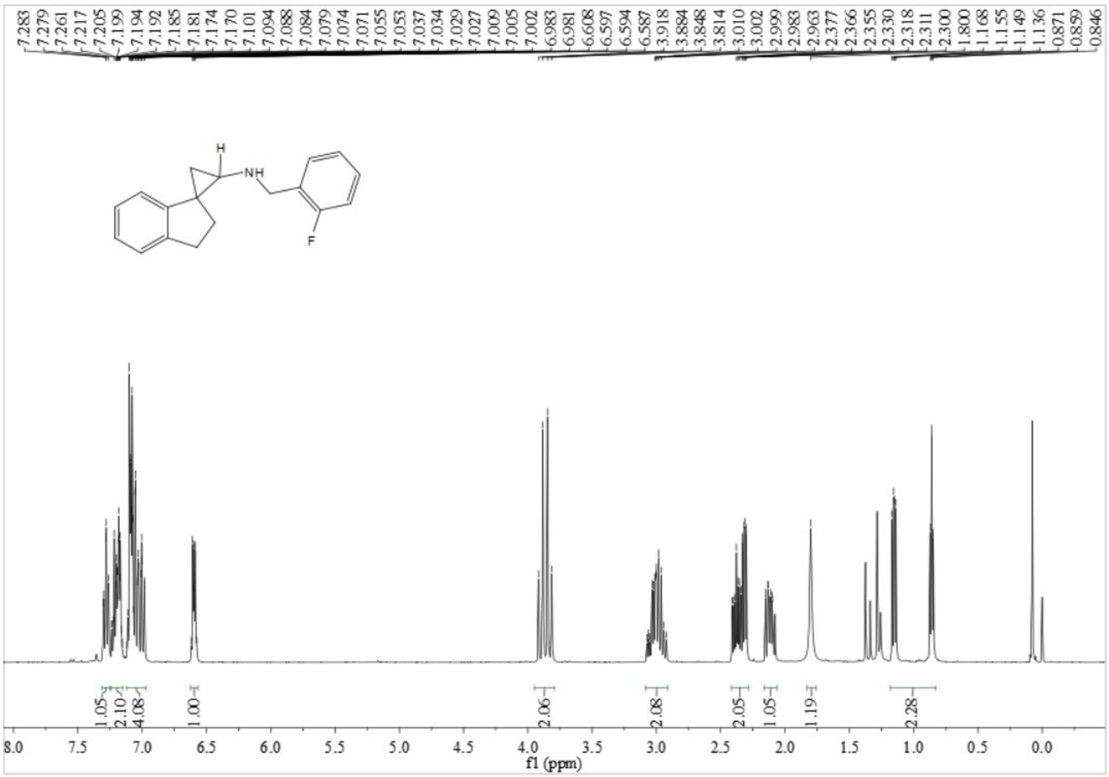

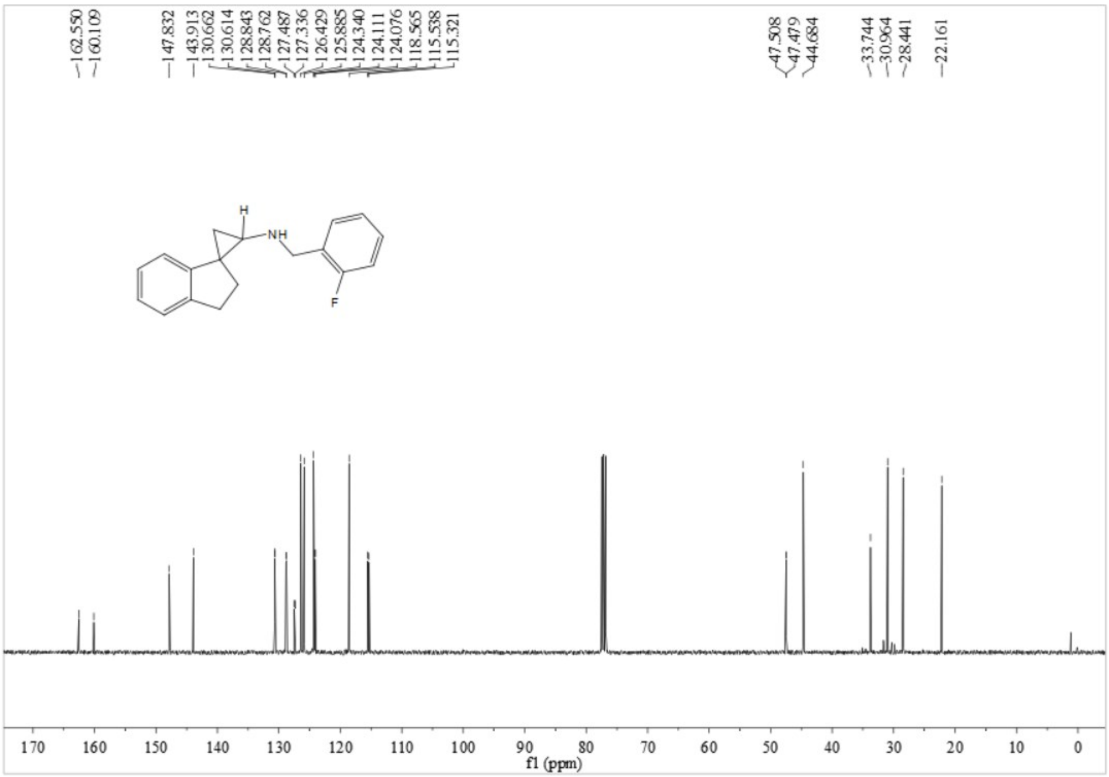

^{1}H NMR (400 MHz, $CDCl_3$) and ^{13}C NMR (101MHz, $CDCl_3$) spectrum for 15b

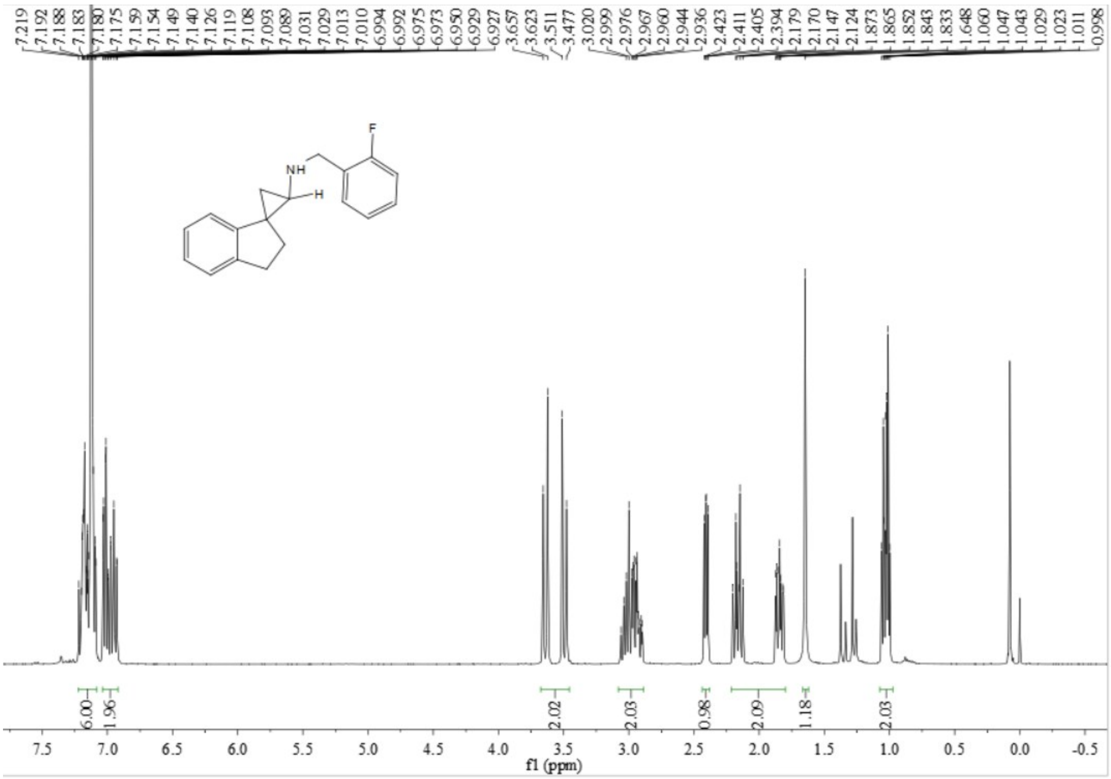

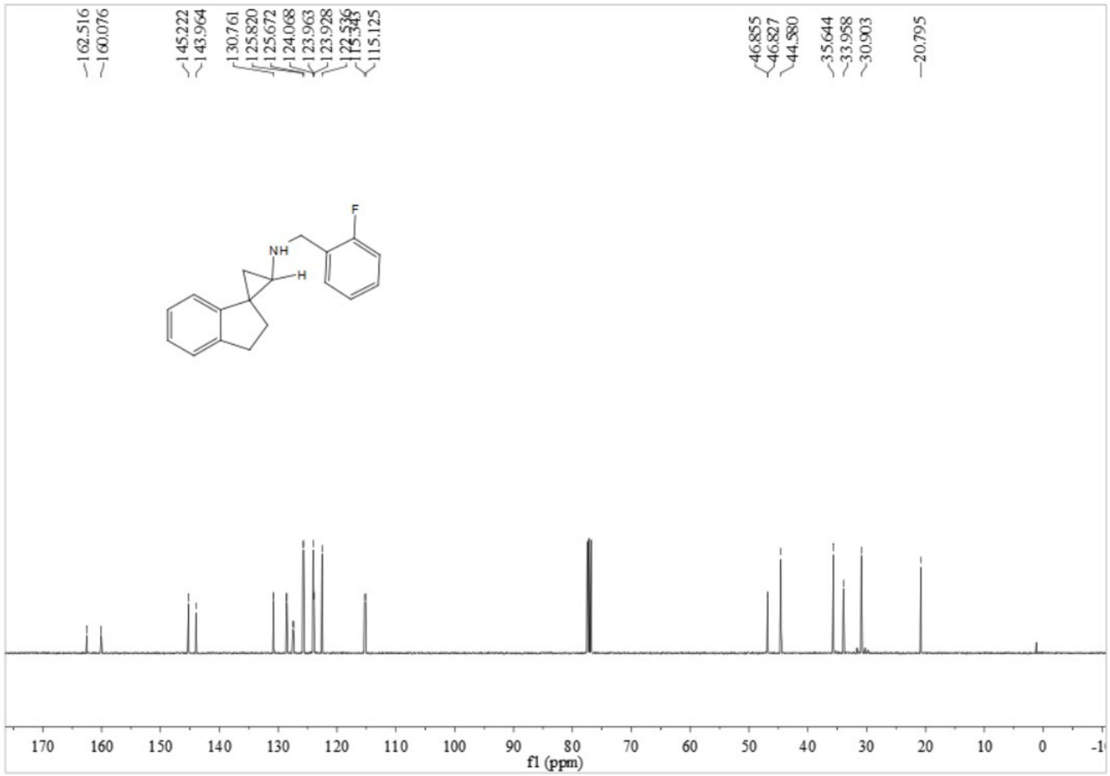

^{1}H NMR (400 MHz, $CDCl_3$) and ^{13}C NMR (101MHz, $CDCl_3$) spectrum for 16a

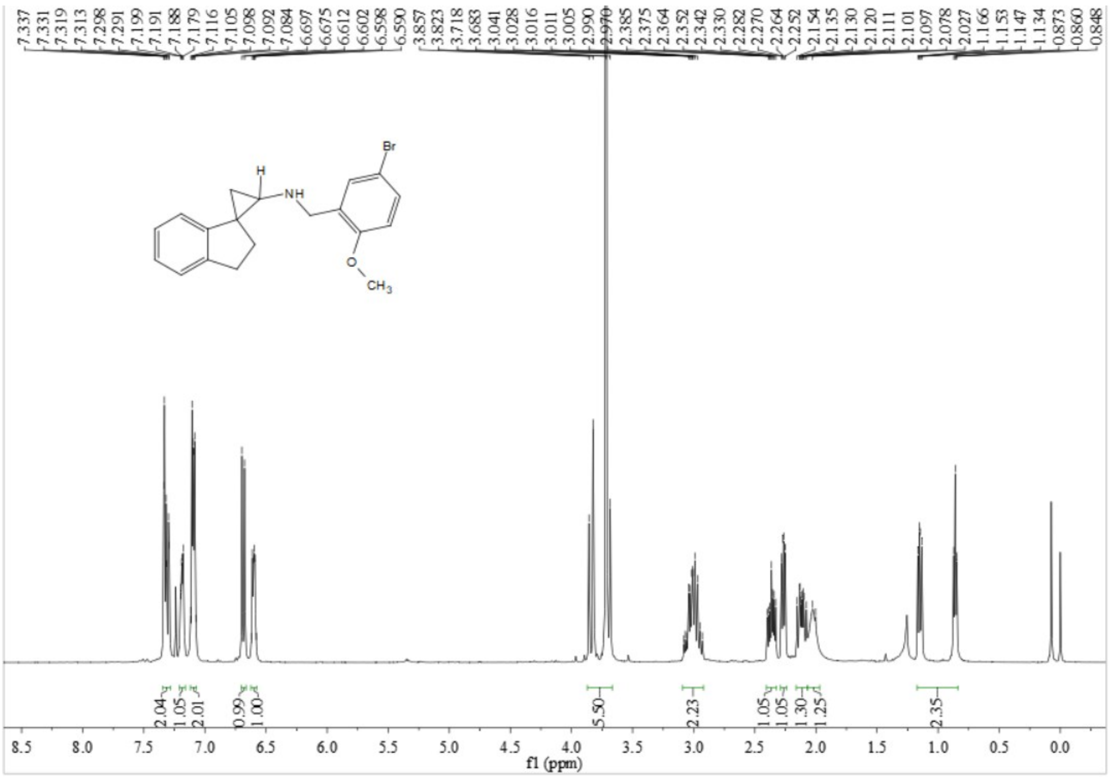

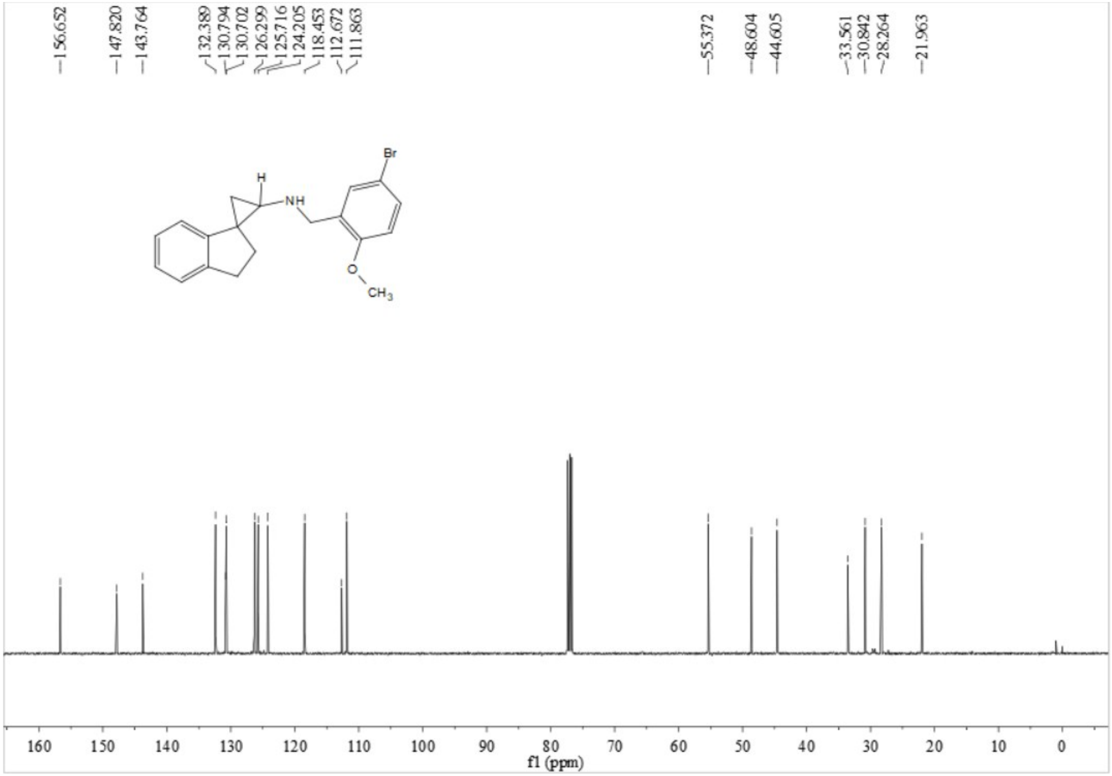

^{1}H NMR (400 MHz, $CDCl_3$) and ^{13}C NMR (101MHz, $CDCl_3$) spectrum for 16b

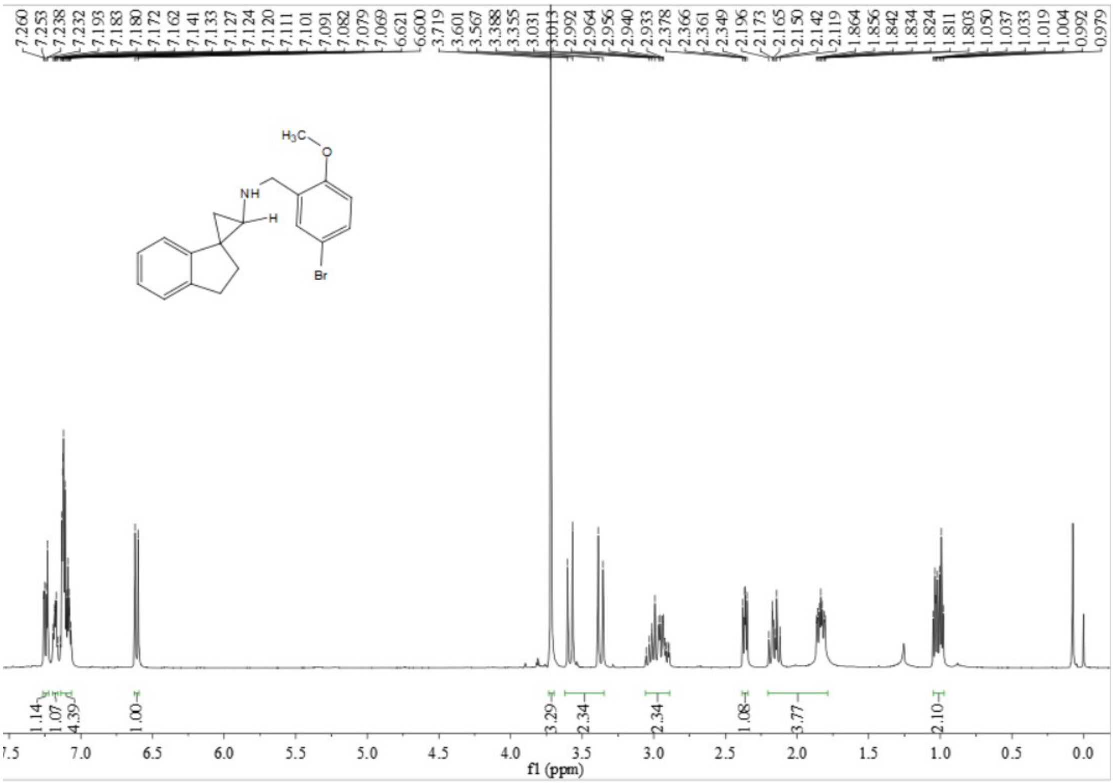

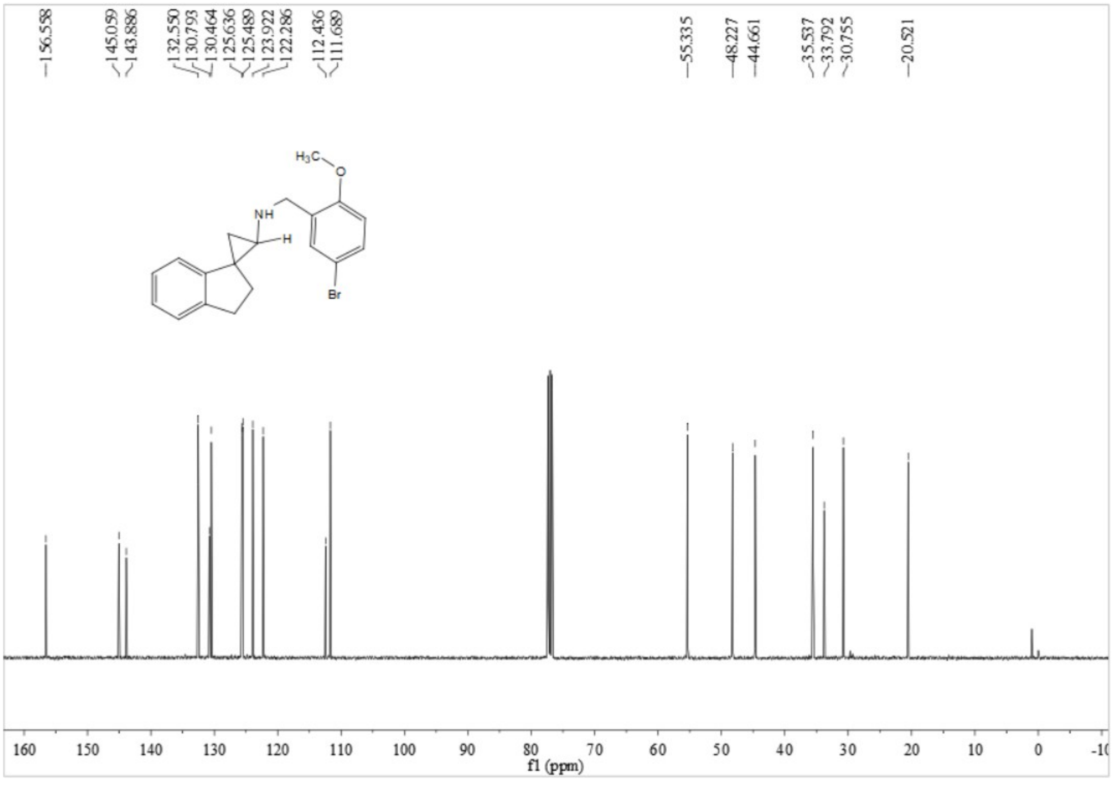

^1H NMR (400 MHz, $CDCl_3$) and ^{13}C NMR (101MHz, $CDCl_3$) spectrum for 17a

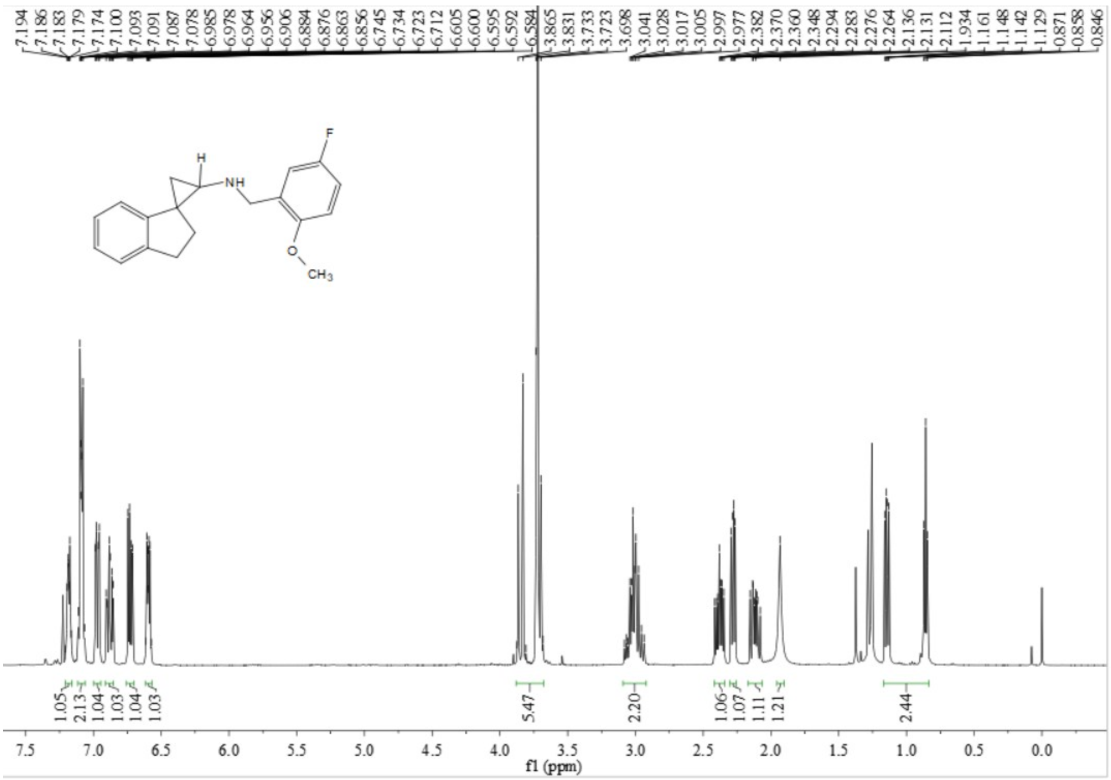

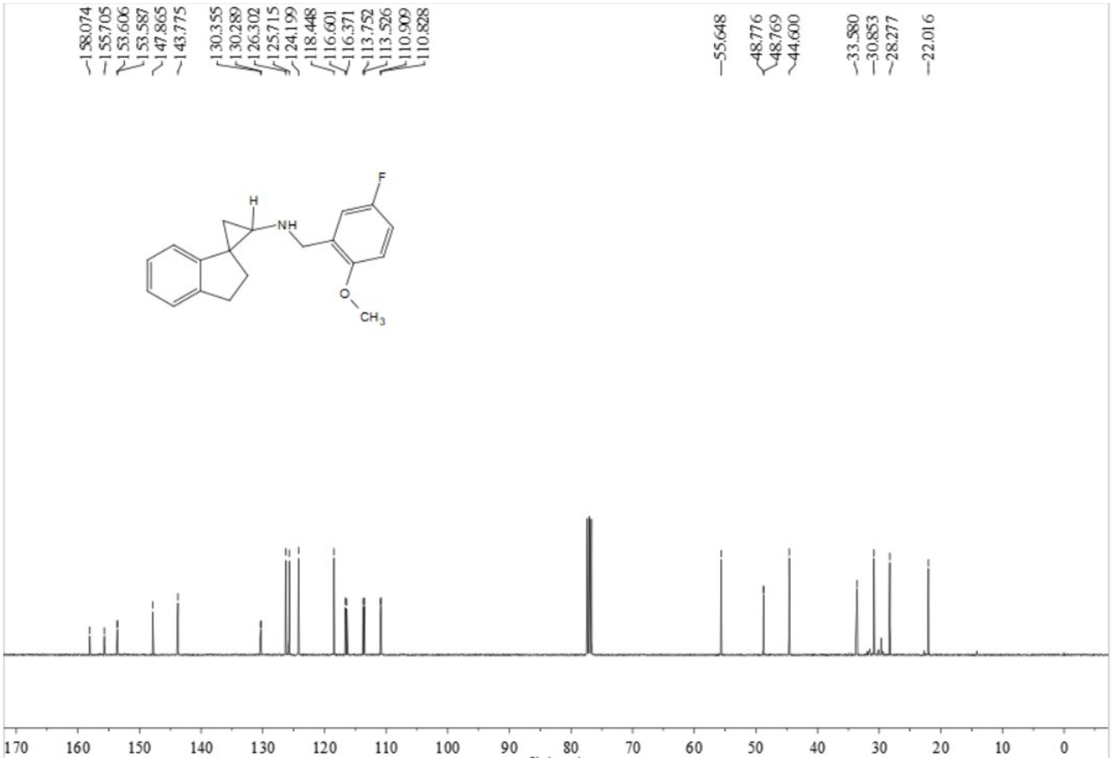

^{1}H NMR (400 MHz, $CDCl_3$) and ^{13}C NMR (101MHz, $CDCl_3$) spectrum for 17b

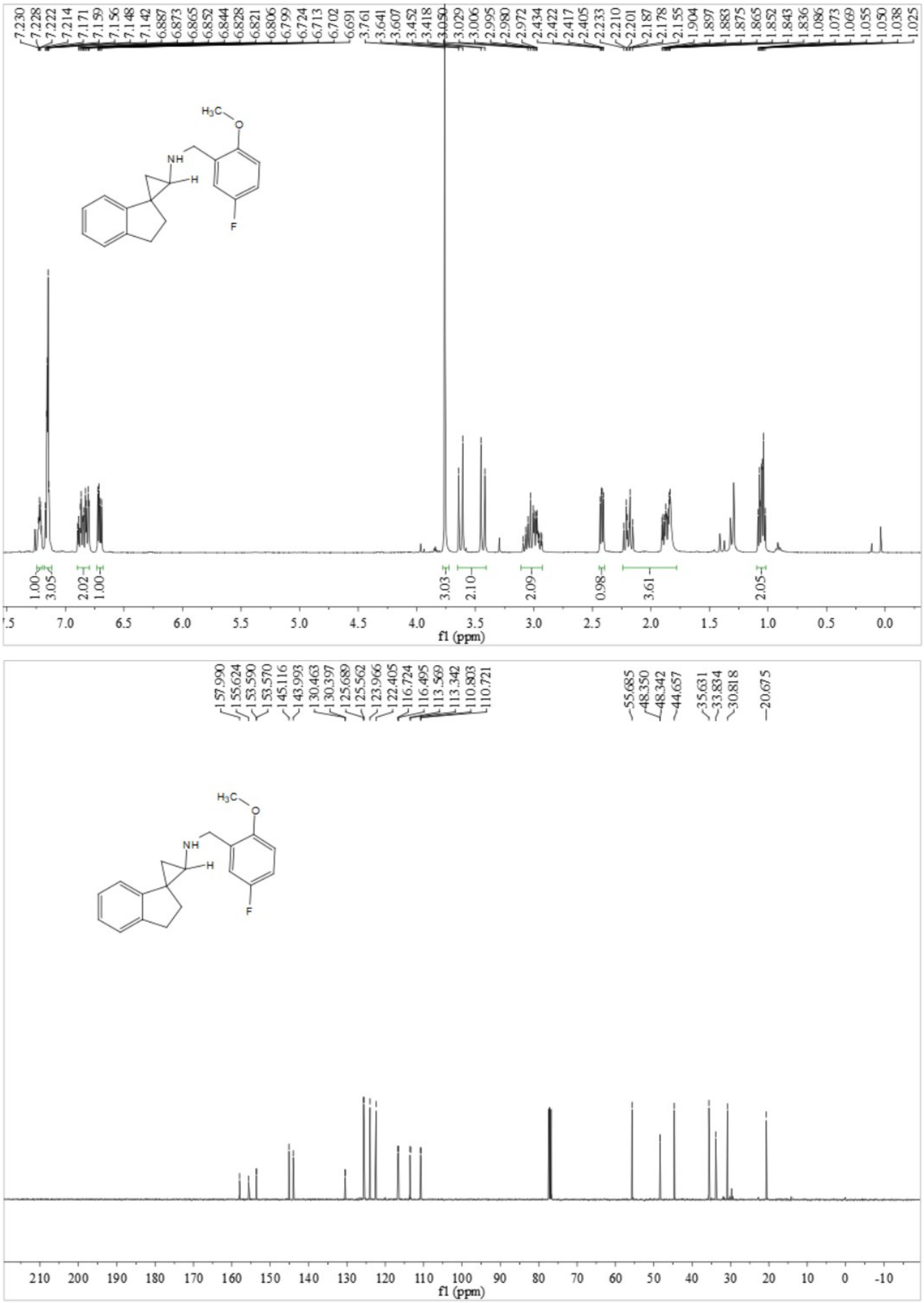

^{1}H NMR (400 MHz, $CDCl_3$) and ^{13}C NMR (101MHz, $CDCl_3$) spectrum for 18a

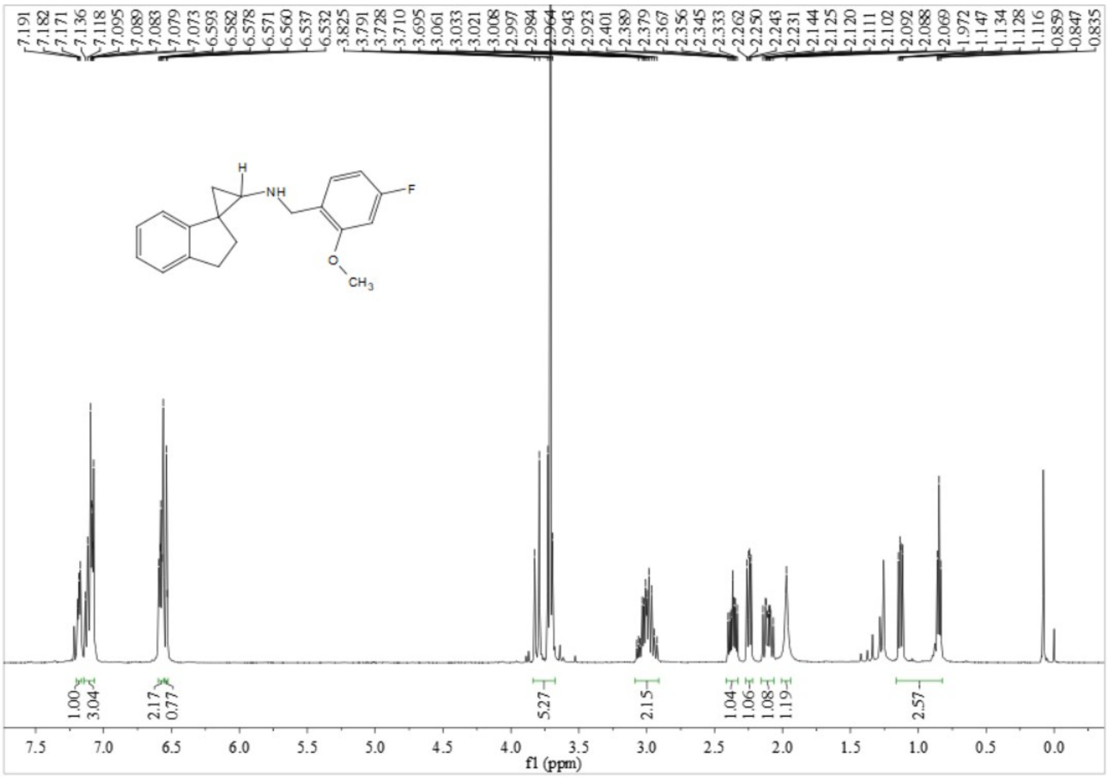

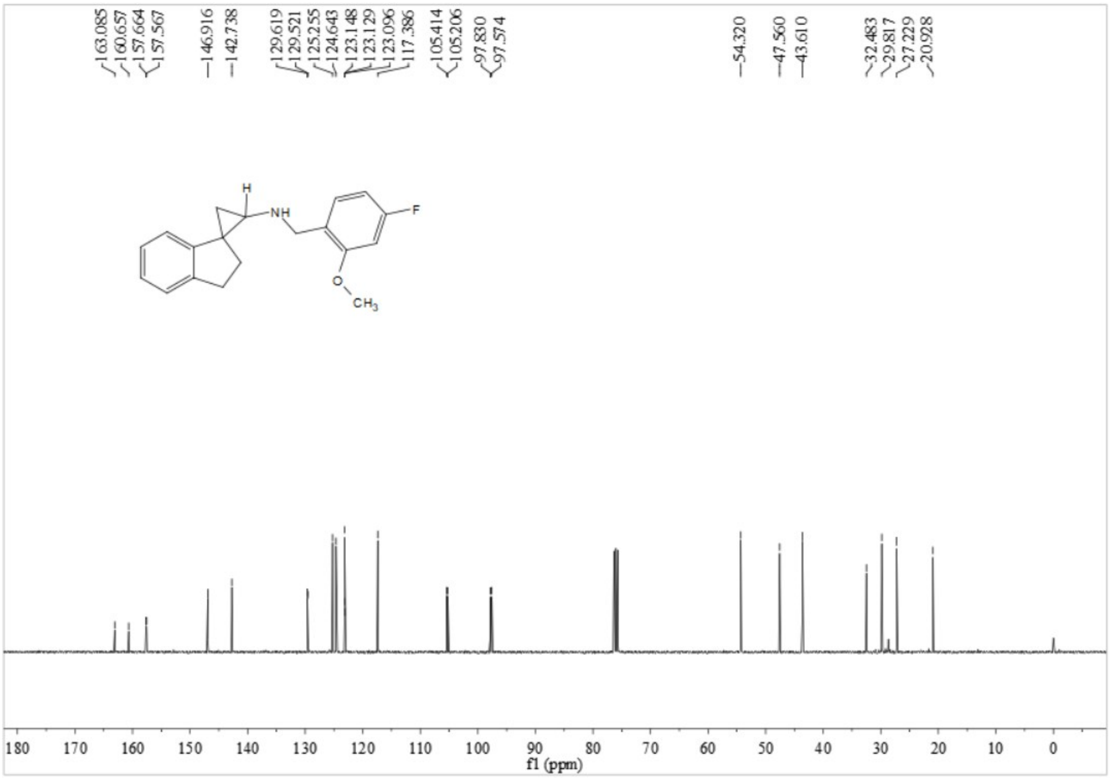

^{1}H NMR (400 MHz, $CDCl_3$) and ^{13}C NMR (101MHz, $CDCl_3$) spectrum for 18b

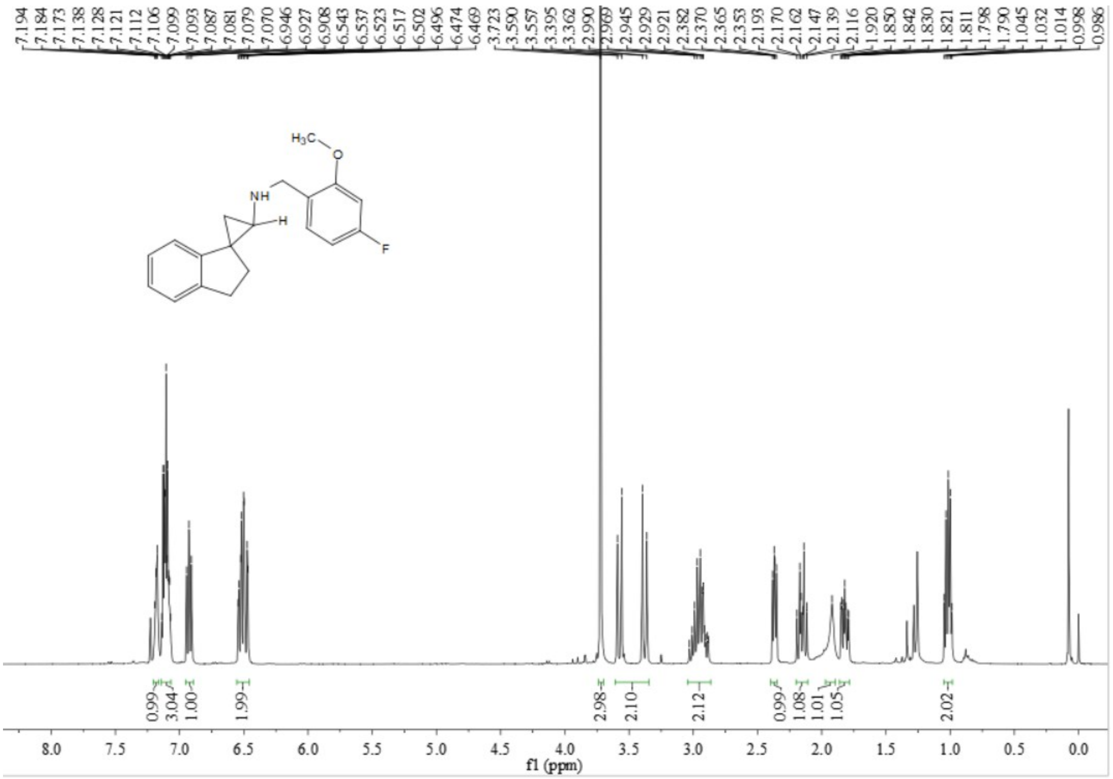

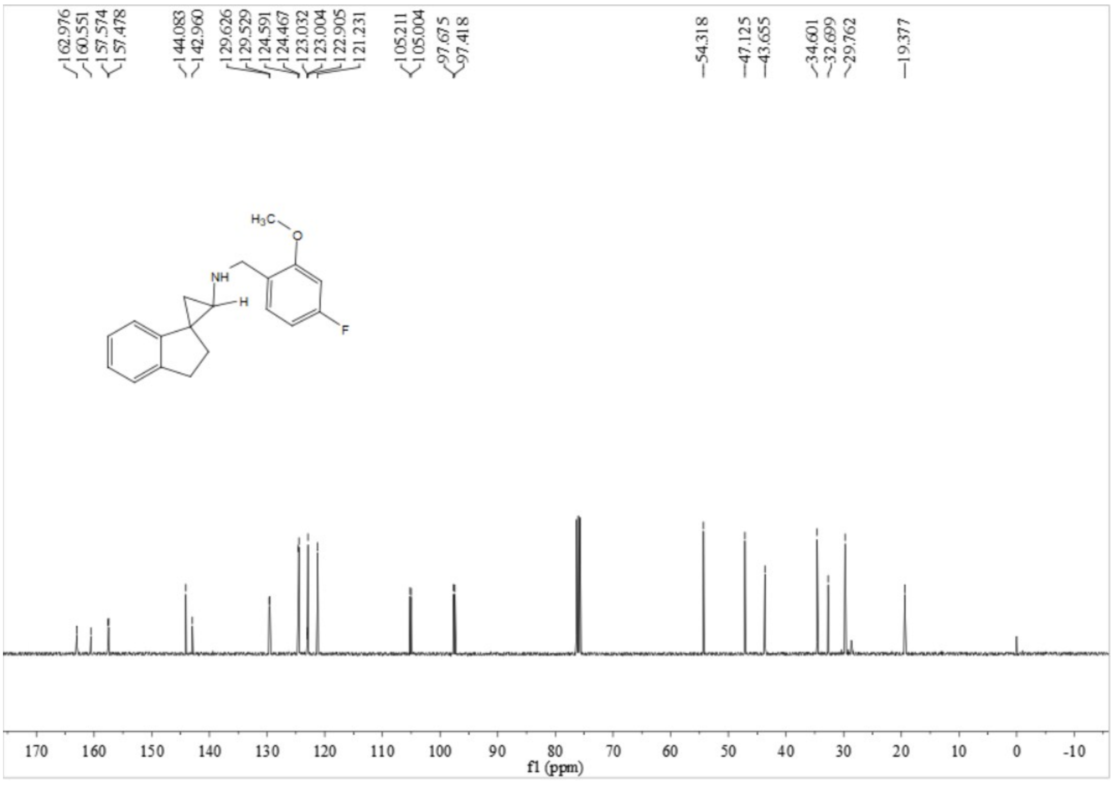

^{1}H NMR (400 MHz, $CDCl_3$) and ^{13}C NMR (101MHz, $CDCl_3$) spectrum for 19a

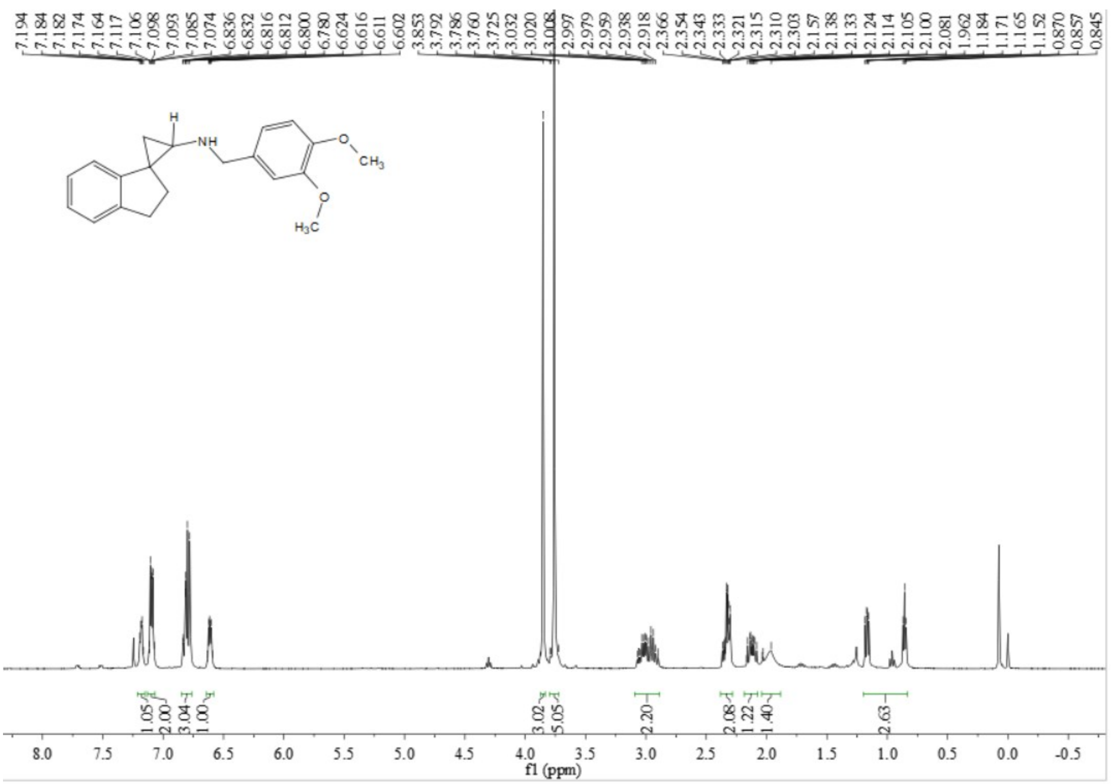

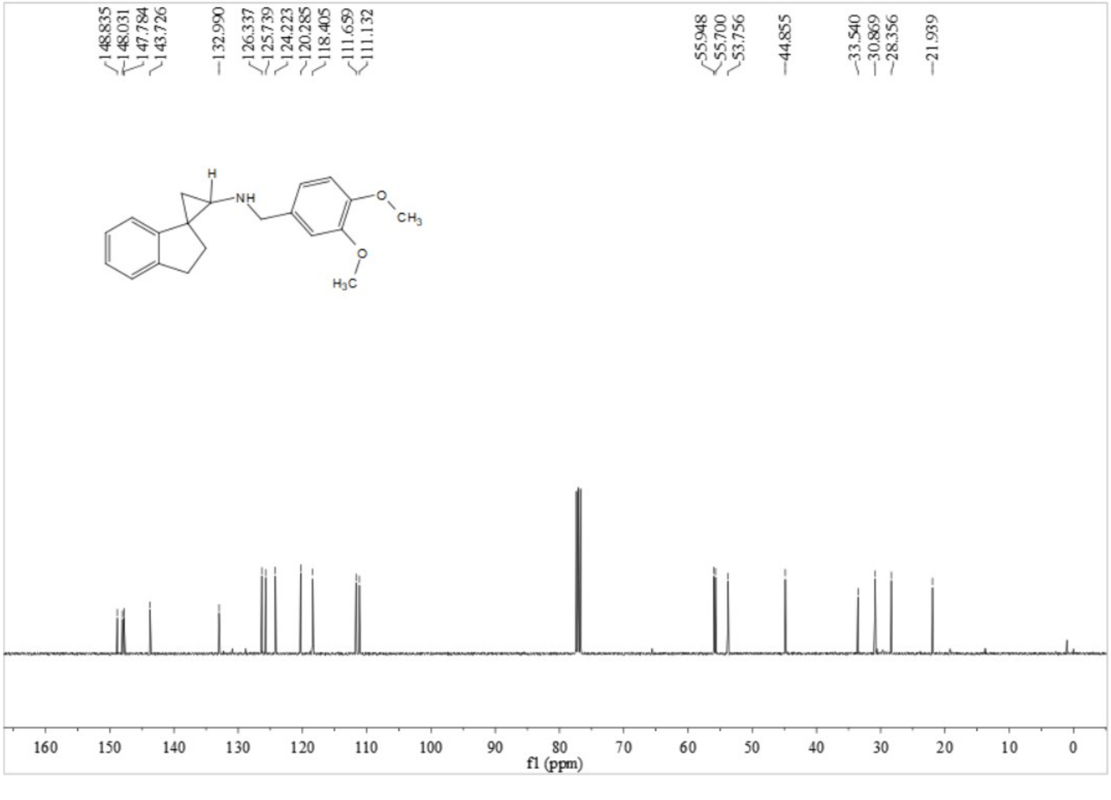

^{1}H NMR (400 MHz, $CDCl_3$) and ^{13}C NMR (101MHz, $CDCl_3$) spectrum for 19b

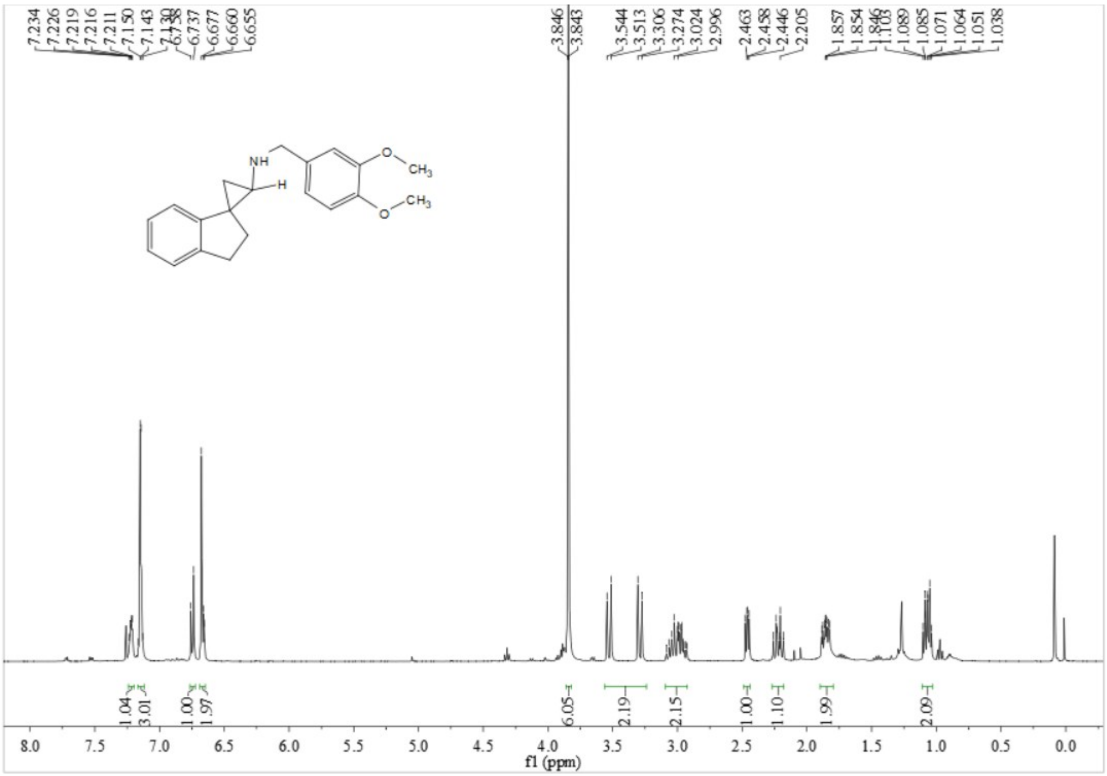

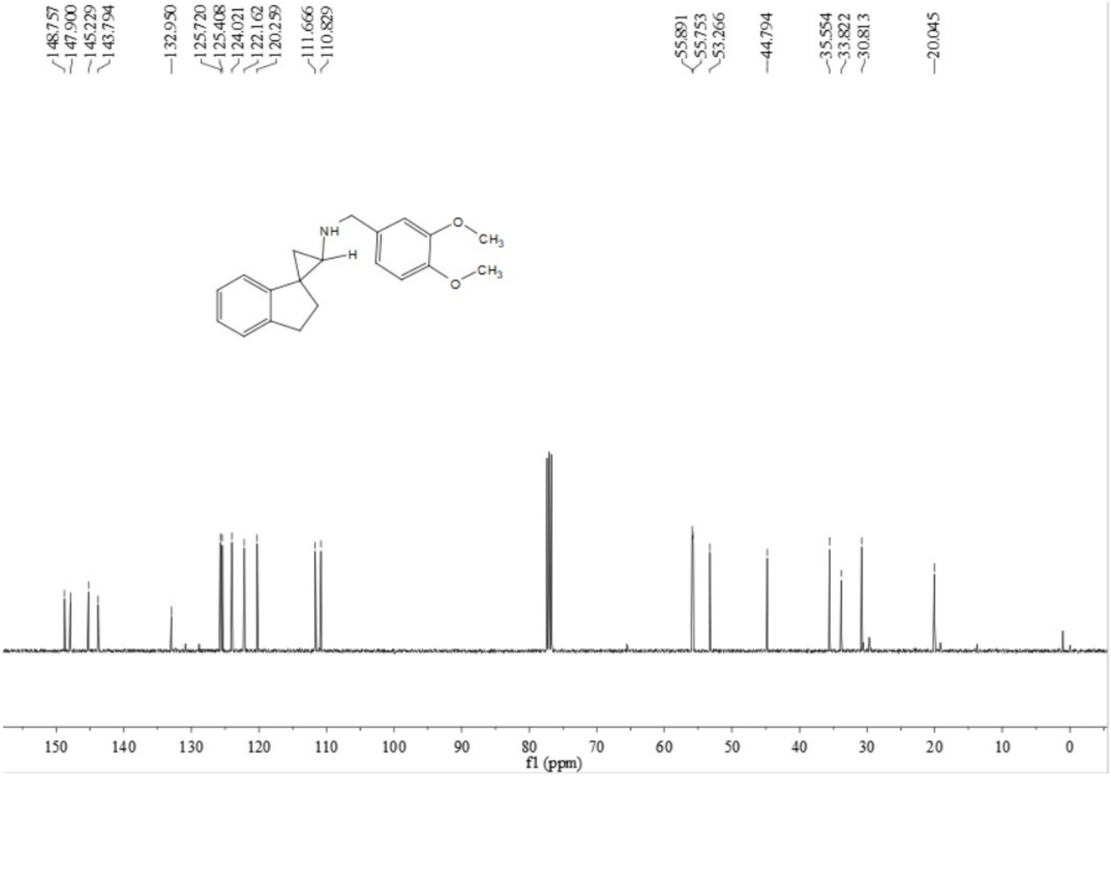

^{1}H NMR (400 MHz, $CDCl_3$) and ^{13}C NMR (101MHz, $CDCl_3$) spectrum for 20a

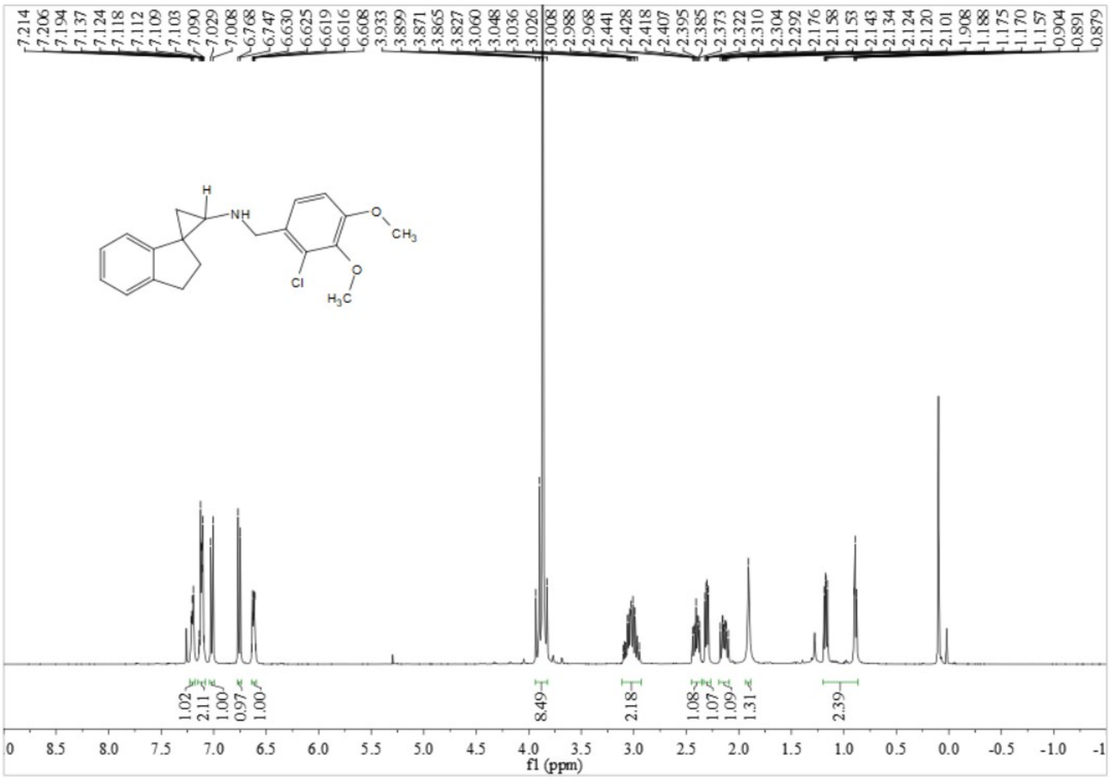

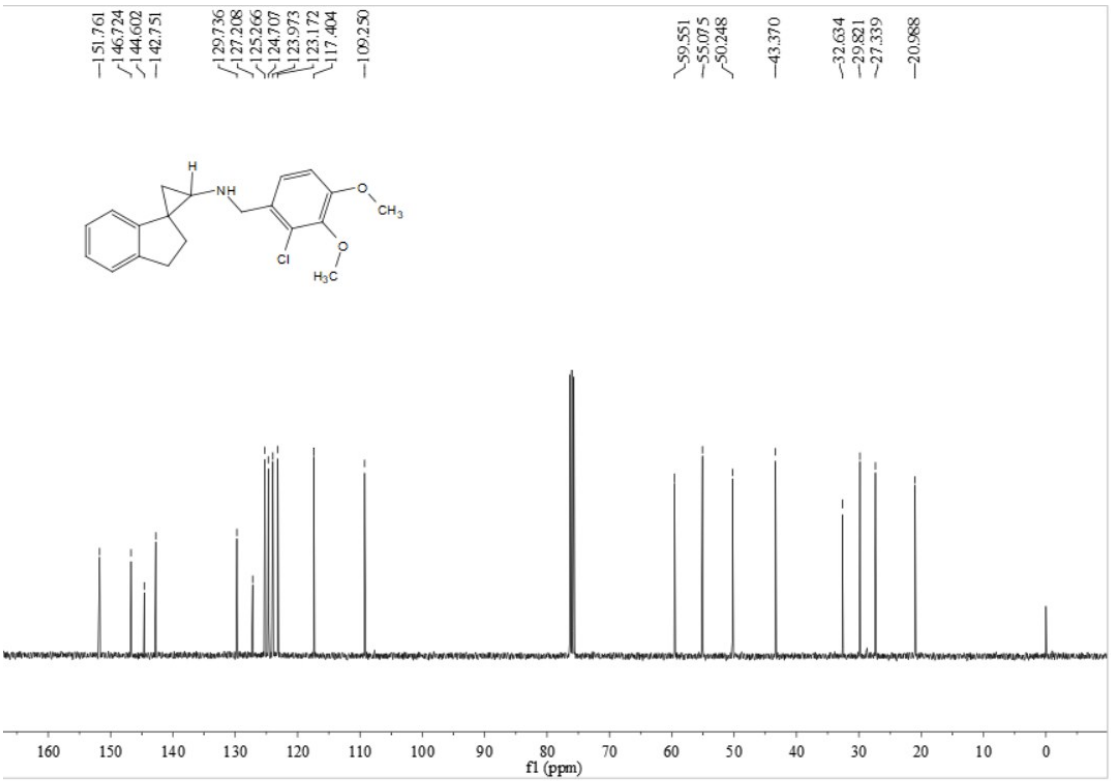

^{1}H NMR (400 MHz, $CDCl_3$) and ^{13}C NMR (101MHz, $CDCl_3$) spectrum for 20b

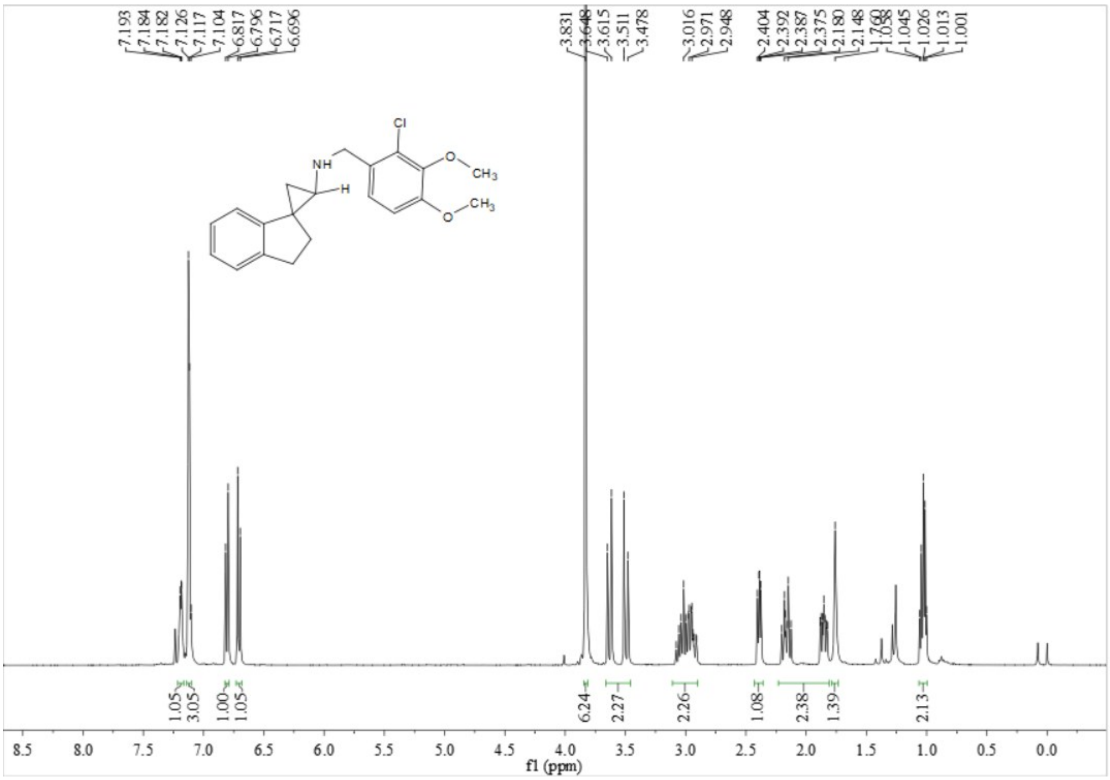

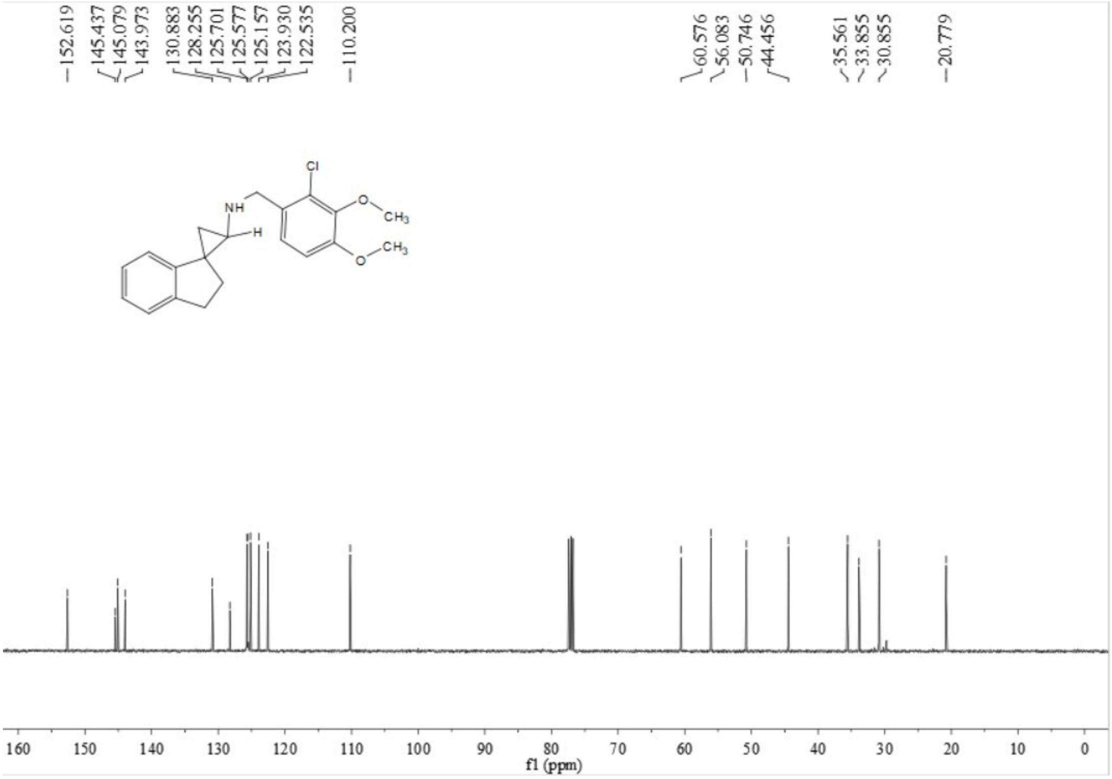

^{1}H NMR (400 MHz, CD_3OD) and ^{13}C NMR (101MHz, CD_3OD) spectrum for 21a

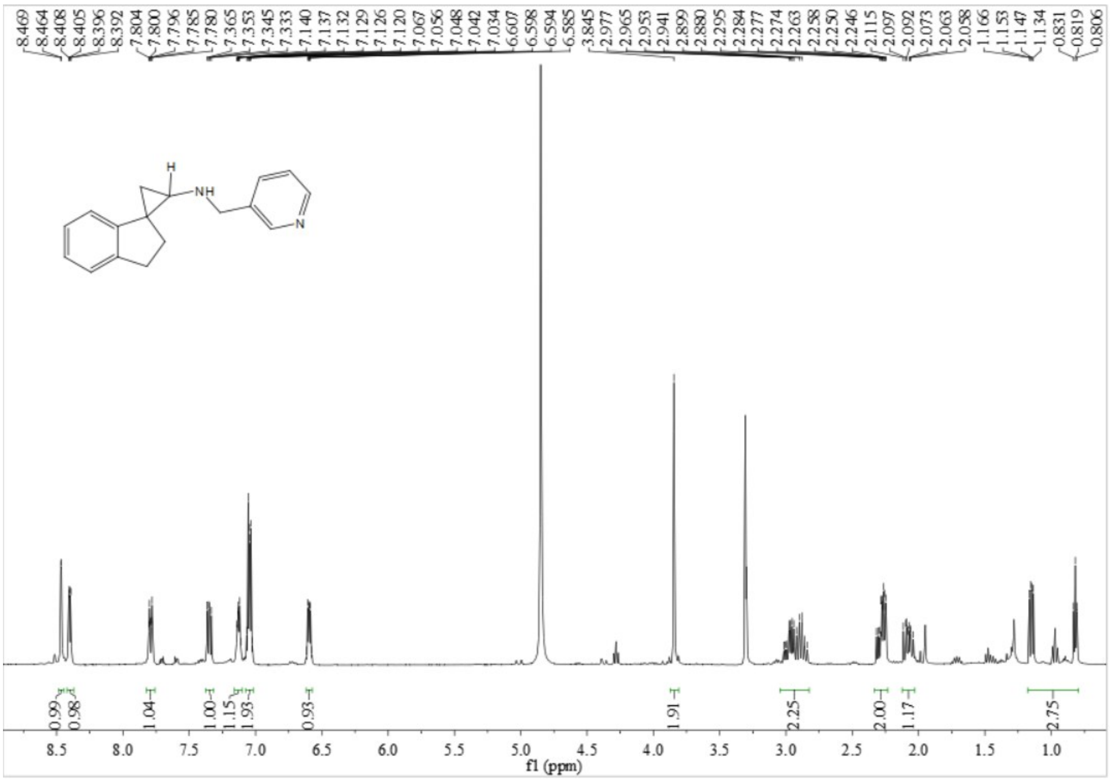

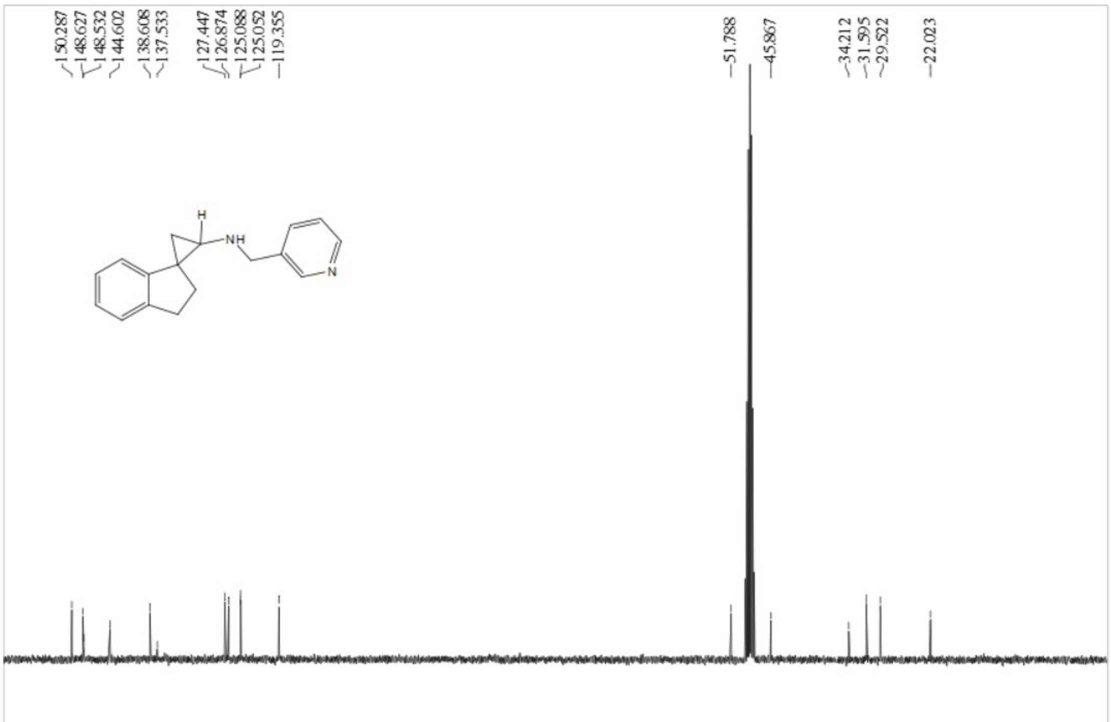

^{1}H NMR (400 MHz, CD_3OD) and ^{13}C NMR (101MHz, CD_3OD) spectrum for 21b

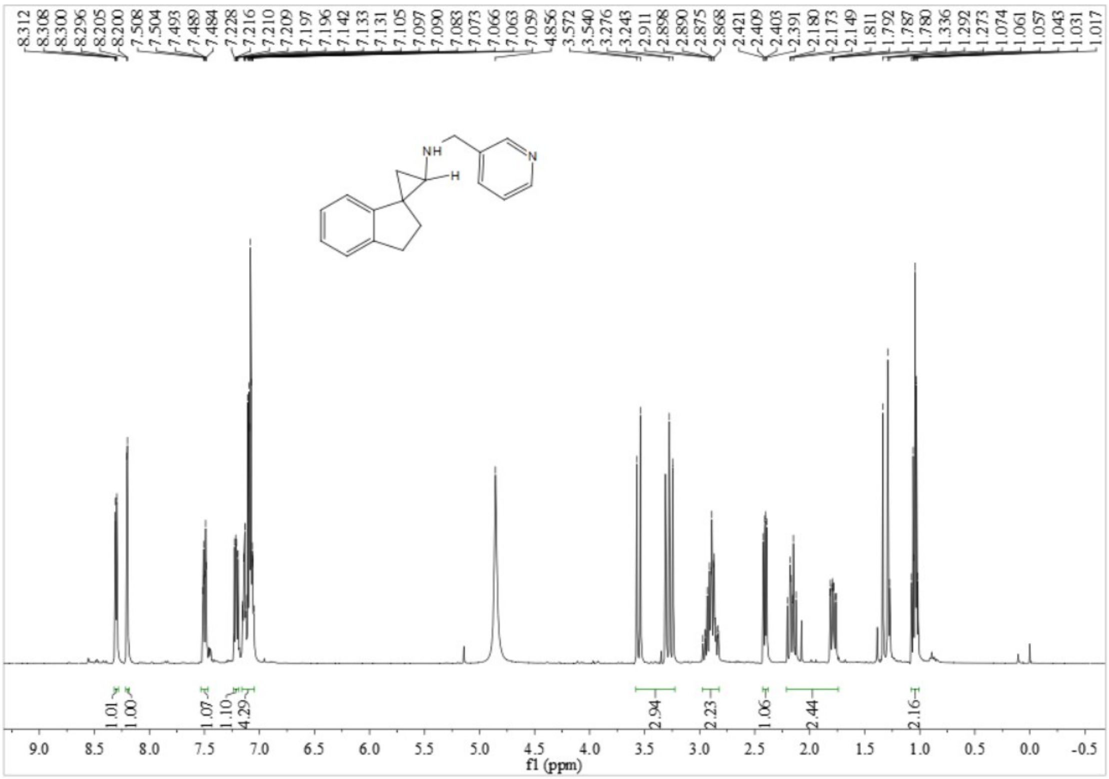

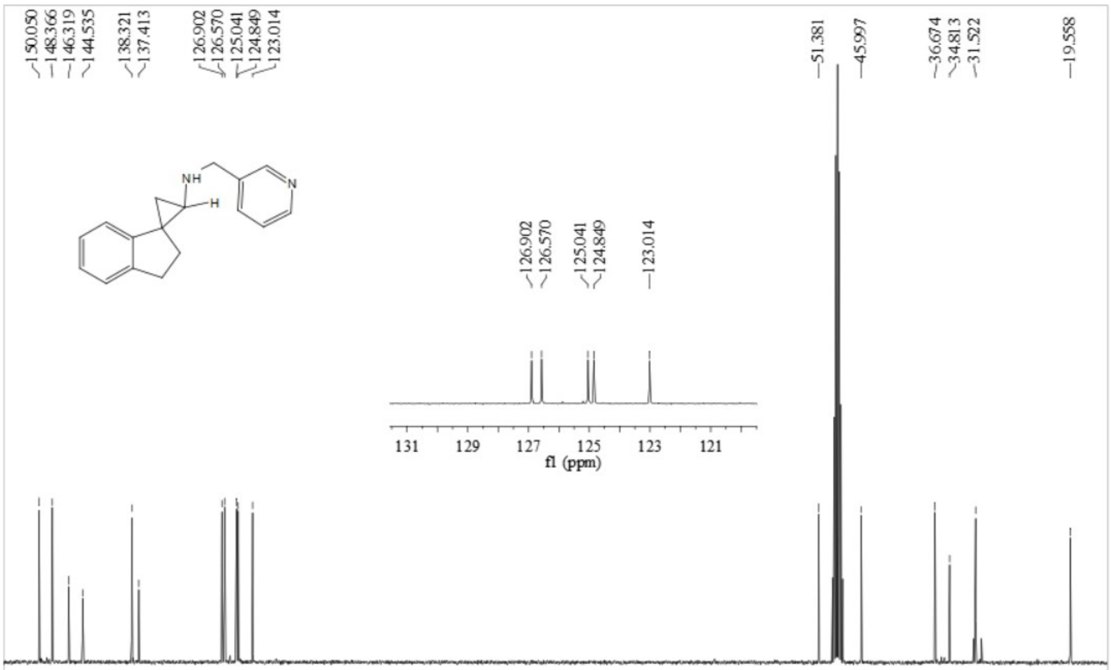

^{1}H NMR (400 MHz, $CDCl_3$) and ^{13}C NMR (101MHz, $CDCl_3$) spectrum for 22a

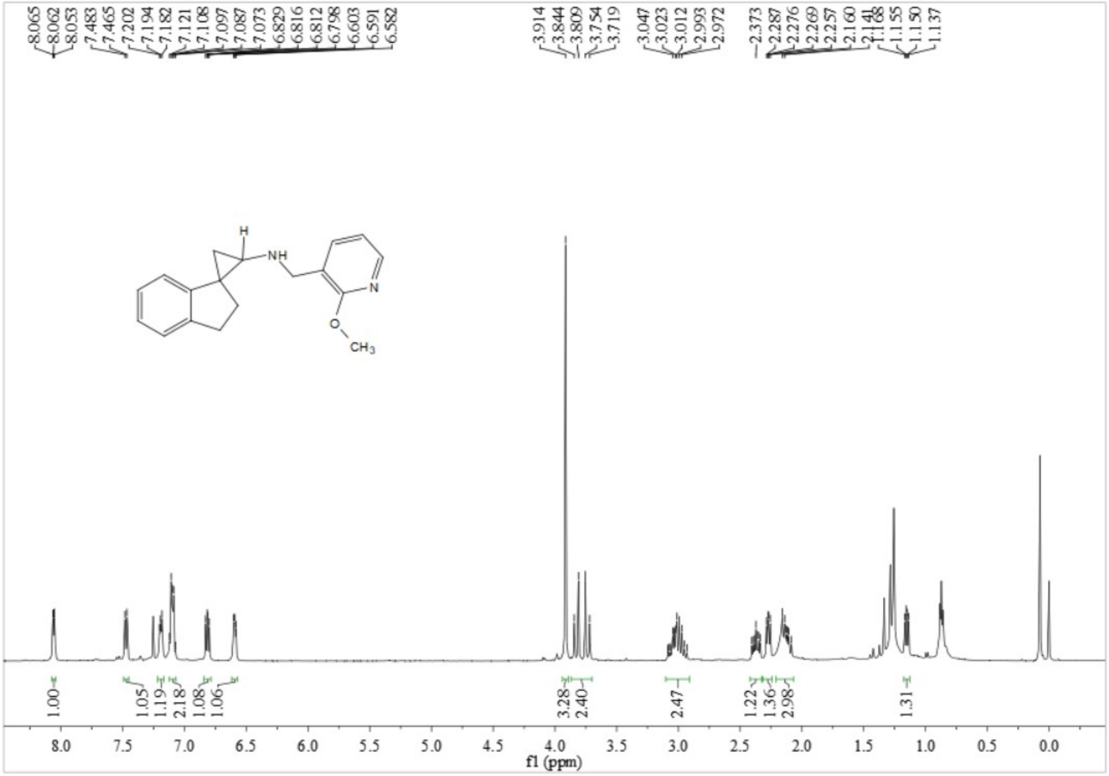

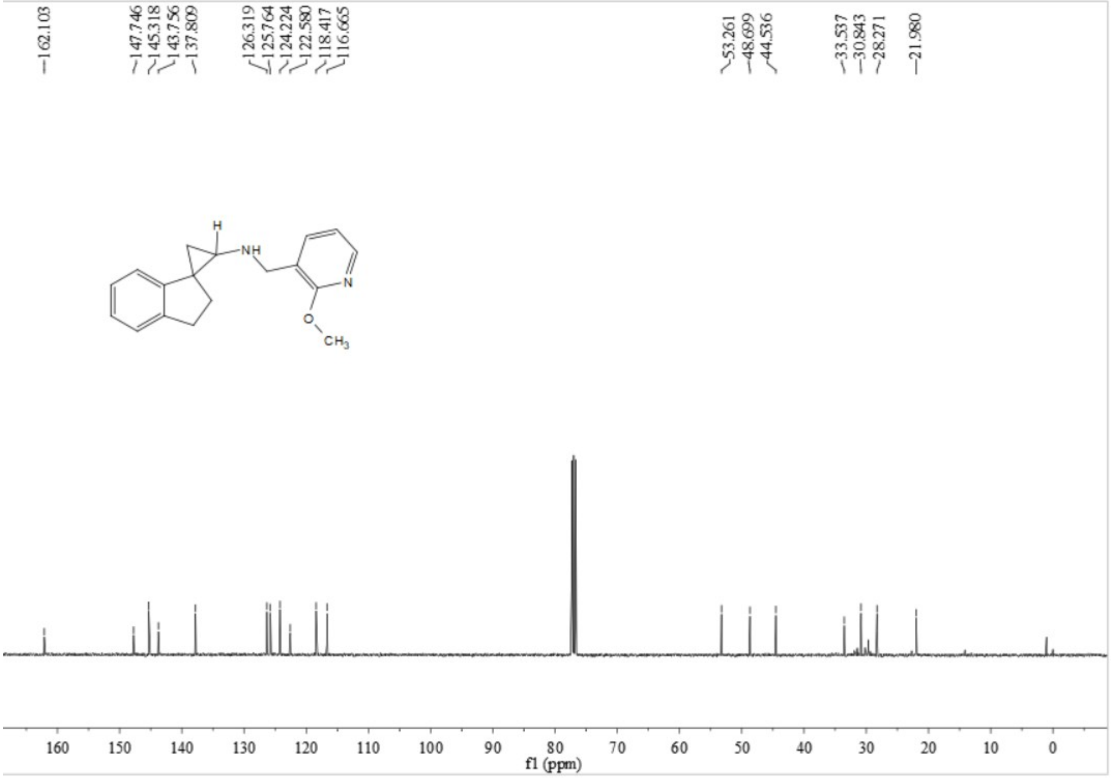

^{1}H NMR (400 MHz, CD_3OD) and ^{13}C NMR (101MHz, CD_3OD) spectrum for 22b

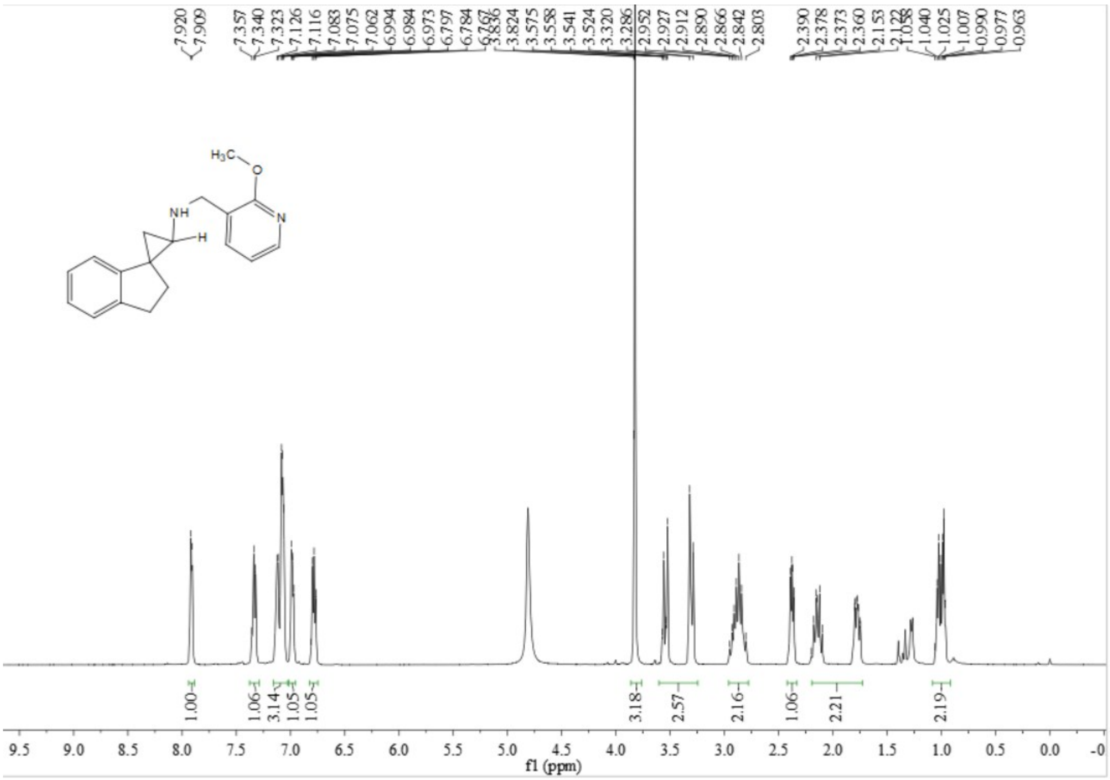

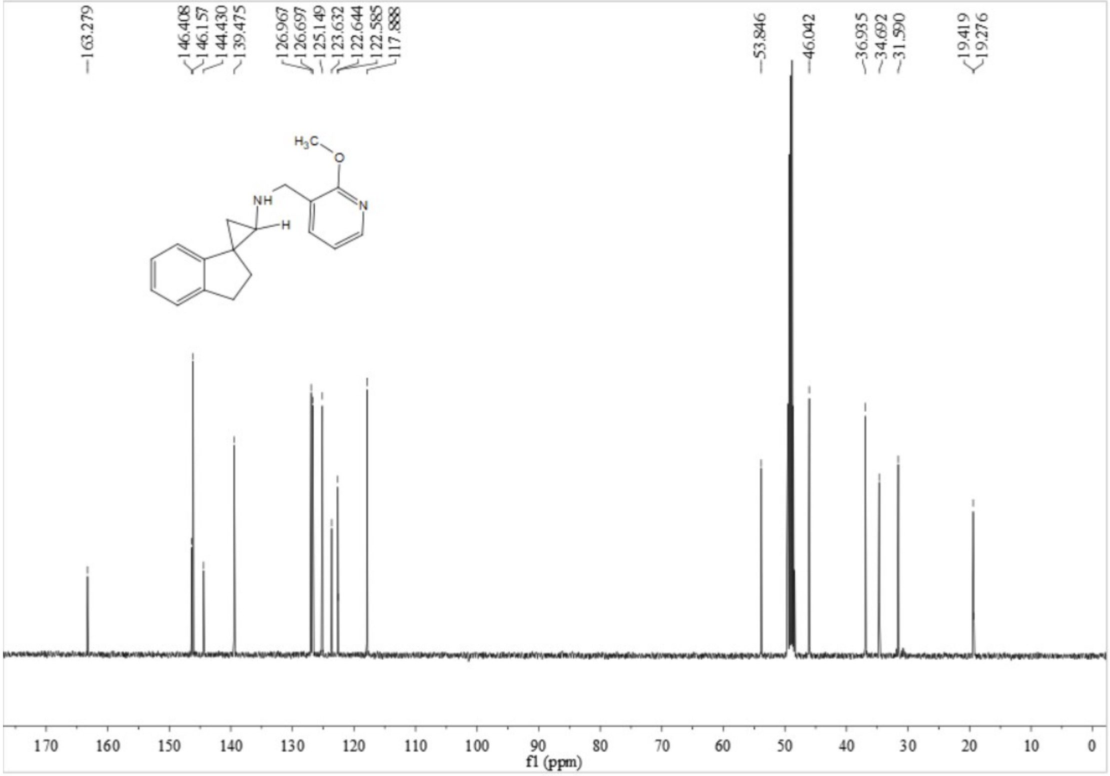

^{1}H NMR (400 MHz, $CDCl_3$) and ^{13}C NMR (101MHz, $CDCl_3$) spectrum for 23a

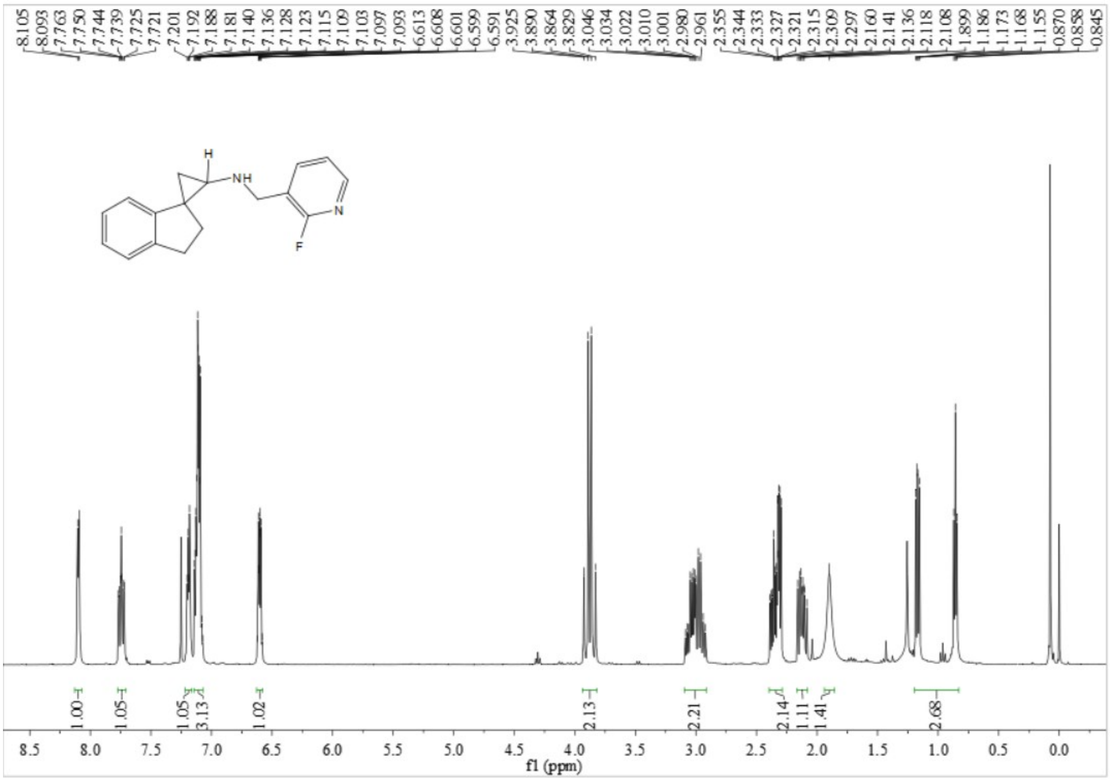

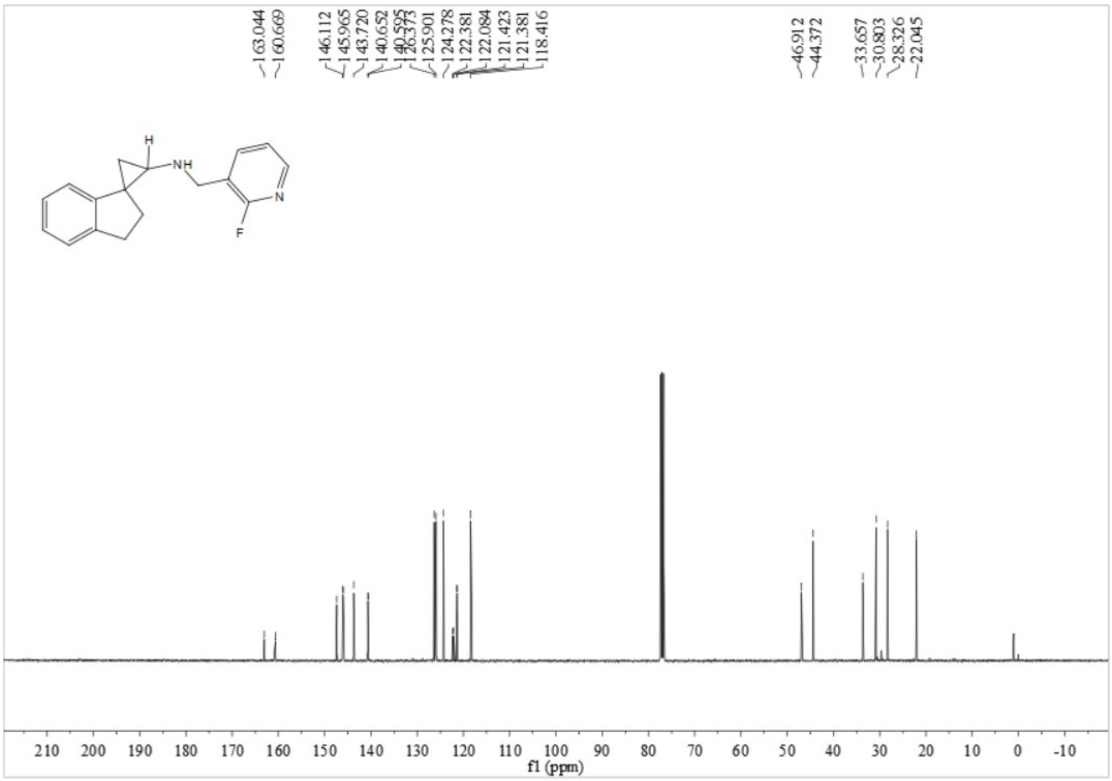

^{1}H NMR (400 MHz, $CDCl_3$) and ^{13}C NMR (101MHz, $CDCl_3$) spectrum for 23b

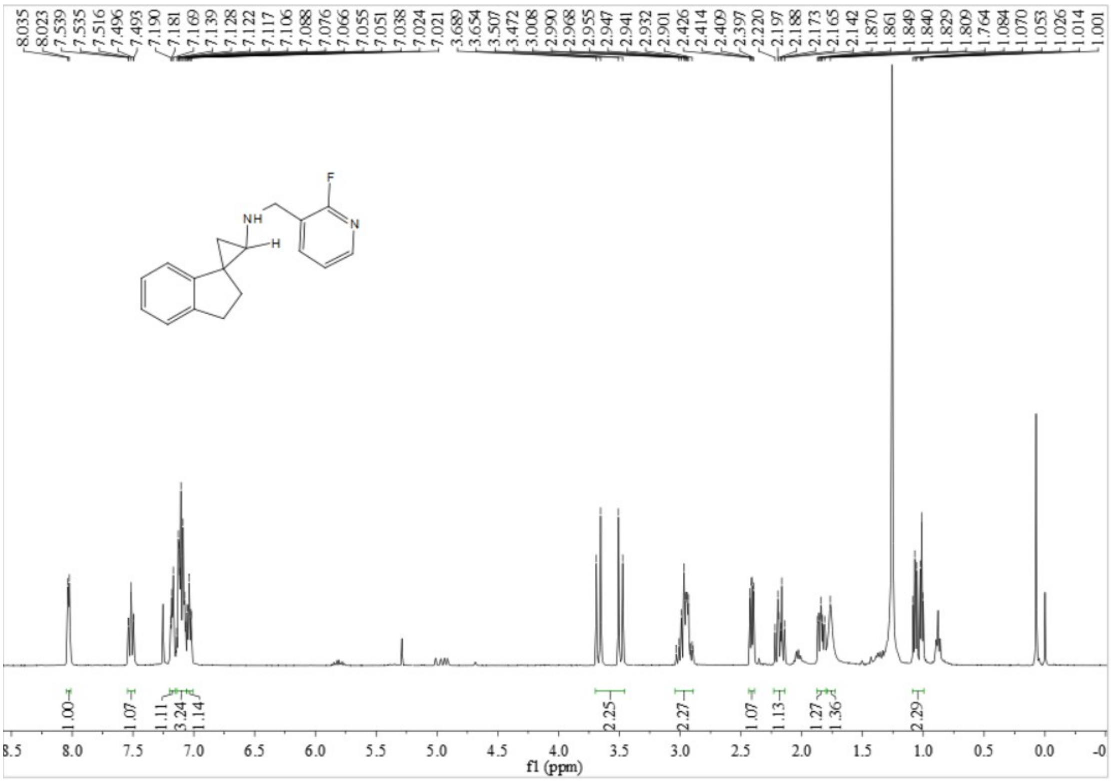

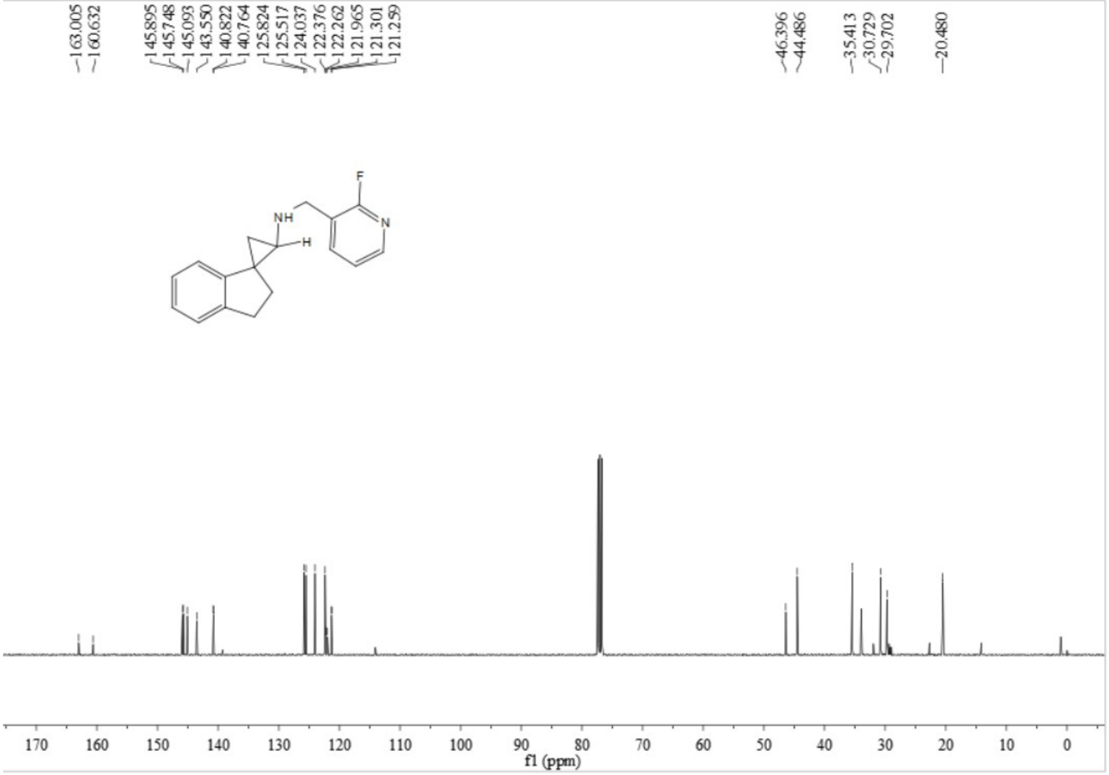

^{1}H NMR (400 MHz, $CDCl_3$) and ^{13}C NMR (101MHz, $CDCl_3$) spectrum for 24a

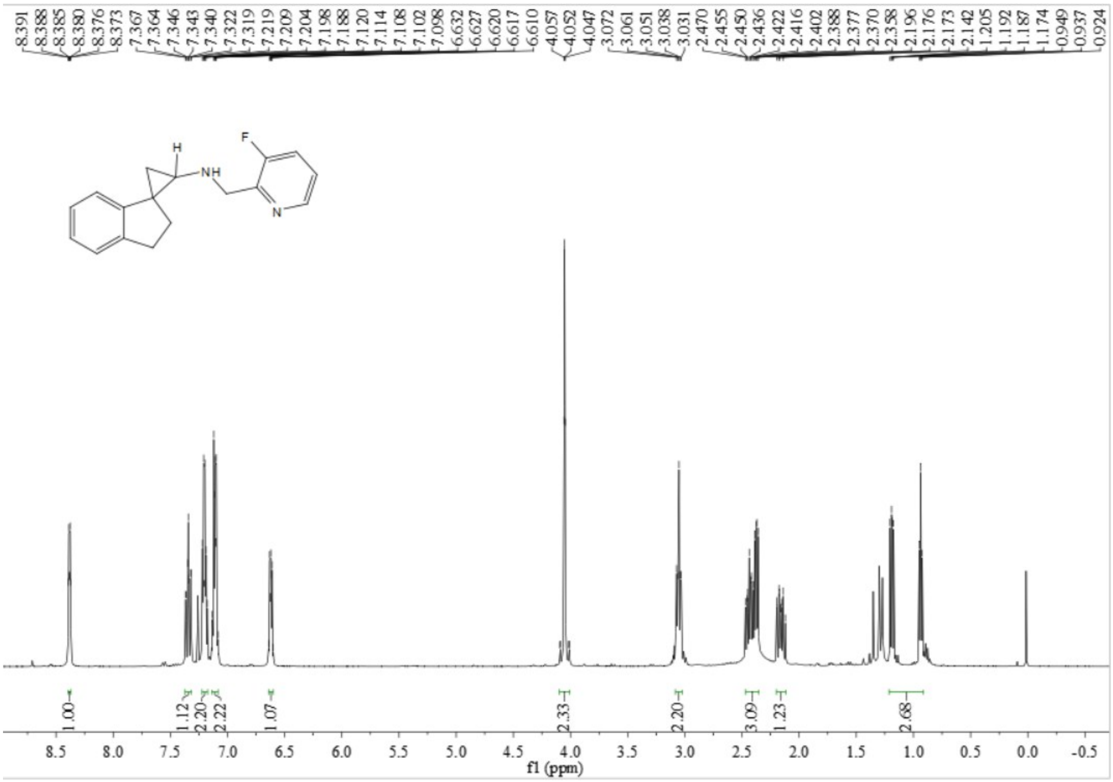

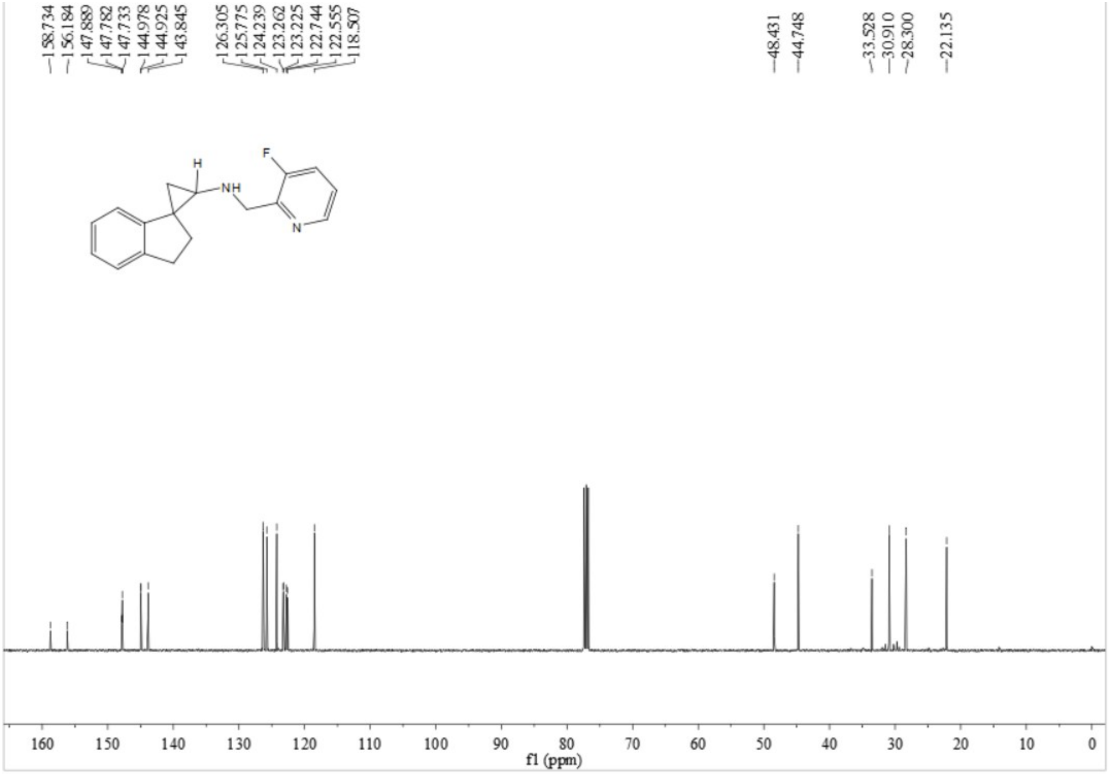

^{1}H NMR (400 MHz, $CDCl_3$) and ^{13}C NMR (101MHz, $CDCl_3$) spectrum for 24b

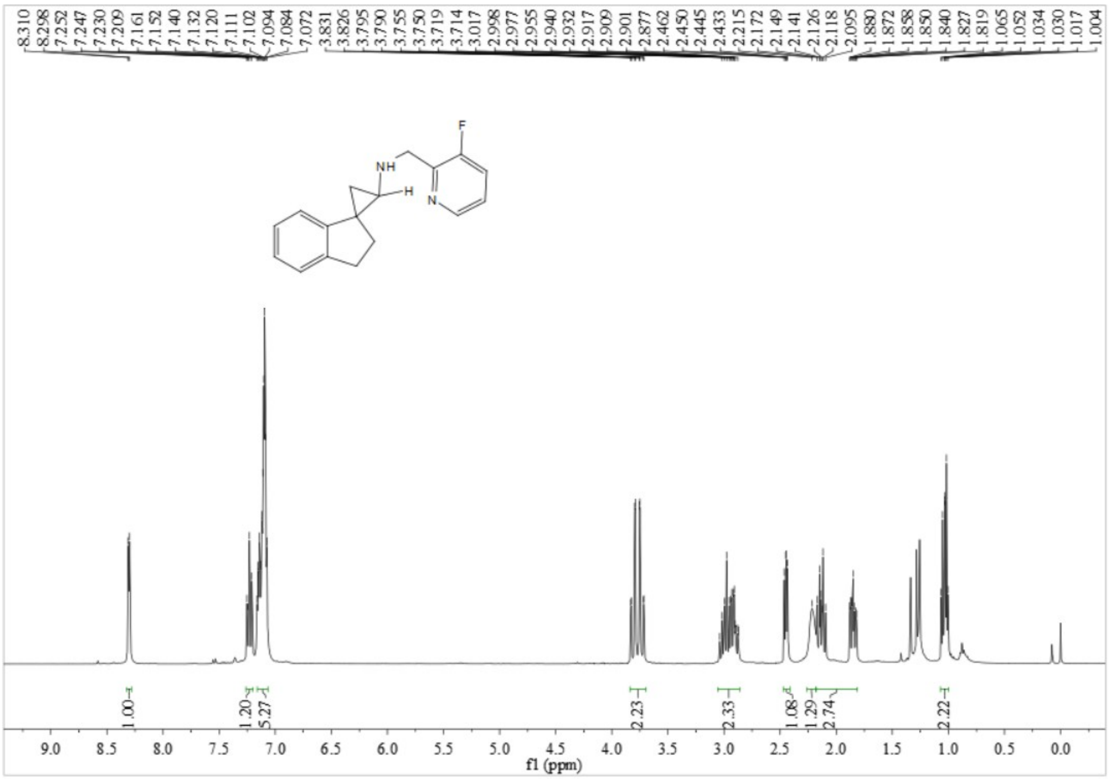

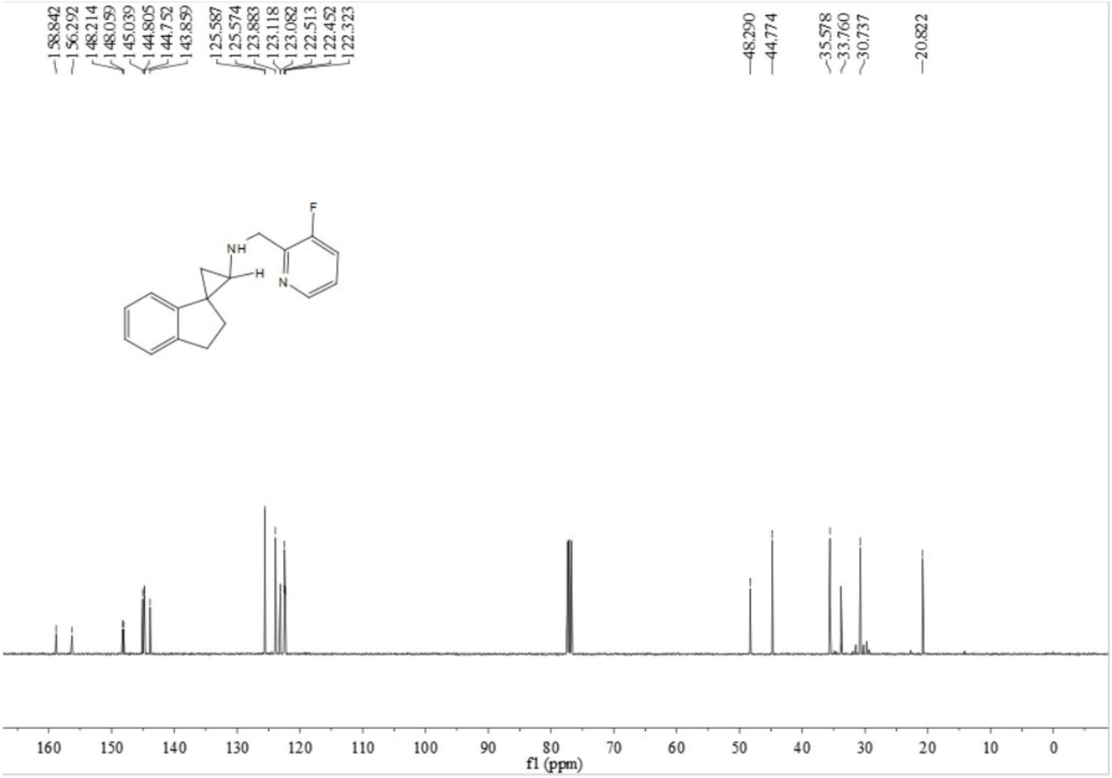

^{1}H NMR (400 MHz, CD_3OD) and ^{13}C NMR (101MHz, CD_3OD) spectrum for 25a

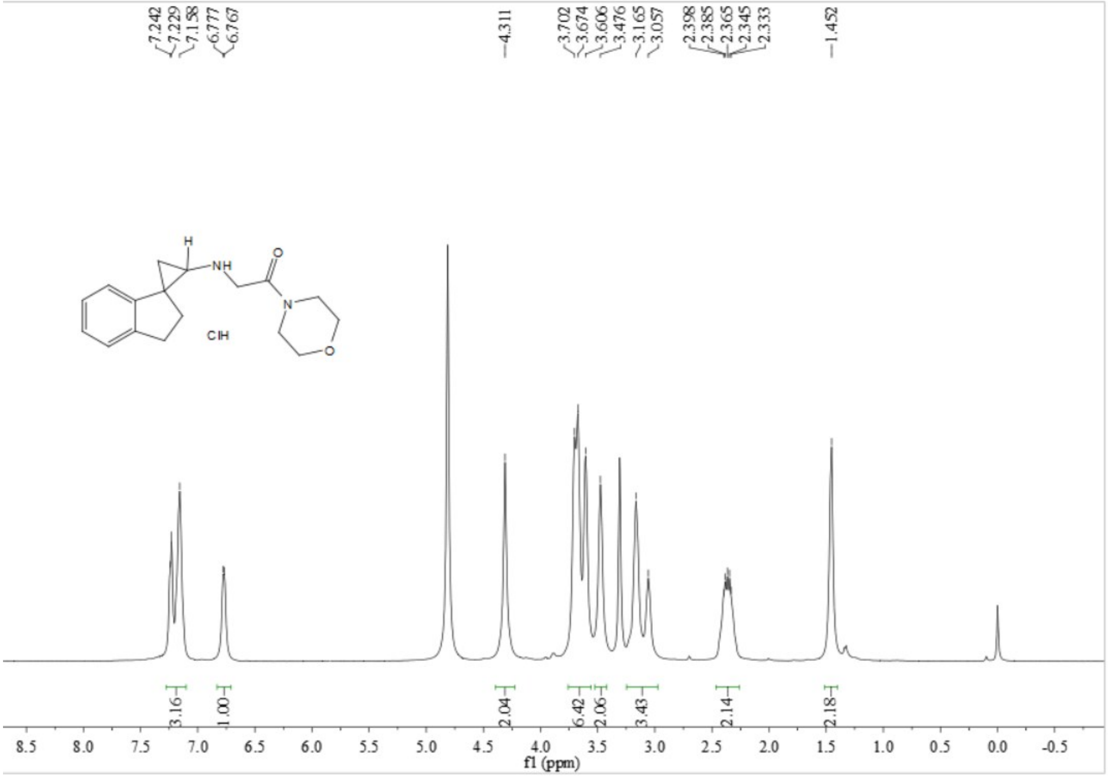

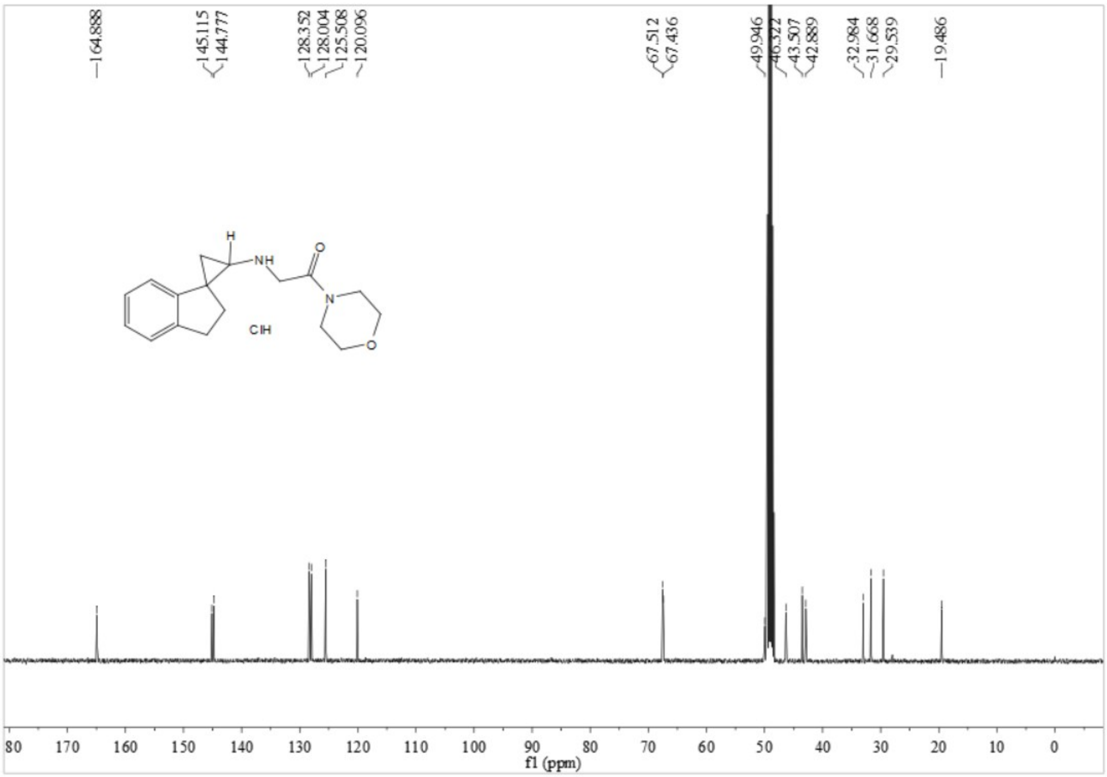

^{1}H NMR (400 MHz, CD_3OD) and ^{13}C NMR (101MHz, CD_3OD) spectrum for 25b

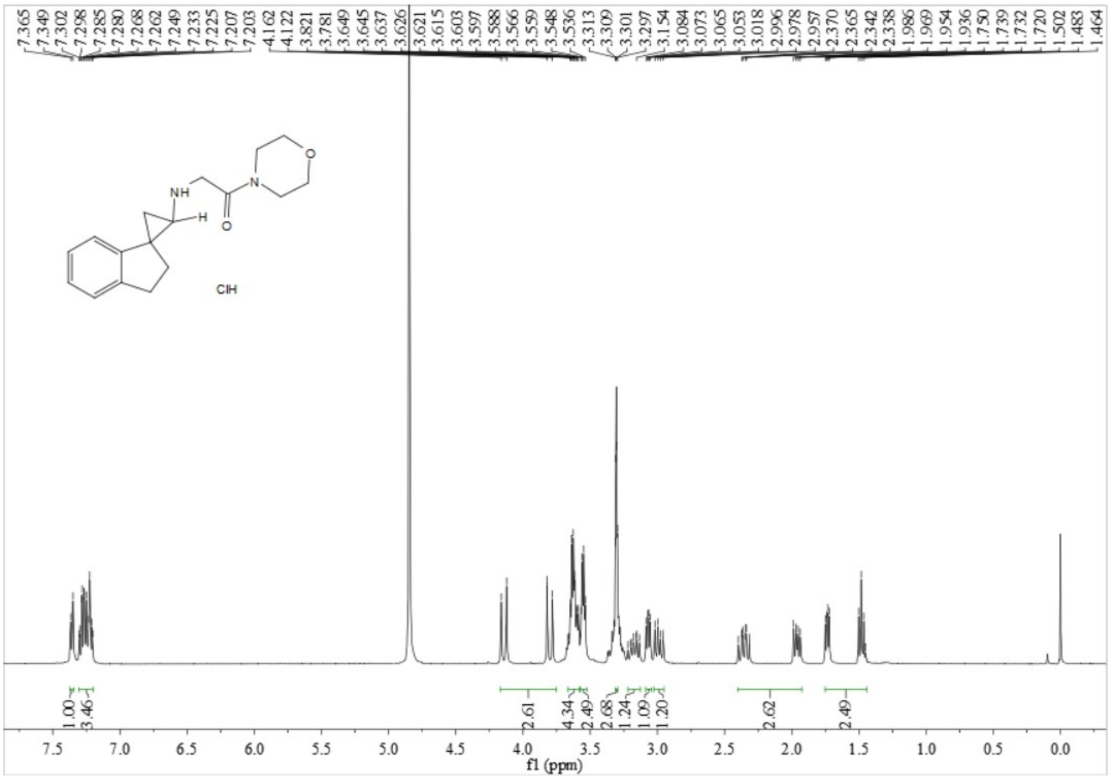

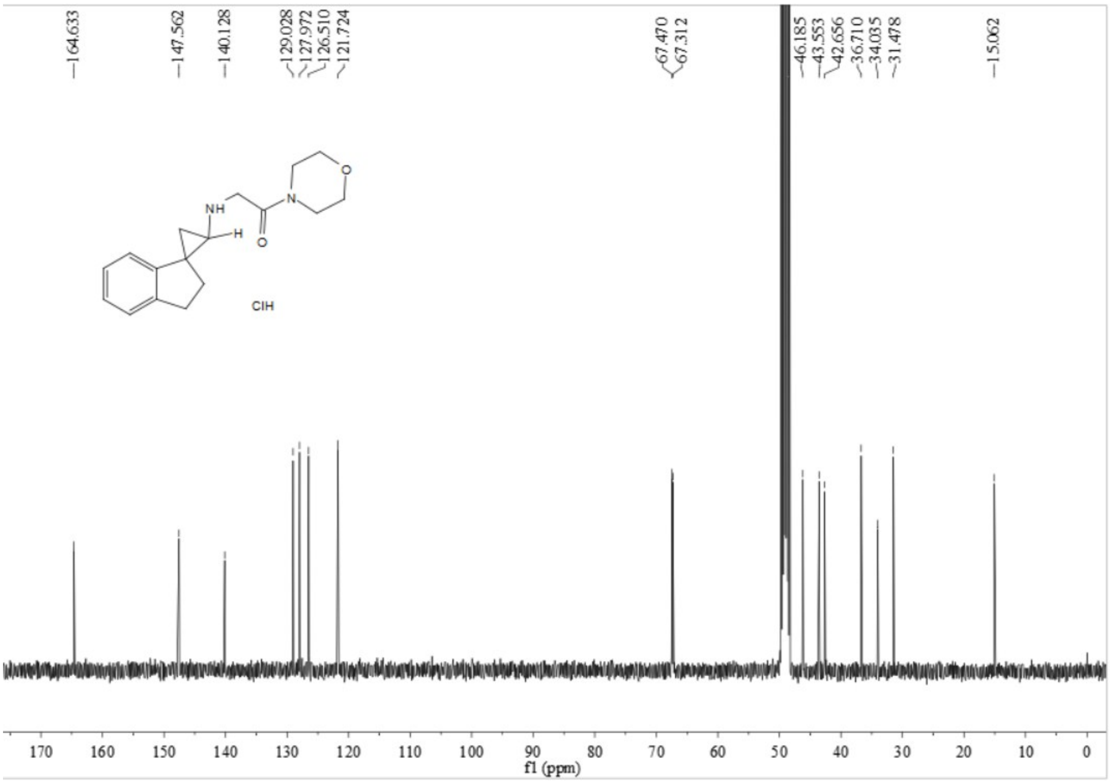

^{1}H NMR (400 MHz, DMSO-*d*6) and ^{13}C NMR (101MHz, DMSO-*d*6) spectrum for 28a

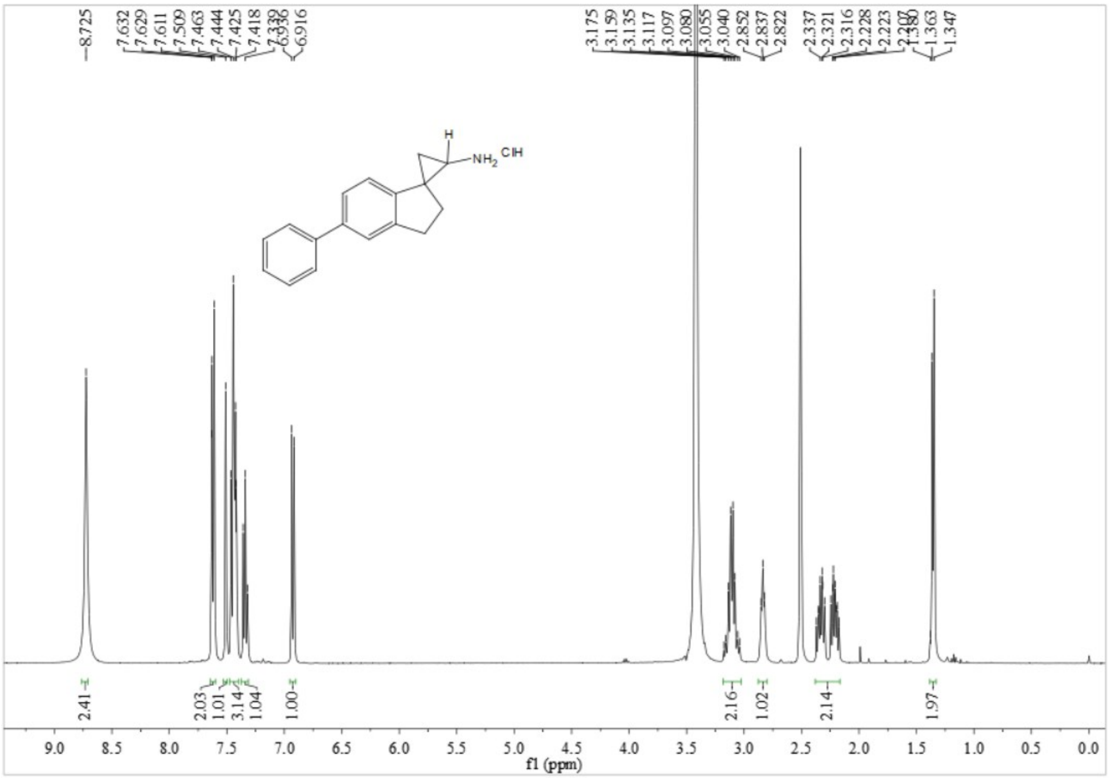

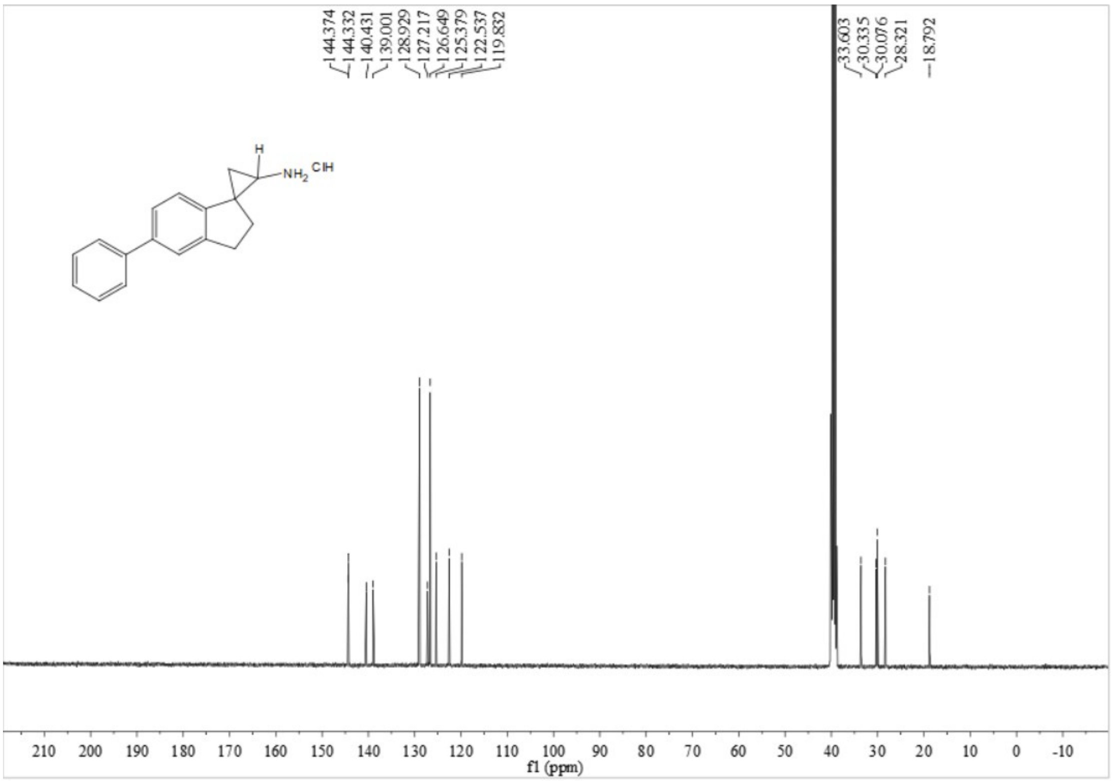

^{1}H NMR (400 MHz, DMSO-*d*6) and ^{13}C NMR (101MHz, DMSO-*d*6) spectrum for 28b

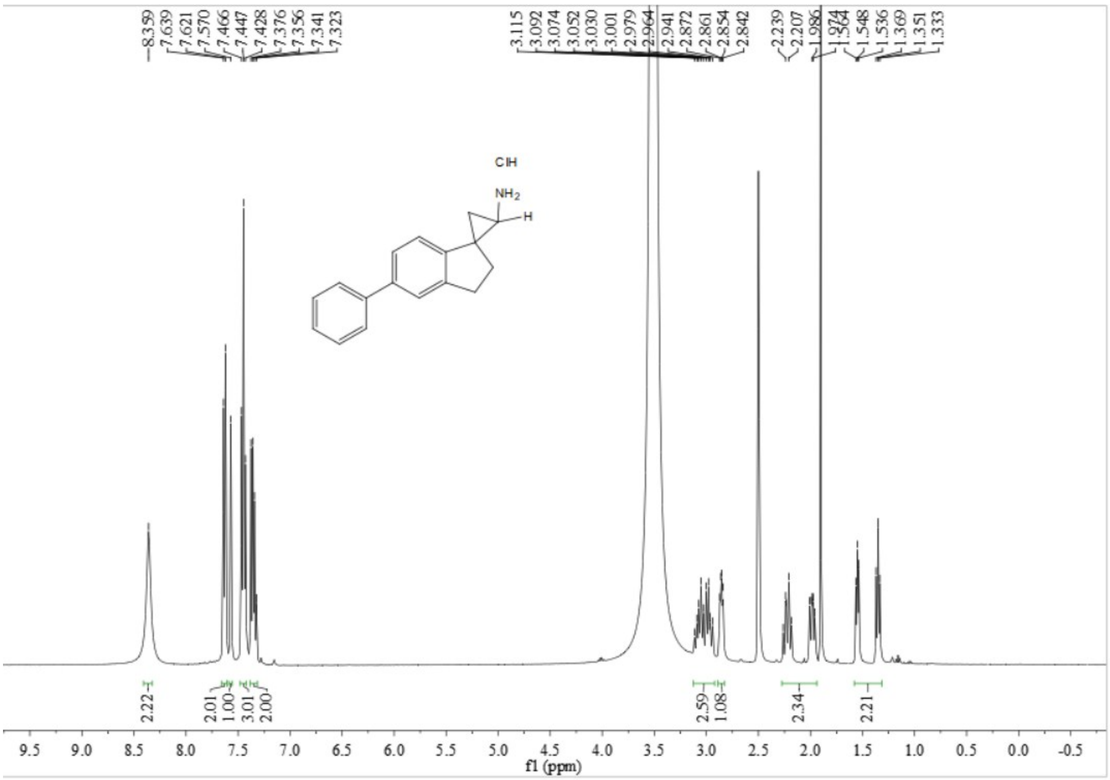

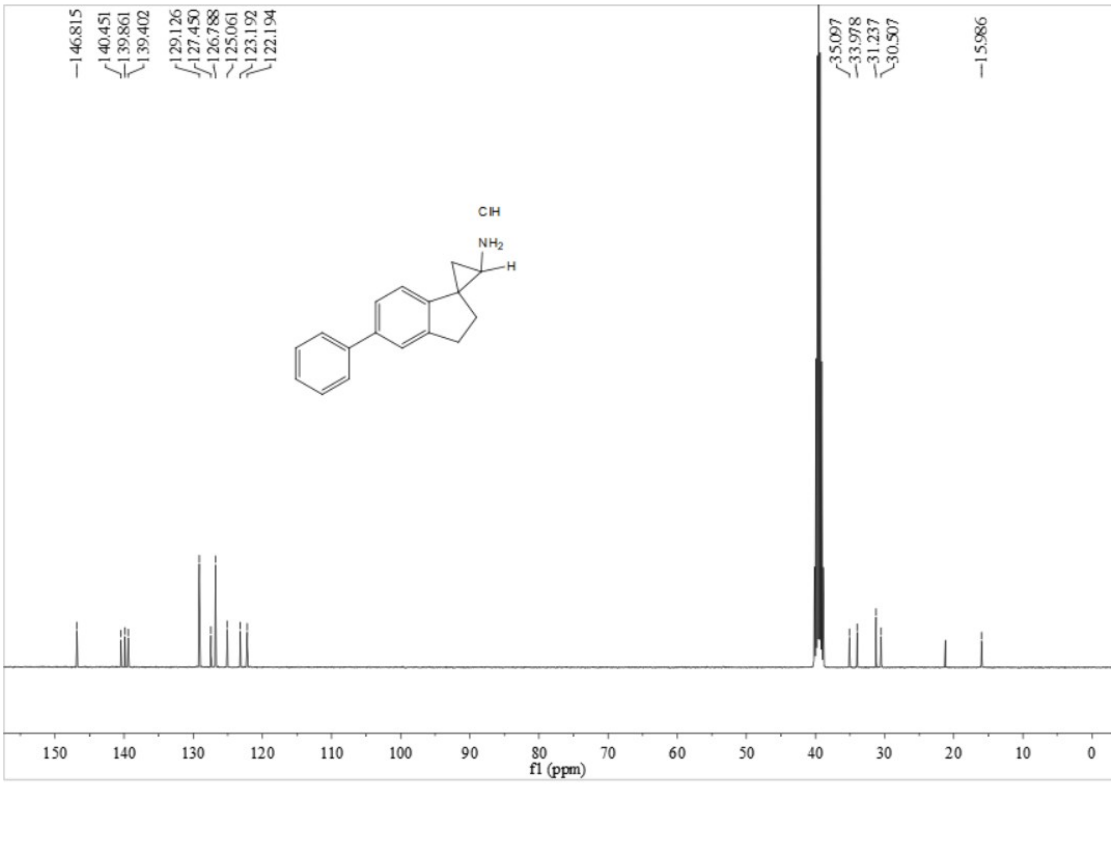

^{1}H NMR (400 MHz, DMSO-*d6*) and ^{13}C NMR (101MHz, DMSO-*d6*) spectrum for 29a

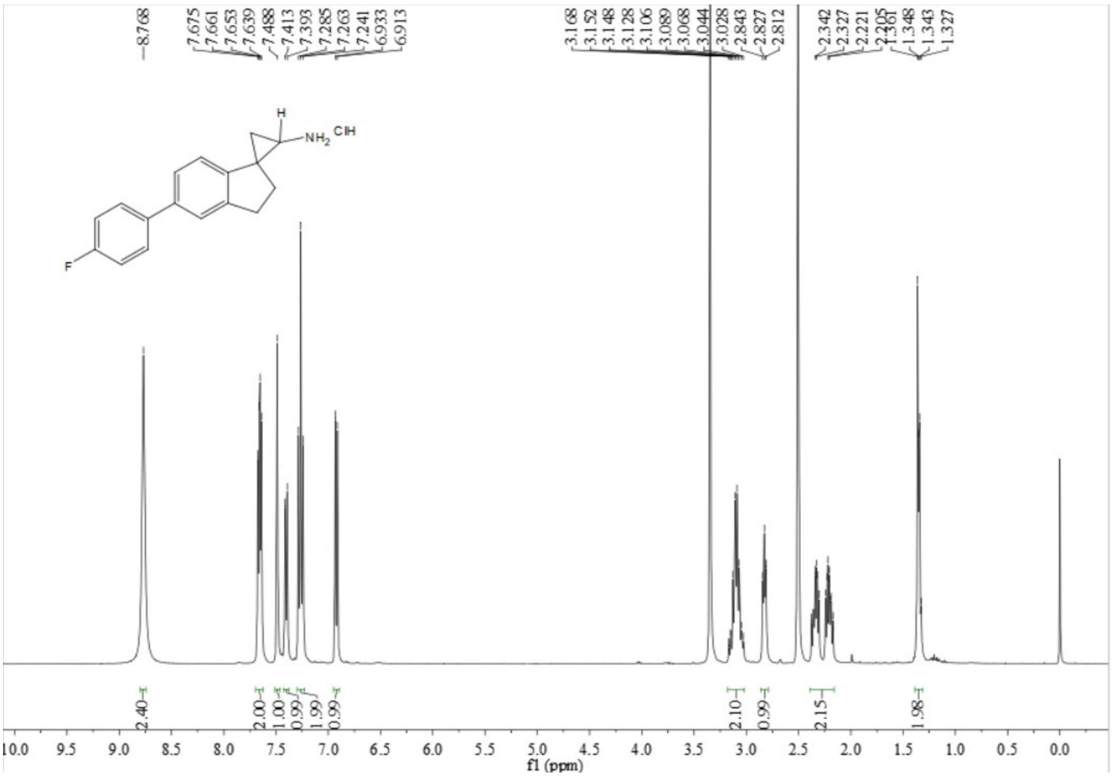

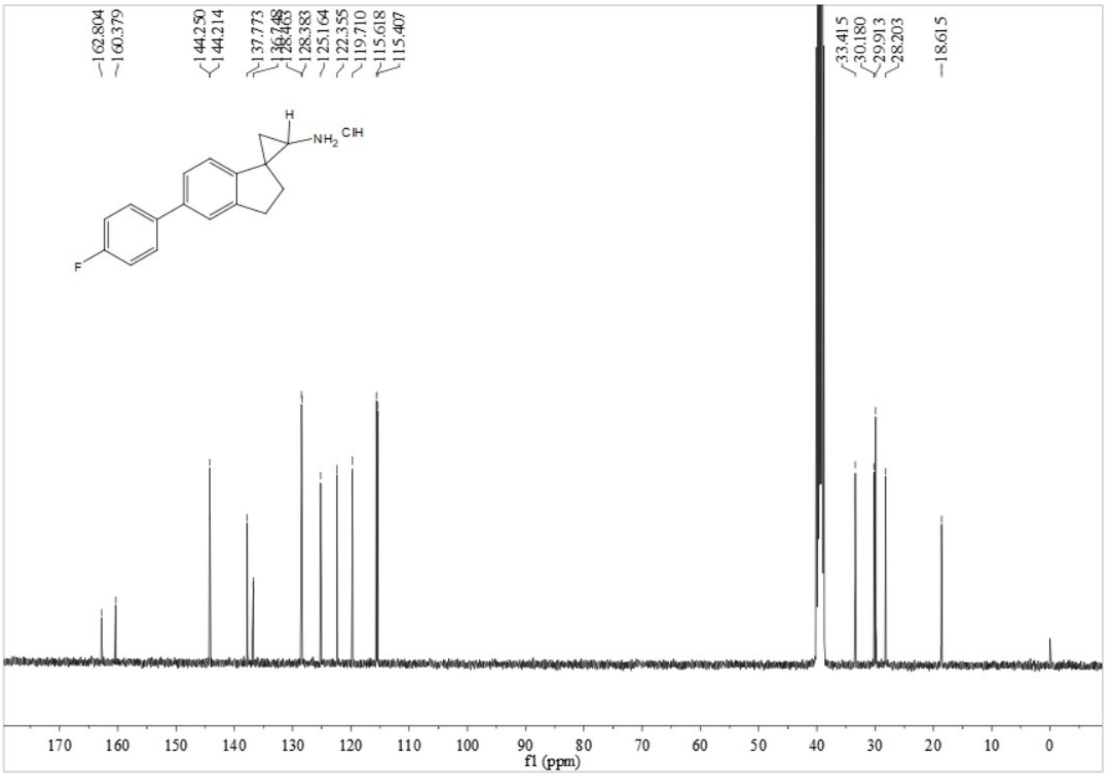

^{1}H NMR (400 MHz, DMSO-*d6*) and ^{13}C NMR (101MHz, DMSO-*d6*) spectrum for 29b

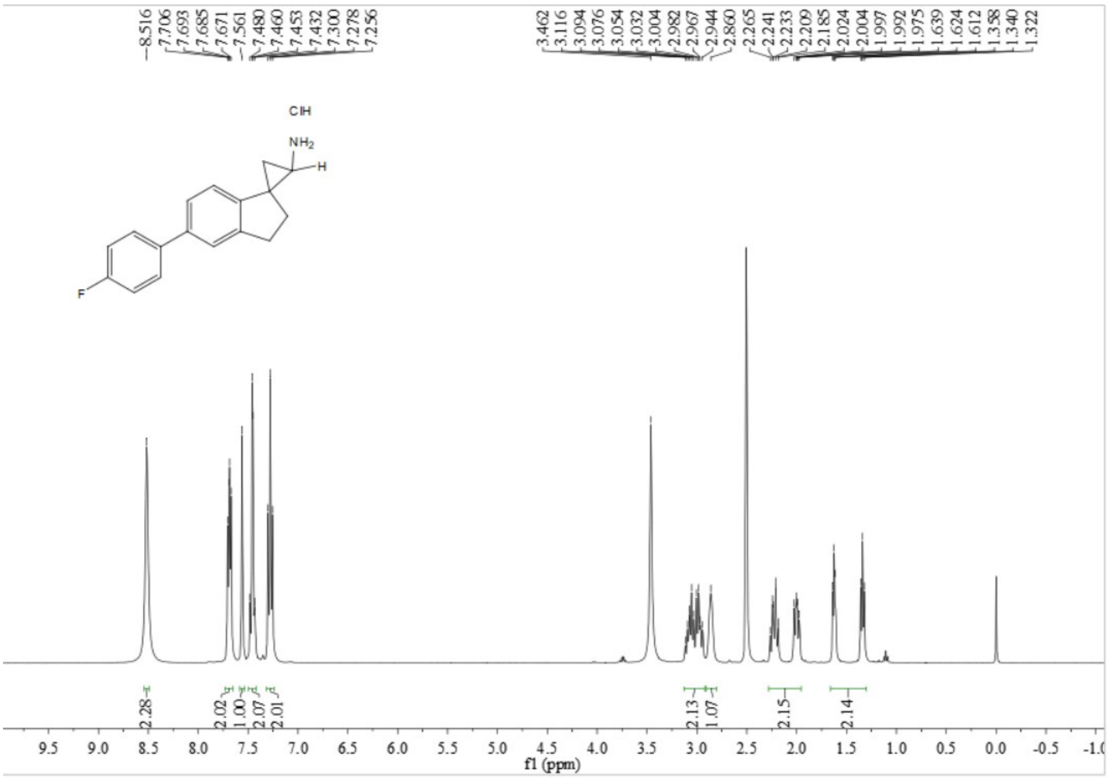

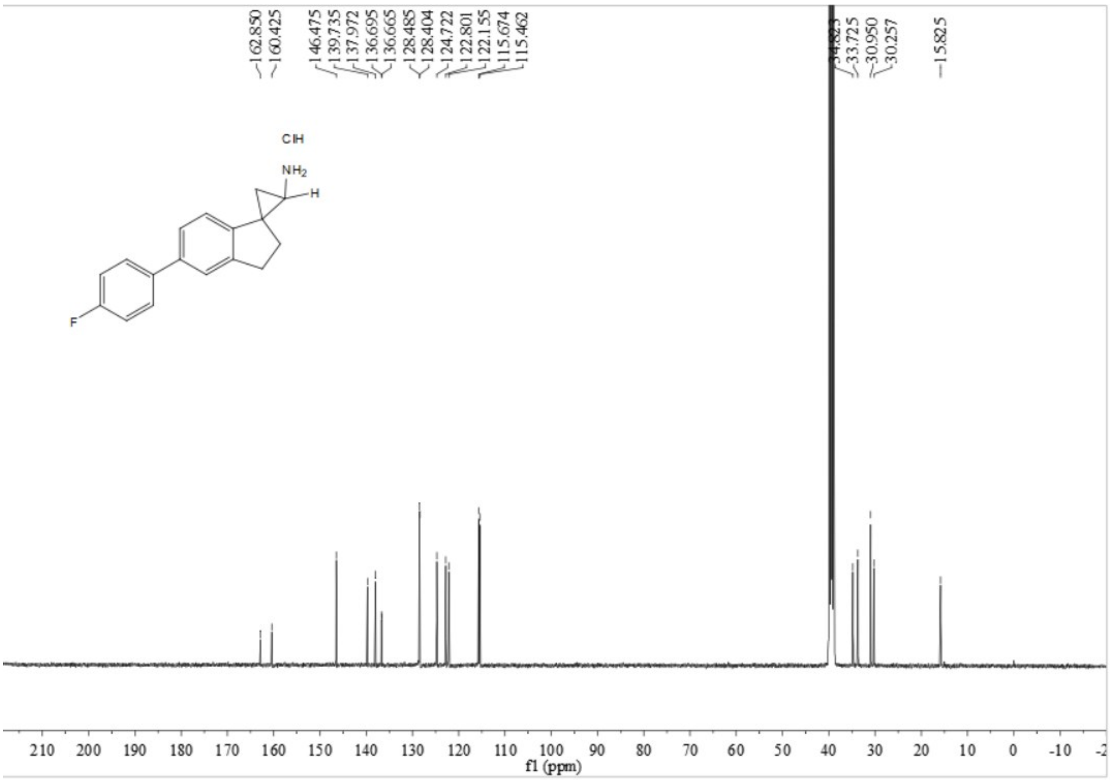

^{1}H NMR (400 MHz, $CDCl_3$) and ^{13}C NMR (101MHz, $CDCl_3$) spectrum for 30a

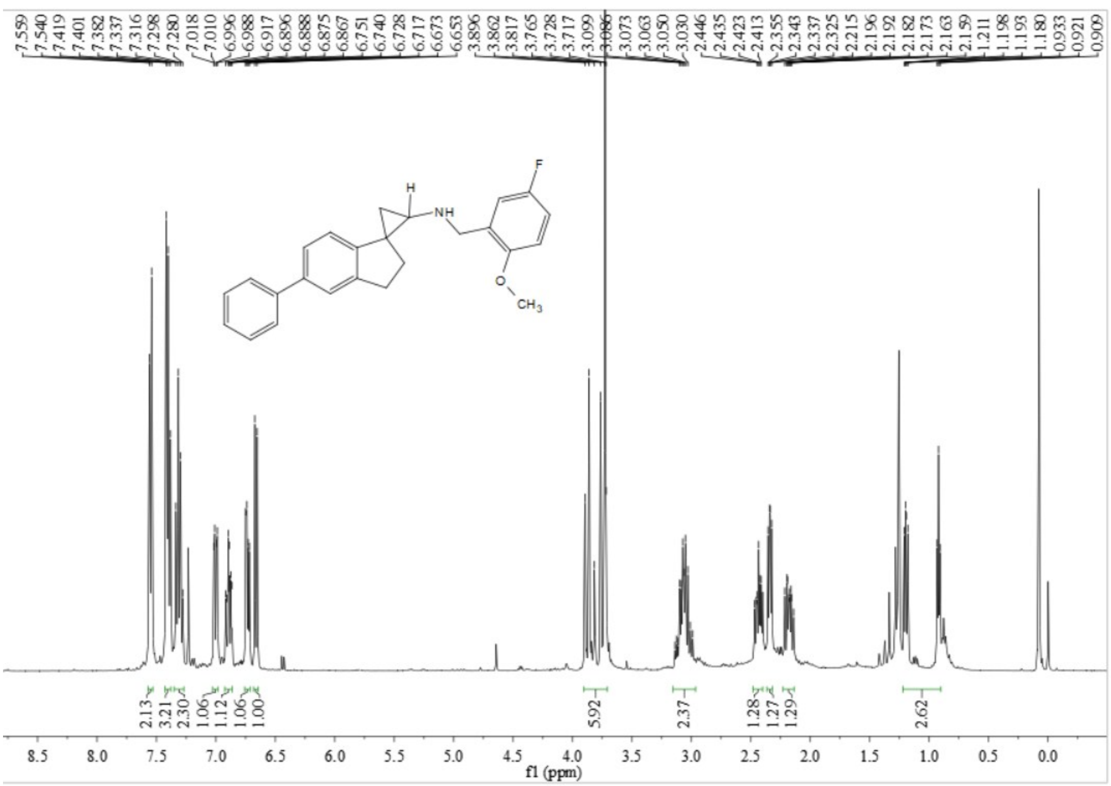

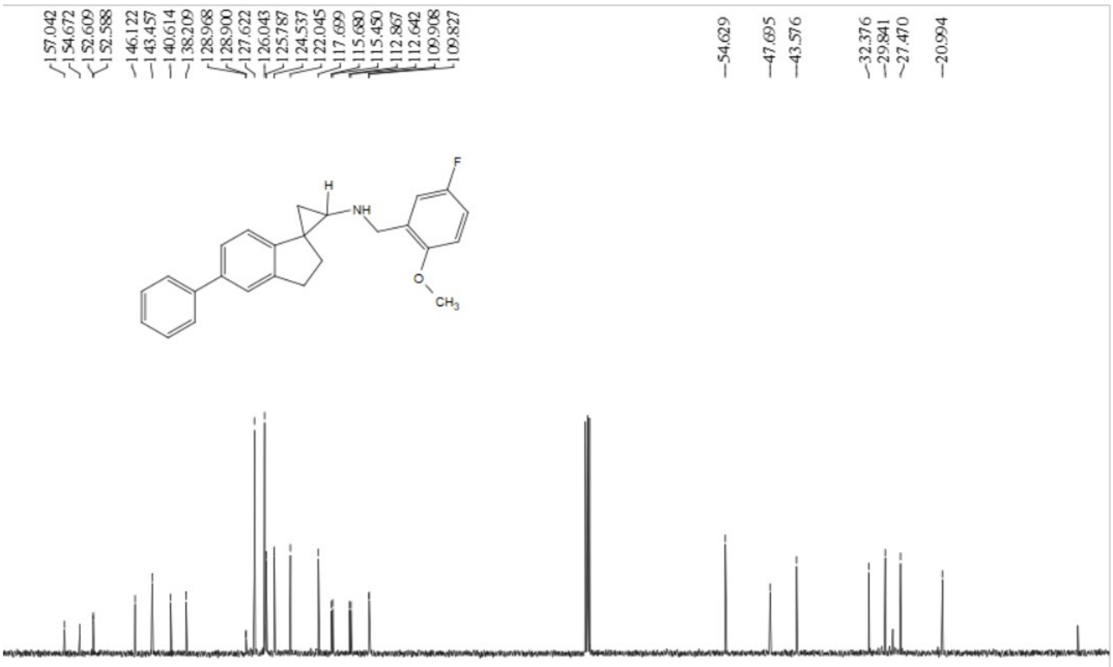

^{1}H NMR (400 MHz, $CDCl_3$) and ^{13}C NMR (101MHz, $CDCl_3$) spectrum for 30b

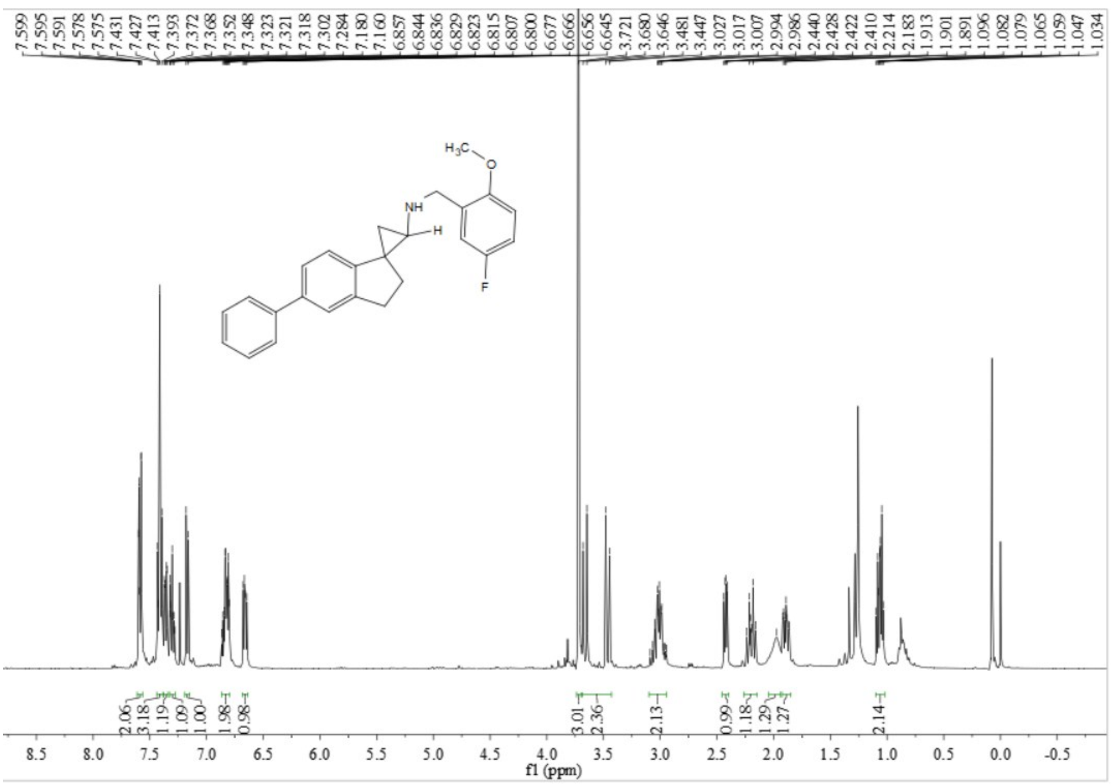

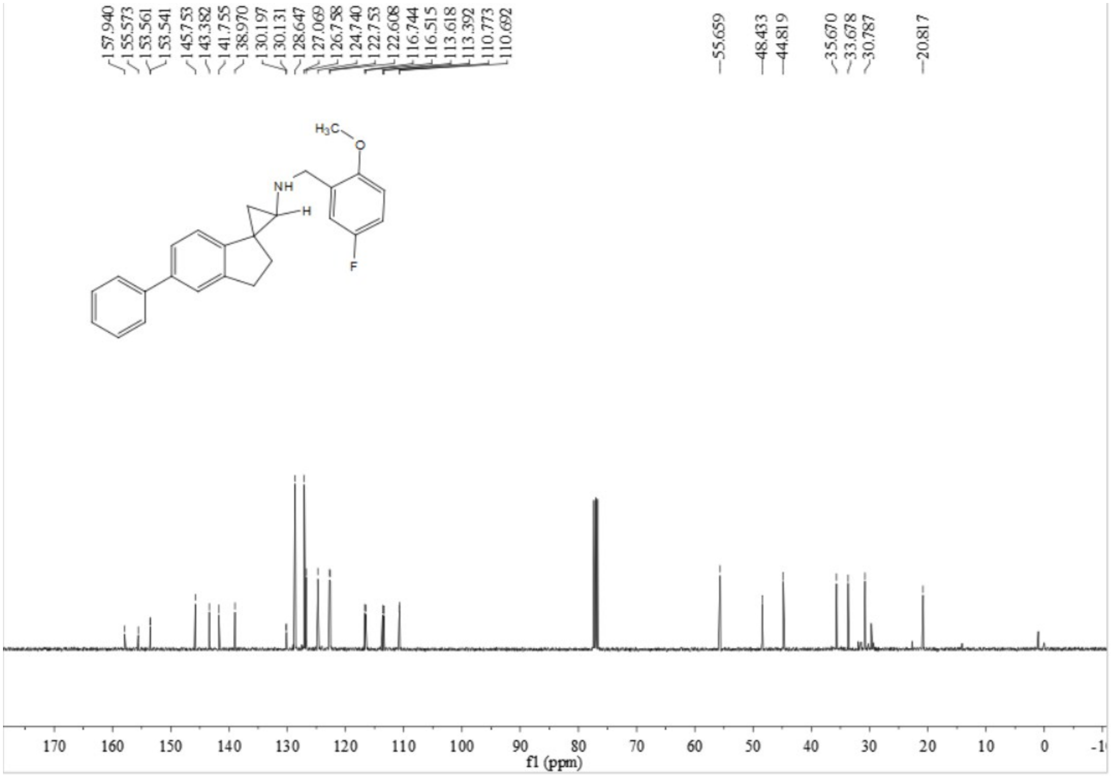

^{1}H NMR (400 MHz, $CDCl_3$) and ^{13}C NMR (101MHz, $CDCl_3$) spectrum for 31a

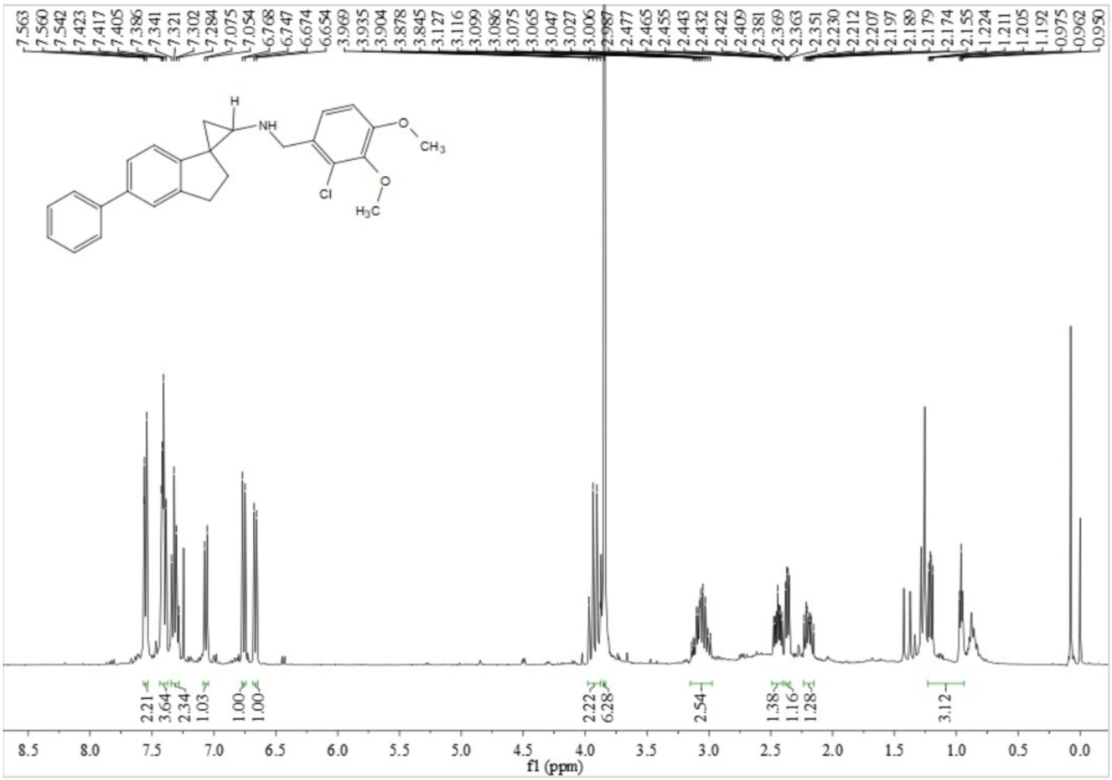

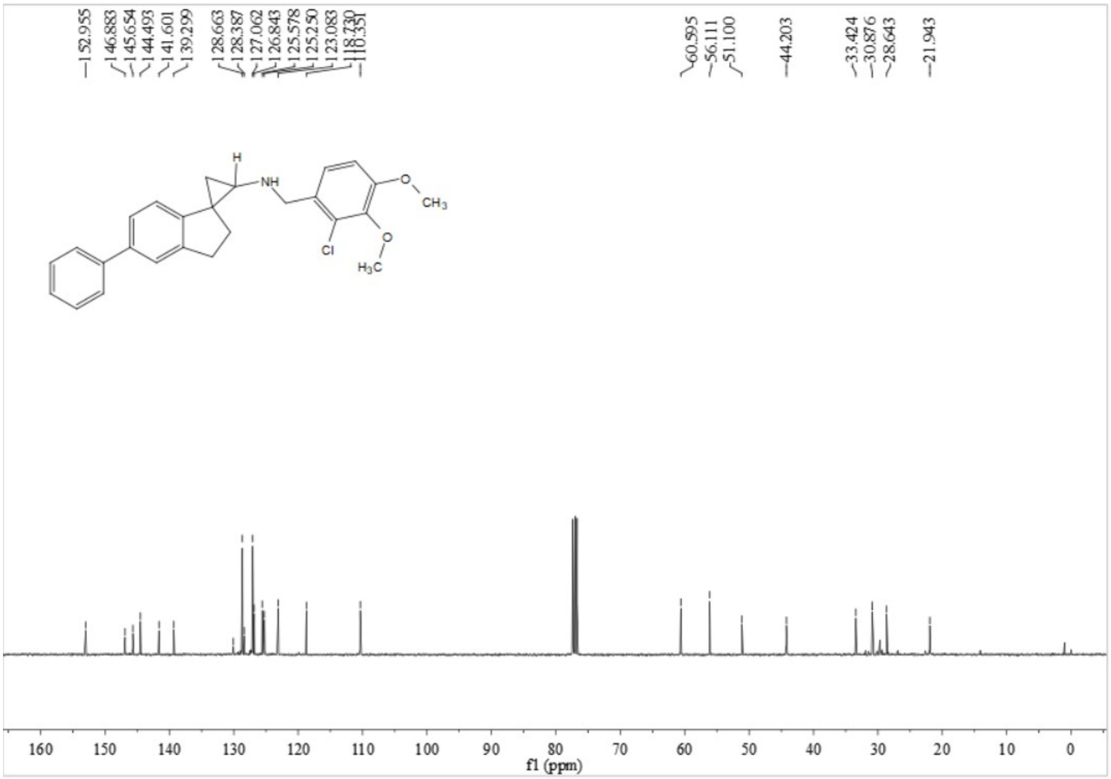

^{1}H NMR (400 MHz, $CDCl_3$) and ^{13}C NMR (101MHz, $CDCl_3$) spectrum for 32a

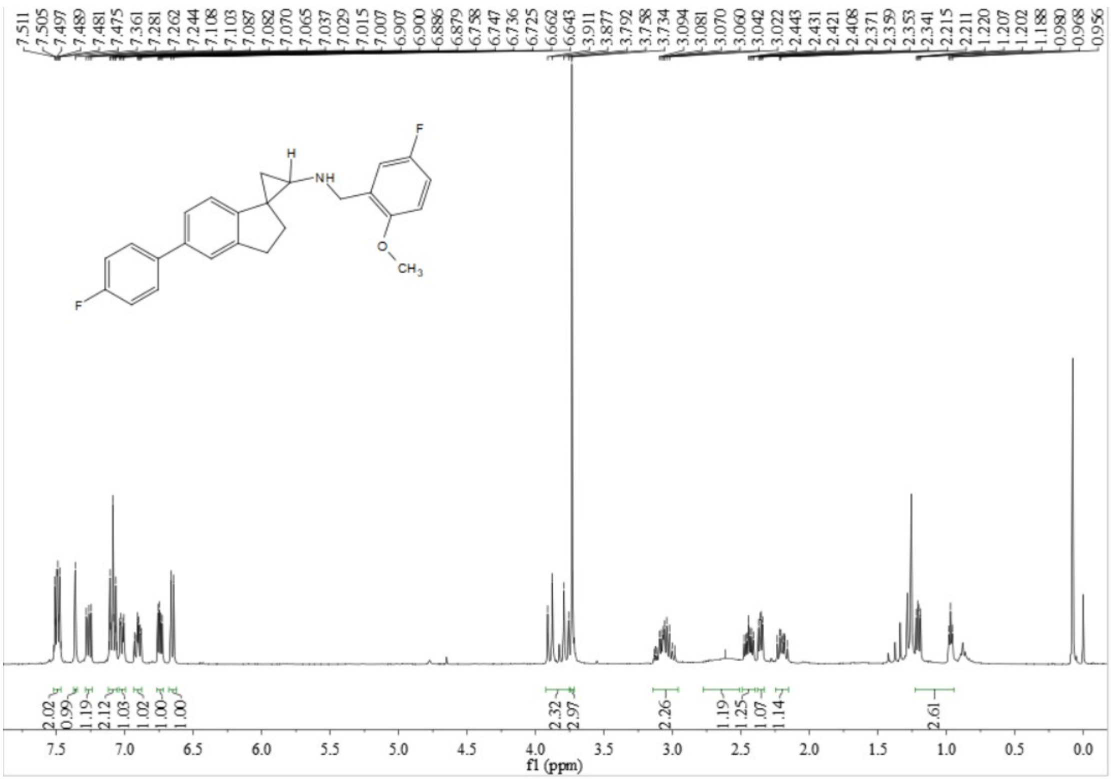

7.511
7.505
7.497
7.489
7.481
7.475
7.361
7.281
7.262
7.244
7.108
7.103
7.087
7.082
7.070
7.065
7.037
7.029
7.015
7.007
6.907
6.900
6.886
6.879
6.758
6.747
6.736
6.725
6.662
6.643
3.911
3.877
3.792
3.758
3.734
3.094
3.081
3.070
3.060
3.042
3.022
2.443
2.431
2.421
2.408
2.371
2.359
2.353
2.341
2.215
2.211
1.220
1.207
1.202
1.188
0.980
0.968
0.956
F
H
NH
O
CH3
F
2.02
0.99
1.19
2.12
1.03
1.02
1.00
1.00
2.32
2.97
2.26
1.19
1.25
1.07
1.14
2.61
7.5 7.0 6.5 6.0 5.5 5.0 4.5 4.0 3.5 3.0 2.5 2.0 1.5 1.0 0.5 0.0
f1 (ppm)

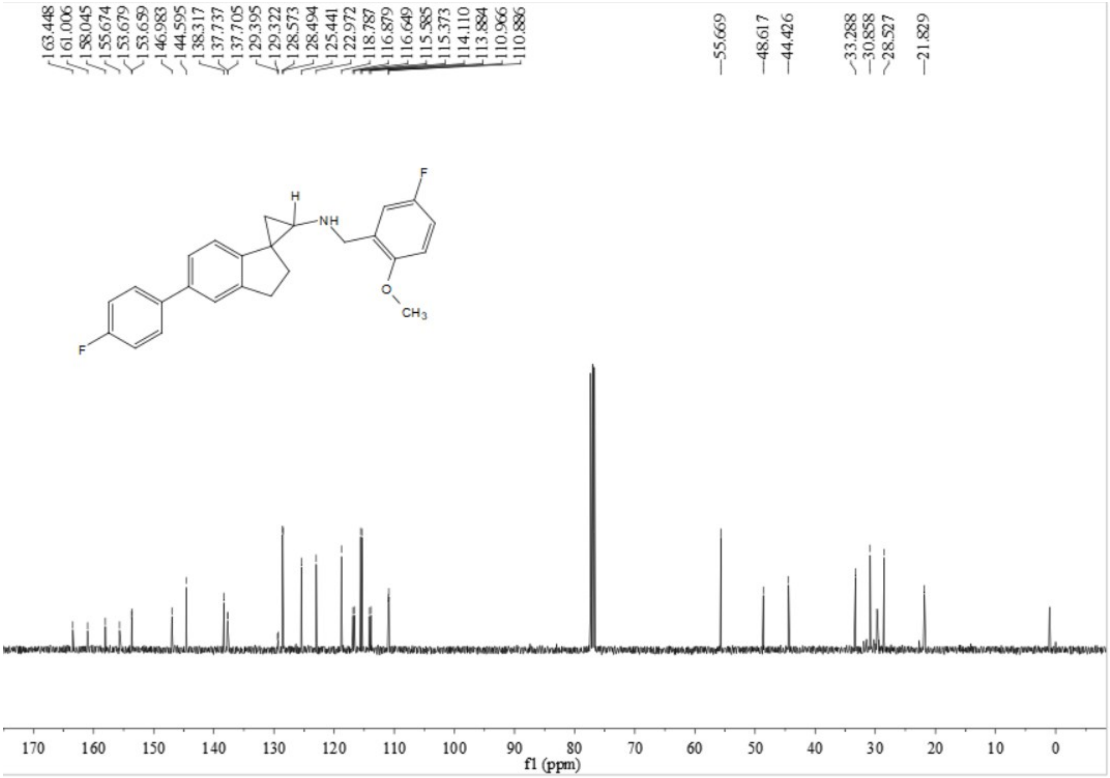

163.448
161.006
158.045
155.674
153.679
153.659
146.983
144.595
138.317
137.737
137.705
129.395
129.322
128.573
128.494
125.441
122.972
118.787
116.879
116.649
115.585
115.373
114.110
113.884
110.966
110.886
55.669
48.617
44.426
33.288
30.858
28.527
21.829
F
H
NH
O
CH3
F
170 160 150 140 130 120 110 100 90 80 70 60 50 40 30 20 10 0
f1 (ppm)
